# Computational design of highly efficient thermostable MHET hydrolases and dual enzyme system for PET recycling

**DOI:** 10.1038/s42003-023-05523-5

**Published:** 2023-11-09

**Authors:** Jun Zhang, Hongzhao Wang, Zhaorong Luo, Zhenwu Yang, Zixuan Zhang, Pengyu Wang, Mengyu Li, Yi Zhang, Yue Feng, Diannan Lu, Yushan Zhu

**Affiliations:** 1 College of Life Science and Technology, Beijing University of Chemical Technology, Beijing, 100029 China; 2 Department of Chemical Engineering, Tsinghua University, Beijing, 100084 China; 3 Beijing Advanced Innovation Center for Soft Matter Science and Engineering, State Key Laboratory of Chemical Resource Engineering, Beijing University of Chemical Technology, Beijing, 100029 China; 4 National Energy R&D Center for Biorefinery, Beijing University of Chemical Technology, Beijing, 100029 China

**Keywords:** Protein design, Biocatalysis

## Abstract

Recently developed enzymes for the depolymerization of polyethylene terephthalate (PET) such as FAST-PETase and LCC-ICCG are inhibited by the intermediate PET product mono(2-hydroxyethyl) terephthalate (MHET). Consequently, the conversion of PET enzymatically into its constituent monomers terephthalic acid (TPA) and ethylene glycol (EG) is inefficient. In this study, a protein scaffold (1TQH) corresponding to a thermophilic carboxylesterase (Est30) was selected from the structural database and redesigned in silico. Among designs, a double variant KL-MHETase (I171K/G130L) with a similar protein melting temperature (67.58 °C) to that of the PET hydrolase FAST-PETase (67.80 °C) exhibited a 67-fold higher activity for MHET hydrolysis than FAST-PETase. A fused dual enzyme system comprising KL-MHETase and FAST-PETase exhibited a 2.6-fold faster PET depolymerization rate than FAST-PETase alone. Synergy increased the yield of TPA by 1.64 fold, and its purity in the released aromatic products reached 99.5%. In large reaction systems with 100 g/L substrate concentrations, the dual enzyme system KL36F achieved over 90% PET depolymerization into monomers, demonstrating its potential applicability in the industrial recycling of PET plastics. Therefore, a dual enzyme system can greatly reduce the reaction and separation cost for sustainable enzymatic PET recycling.

## Abstract (web summary)

A study on the computational design of enzymes for plastic (MHET and PET) degradation yielded an efficient dual enzyme degradation system for PET recycling with a substantially increased activity and a near pure concentration of product TPA.

## Introduction

Synthetic polymers deliver numerous benefits to human society, and plastics have emerged as essential materials for almost every aspect of life. Poly(ethylene terephthalate) (PET) is one of the most widely used synthetic plastics, and over 82 million metric tons are produced globally^1^. However, the degradation of PET is very slow owing to its chemical recalcitrance with regard to abiotic and biological breakdown^2^. Consequently, it accumulates in both landfills and the environment. It therefore causes serious damage to the global ecosystem and intensifies demand for a circular plastic economy^3^. Conventional chemical recycling methods, such as glycolysis^4,5^, hydrolysis^6,7^, alcoholysis^8,9^, and aminolysis^10^, require high temperatures and pressures, toxic catalysts, and hazardous chemicals. Chemical treatment methods introduce numerous unknown impurities that are difficult to eliminate, which increases industrial production costs^1^. Enzymatic depolymerization is an attractive and environmentally friendly alternative method for PET waste recycling because it can be carried out under mild conditions in an aqueous solvent, and therefore complies with the concept of sustainability. Recently developed PET hydrolases such as LCC-ICCG^11^ can achieve 90% PET conversion on an industrial scale. The exceptional selectivity and mild reaction conditions of enzymatic catalysis can be applied to the exclusive depolymerization of the PET in plastic waste into its constituent monomers terephthalic acid (TPA) and ethylene glycol (EG). This greatly reduces processing costs. However, the current problem with the industrial enzymatic recycling of PET is its relatively high cost compared with virgin PET manufacturing^1^. The PET hydrolases LCC-ICCG and FAST-PETase^12^ can quickly depolymerize PET polymers into the short intermediate products bis-2-hydroxyethyl terephthalate (BHET) and mono-hydroxyethyl terephthalate (MHET). However, the catalytic efficiencies of PET hydrolases with regard to the hydrolysis of MHET into the terminal products TPA and EG are moderate^13–15^. The active sites of PET hydrolases are amenable to hydrophobic polymers or short oligo-ethylene terephthalate molecule substrates^16,17^, and always hydrophobic. But, the MHET intermediate tends to bind tightly to the PET-degrading enzyme in a non-catalytically pose. This results in inhibition of the PET-degrading enzymes and low catalytic efficiency with regard to MHET hydrolysis. Therefore, a highly efficient MHET hydrolase is needed for the industrial depolymerization of PET into its pure constituent monomers TPA and EG.

**Fig. 1:**
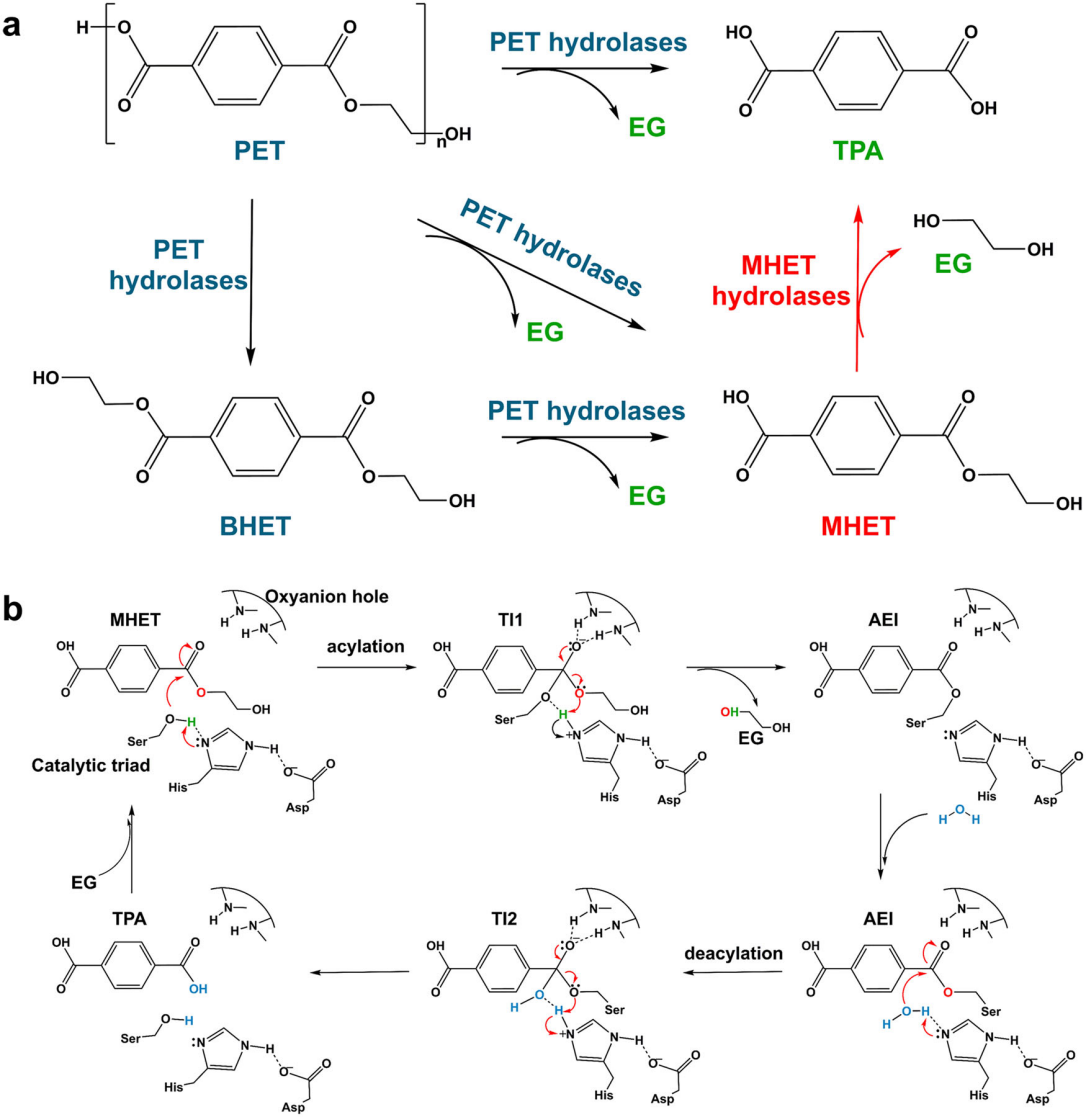
Schematic diagram of PET degradation and catalytic mechanism of MHET hydrolase. **a** Reaction scheme for PET degradation. PET hydrolases depolymerize PET to give MHET as the intermediate product and MHET hydrolases convert MHET to TPA. **b** Reaction coordinate for MHET hydrolysis. The reaction is initiated by the nucleophilic attack of the serine deprotonated by the histidine upon the ester carbon, thus yielding the first tetrahedral intermediate (TI1). The TI1 promotes the formation of an acyl-enzyme intermediate (AEI) and the release of EG. In the deacylation step, another tetrahedral intermediate (TI2) is subsequently generated caused by a nucleophilic attack from a water molecule and then resolved to restore the free enzyme state and release TPA. The red arrow indicates the electron transfer path.

MHET is the major intermediate product of PET degradation (Fig. 1a). However, it inhibits PET hydrolases^13,14,18,19^, resulting in low TPA yield and poor PET conversion efficiency. Multi-enzyme systems can promote the substrate channel and proximity effect between enzymes. This greatly reduce the diffusion restriction between the active centers of the enzymes, thereby promoting enzyme synergy and improving catalytic efficiency^20,21^. In 2016, Yoshida et al.^22^ isolated a bacterium (*Ideonella sakaiensis* 201-F6) that uses PET plastic as a carbon source. This bacterium secretes two enzymes for PET degradation: a PET hydrolase (PETase) and a MHET hydrolase (MHETase). This natural synergistic dual enzyme system efficiently degrades PET into its constituent monomers, i.e., TPA and EG, at 30 °C (Fig. 1a). Knott et al.^23^ covalently connected MHETase to the *N*-terminus of PETase via Gly-Ser linkers to construct a fusion enzyme. That fusion enzyme produced approximately six-fold more degradation products from a PET film in 96 h at 30 °C than the single PETase. The glass transition temperature (*T*_g_) of bulk amorphous PET polymers is in the range of 65–71 °C^24^. When the reaction temperature approaches *T*_g_, the amorphous PET polymer structure transitions from a glassy state to a rubbery state^25^, which helps the active sites of PET hydrolases contact more ester bonds in the polymer substrate^24^. Therefore, high temperatures approaching the *T*_g_ of PET are necessary for its degradation^24^, and great progress has been made to enhance the thermostability of PETase^3,16,17,26–36^. Recently, guided by machine learning, a PETase variant with five point mutations termed FAST-PETase^12^ has been engineered to efficiently degrade a wide range of post-consumer PET plastics at an optimal temperature of 50 °C. The activity of this enzyme exceeds the activities of other engineered alternatives^11,26,29^. However, there is no report of a thermostable MHETase to match this PET hydrolase at 50 °C (Table S1).

Various efforts have been made to find appropriate MHET hydrolases to engineer dual enzyme systems that remove the intermediate product MHET^21,23,37–40^. The removal of MHET improves the collective efficiency of PET depolymerization and promotes the TPA yield. Mrigwani et al.^39^ constructed a dual enzyme system comprising *Thermus thermophilus* carboxylesterase (TTCE) and leaf-branch compost cutinase (LCC), which is a PET hydrolase. The system produced a 30% higher pure TPA yield from the degradation of PET films at 60 °C than LCC alone. Haugwitz et al.^40^ added the engineered TfCa mutant WA to PETase PM, and the resulting system achieved an 11-fold TPA yield from the degradation of an amorphous PET film compared with the single PETase PM at 45 °C over 24 h. However, although they are thermostable, these MHET hydrolases always confer low MHET degradation activity, and are therefore incompatible with proficient and thermostable PET hydrolases, unless used in excess. Therefore, it is necessary to develop efficient thermostable MHET hydrolases to engineer viable dual enzyme systems for industrial PET recycling.

Recently, a structure-based computational method has been used to design new enzymes for various applications^41–52^. Computational enzyme design uses virtual screening; it can significantly reduce laboratory work and greatly shorten the development cycle required for new enzymes, in contrast to directed evolution technology^50,53^. In the present research, we used our computational enzyme design tool PRODA^54–58^, in combination with high-throughput molecular dynamics simulation^51,59–62^ (Fig. S1), to generate efficient enzymes for MHET hydrolysis at the optimal temperatures required for the PET hydrolase FAST-PETase. An active site model for MHET hydrolysis was developed based on the catalytic mechanism of the serine hydrolases, which catalyze a two-step reaction involving the formation of an acyl-enzyme intermediate (acylation) that is released hydrolytically in the second step (deacylation)^63^. During acylation, the key catalytic serine is deprotonated by His, activating the hydroxyl oxygen of Ser for nucleophilic attack on the carbonyl C of MHET, which releases EG and forms the acyl-enzyme intermediate (Fig. 1b)^64,65^. During the deacylation step, the ester carbon of the acyl-enzyme intermediate is nucleophilically attacked by a water molecule. His plays a similar role as in acylation, deprotonating the catalytic water and transferring the proton to the serine, then activating the release of TPA^64,65^. Based on the above mechanism, we constructed an active site model for obtaining efficient MHET hydrolases by PRODA. The designed MHET hydrolases were fused with the PET hydrolase FAST-PETase with the aid of the protein structure prediction tool AlphaFold^66,67^ to engineer a dual enzyme system for PET depolymerization. The dual enzyme system exhibited higher PET conversion rates and TPA yields than the single enzyme system.

## Results

### Thermostable scaffold selection from structural database for MHET hydrolysis

**Fig. 2:**
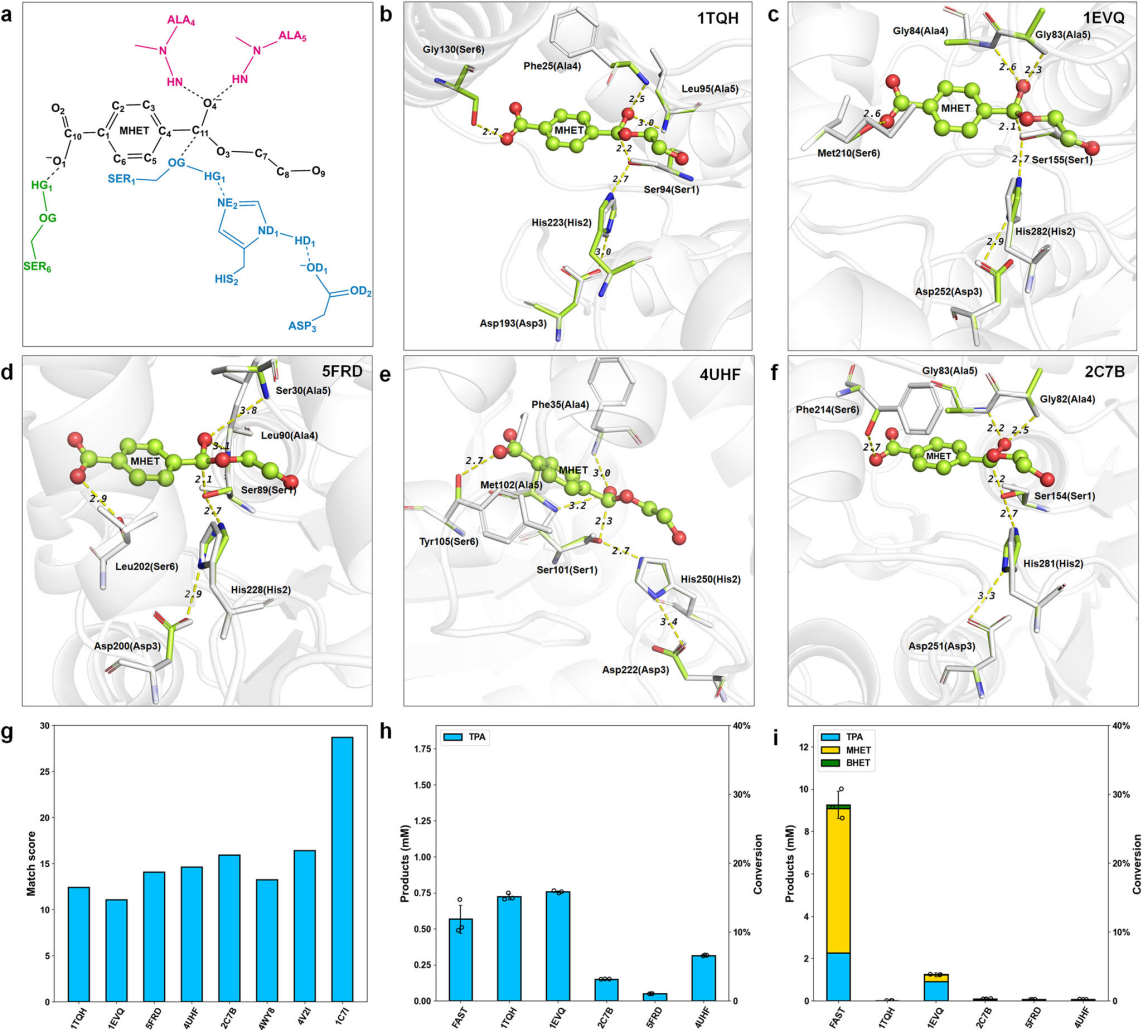
Scaffold selection and characterization for MHET hydrolysis. **a** The active site model for MHET hydrolysis. The catalytic triad residues are colored in blue, the residues as hydrogen bond donors for the oxyanion are colored in pink, and the residue stabilizing the TS is colored in green. **b**–**f** Overlay of the matched active site model(colored) with the crystal structure(white) in five scaffolds: 1TQH(b), 1EVQ(c), 5FRD(d), 4UHF(e), and 2C7B(f), respectively. The protein structures are shown as cartoon and colored in white. The TS of MHET is shown in ball-and-stick model. The matched results of the active site are shown in licorice and colored in green, while the crystal structures in licorice and colored in white. Hydrogen bonds are shown in dashed yellow lines, around which the numbers indicate the distances (Å). **g** The computed match penalty score of 8 scaffolds. **h** Relative activity towards MHET hydrolysis of the selected scaffolds and FAST-PETase. Enzyme reactions were performed in triplicate over 10 min at 50 °C using 4.8 mM MHET and 0.25 μM enzyme loading in 50 mM sodium phosphate, pH 7.5. **i** PET degradation activity test by the selected scaffolds and FAST-PETase on post-consumer bottle grade PET powder (Pc-PET, 200–500 μm) over 24 h at 50 °C in triplicate, using 0.5 μM enzyme loading, 0.16% Pc-PET substrate loading, and 100 mM sodium phosphate, pH 8.0.

The constructed active site model for MHET hydrolysis is shown in Fig. 2a. In addition to the catalytic core—the Ser-His-Asp catalytic triad—the backbone nitrogen of two alanine groups is used to donate hydrogen bonds to stabilize the oxyanion of the transition state (TS) of MHET (Fig. 2a). Alanine is selected in the minimal active site model because it can be replaced by other amino acids except for proline. A serine provides a hydrogen bond to the carboxyl group of the TS to facilitate its binding. The catalytic geometrical relationships between catalytic residues and the transition state for active-site matching are shown in Table S3. The distance and angle parameters are determined based on the crystal structures of natural MHETase (PDB ID: 6QGA^64^ and 6JTT^68^), and the complex structures of serine proteases and their transition state analogs^69^.

To find a suitable protein scaffold to accommodate the active site for MHET hydrolysis at high temperatures, our active site matching algorithm, ProdaMatch^54,57,58^, was used to select scaffolds from a thermophilic scaffold library owing to its natural backbone stability advantage^70^. The thermostable hydrolase library consisted of a total of 104 enzyme entries (Table S2), which were collected from the Protein Data Bank (PDB) according to the following criteria: (i) can be expressed in E. coli; (ii) have a high-resolution crystal structure; and (iii) appropriate sequence length. More details are provided in the method section. As a result, a total of 32 matches were identified in 8 unique scaffolds. Based on the reported optimal temperatures and the match scores of these 8 scaffolds (Fig. 2g and Table S4), 5 top-ranked thermostable scaffolds (1EVQ, 1TQH, 5FRD, 4UHF, and 2C7B) were screened out for the experimental test. The superposition of the 5 matched results with their crystal structures is shown in Fig. 2b–f. The calculated transition state geometries of the catalytic triad and oxyanion hole residues in 5 scaffolds almost overlapped with those in the crystal structures, where the root-mean-square deviations (RMSDs) of the catalytic triads (i.e., 1TQH: 0.663 Å; 1EVQ: 0.666 Å; 5FRD: 0.154 Å; 4UHF: 0.162 Å; and 2C7B: 0.217 Å) reached sub-angstrom accuracies and all hydrogen bonding distances in the matched geometries were within 2–4 Å of each other (Fig. 2b–f). This suggests that these 5 scaffolds are able to accommodate the constructed active site model while maintaining perfect catalytic geometric constraints and possibly afford MHET degradation activity. All these five scaffolds were expressible, and two scaffolds, i.e., 1EVQ and 1TQH, exhibited apparent activity with regard to MHET hydrolysis (Fig. 2h). Their activities were 1.27 and 1.33 times higher than that of the FAST-PETase (Fig. 2h). The experimentally measured activities of scaffolds 1EVQ and 1TQH were consistent with their matching scores (Fig. 2g), and they had the lowest penalty scores among all the test scaffolds. Moreover, the 1TQH scaffold had almost no activity with regard to PET degradation compared with 1EVQ (Fig. 2i), whose PET degradation activity reached only 1/7 that of FAST-PETase, indicating that the 1TQH scaffold conferred higher specificity with regard to the hydrolysis of MHET. Therefore, the 1TQH scaffold was selected for further redesign.

### Computational design of efficient variants for MHET hydrolysis

**Fig. 3:**
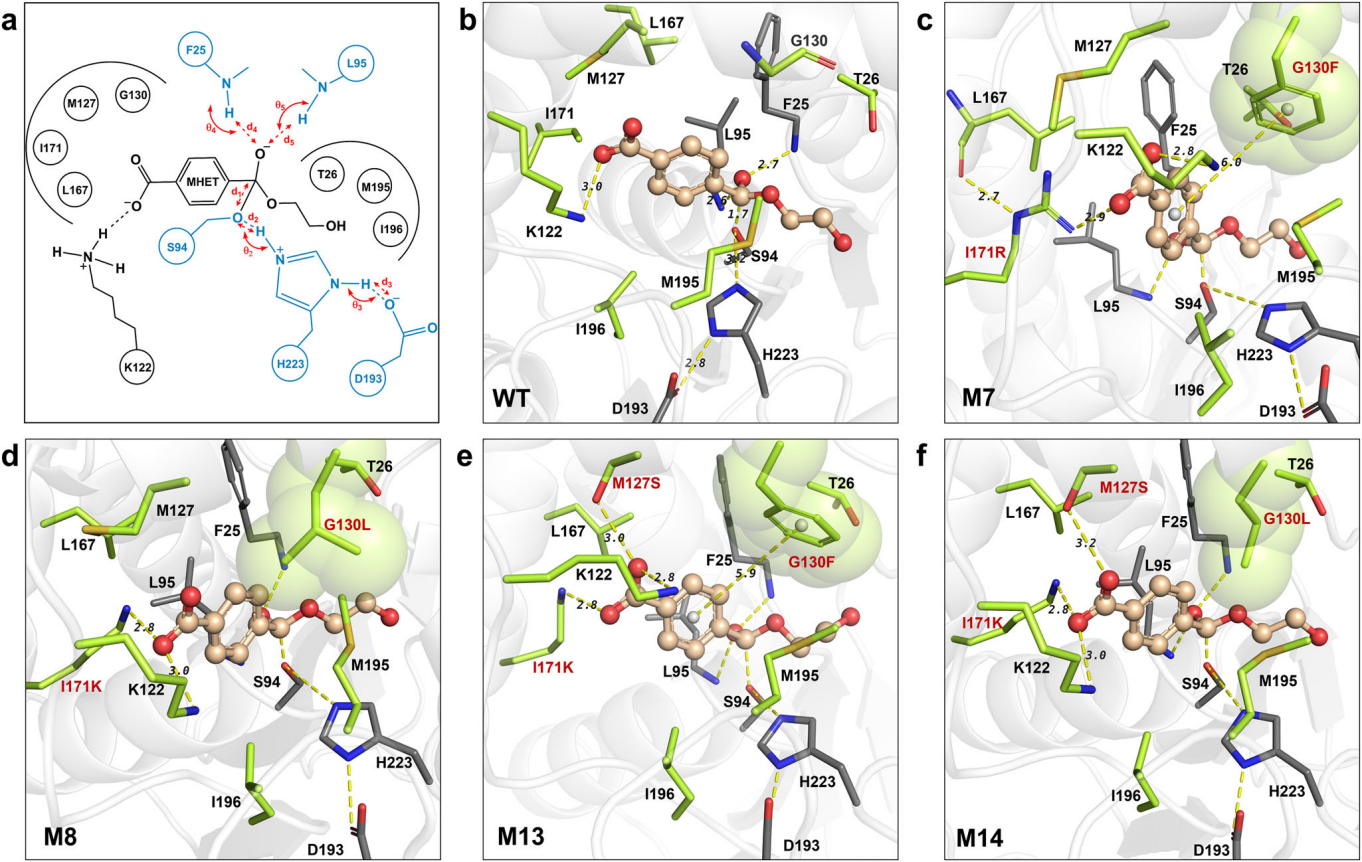
Computed binding geometries of wild type Est30 and designed variants. **a** The catalytic geometrical constraints in active site redesign model. The catalytic residues are shown in blue and the design residues in black. **b** wild type Est30; **c** M7(I171R/G130F); **d** M8(I171K/G130L); **e** M13(I171K/G130F/M127S); **f** M14(I171K/G130L/M127S). The protein structures are shown as cartoon and colored in white. The TS of MHET is shown in ball-and-stick model and colored wheat, while residues are shown in stick model. The sequence selection residues are colored in lemon, while the catalytic triad and oxygen anion hole residues are colored in gray. The hydrogen bonds are shown in yellow dashed lines, around which the numbers indicate the distances (Å). The mutations are labeled in red. The computed binding geometries of other experimentally confirmed variants can be found in Fig. S5.

The 1TQH scaffold corresponds to a thermophilic carboxylesterase cloned from *Geobacillus stearothermophilus* named Est30^71^. To obtain variants with increased activity with regard to MHET hydrolysis, it was necessary to redesign the active site of Est30 to stabilize the TS conformation of MHET in the acylation step. The TS conformation of MHET formed a tetrahedral intermediate (TI) and was used to establish a computational model (Fig. 1b, TI1 (the first tetrahedral intermediate)) for the redesign of Est30. A rotamer library of the TS with 946 conformers was generated following the placing rules (Table S5) and the catalytic geometrical constraints (Table S6) using the targeted small molecule placement approach developed in our earlier work^55,72^. That approach accurately represented the translational, rotational, and conformational freedoms of the TS in the active site of Est30. The computed binding geometry (Fig. 3b) of the TS in wild-type (WT) Est30 showed that the substrate MHET was exposed on the outside of the pocket, with aliphatic residues I171, M127, and L167 surrounding the carboxylic group of MHET. The carboxyl terminus of MHET formed a weak salt bridge with K122 (Fig. 3b, the H-bond distance was 3.0 Å and the angle was unfavorable). Our redesign strategy was to stabilize the catalytically productive conformation of the TS by constructing a hydrogen bonding network to tightly bind the carboxyl terminus of the TS and enhance the hydrophobic environment surrounding the benzene ring of MHET. In total 26 residues in the 10 Å range around the substrate MHET were selected as design sites. The design scheme is shown in Table S7 and the locations of design sites is shown in Fig. S2, where the amino acids at 8 sequence change positions (T26, K122, M127, G130, L167, I171, M195, and I196) were varied among (AILMFWYVCSTKHRNQ). In addition, 18 positions, including 5 catalytic residues (catalytic triads S94/H223/D193, and oxyanion holes F25/L95) and those surrounding the active site, were subjected to side-chain conformational optimization. Figure 3a shows the geometric constraints between catalytic residues and substrate in the TS state and the positions of the redesigned sites relative to the substrate. Using our computational enzyme design tool PRODA^56^, a total of 704 sequences, containing 104 single, 400 double, and 200 triple mutant sequences were generated, and ranked according to the energies of the enzyme–TS complex system (Tables S8–10). More than 70% of the sequences (512/704) had a lower binding energy than the WT. Here, PRODA was used to identify a small set (704) of variants corresponding to the global or near global minimum energy conformations on the energy landscape from the huge sequence space (20^8^). The representative mutations, I171R, I171K, and M127S, which introduced additional hydrogen bonding with the carboxyl terminus of MHET, and mutations G130L and G130F, which enhanced hydrophobic stacking with the benzene ring of MHET, are shown in Fig. 3c–f. To identify a number of tractable sequences for the experimental test, high-throughput molecular dynamics (MD) simulations were invoked to eliminate potential false-positive sequences which refer to computational sequences that are judged to be more active than the design template by the calculated free energies but fail in experiments.

**Fig. 4:**
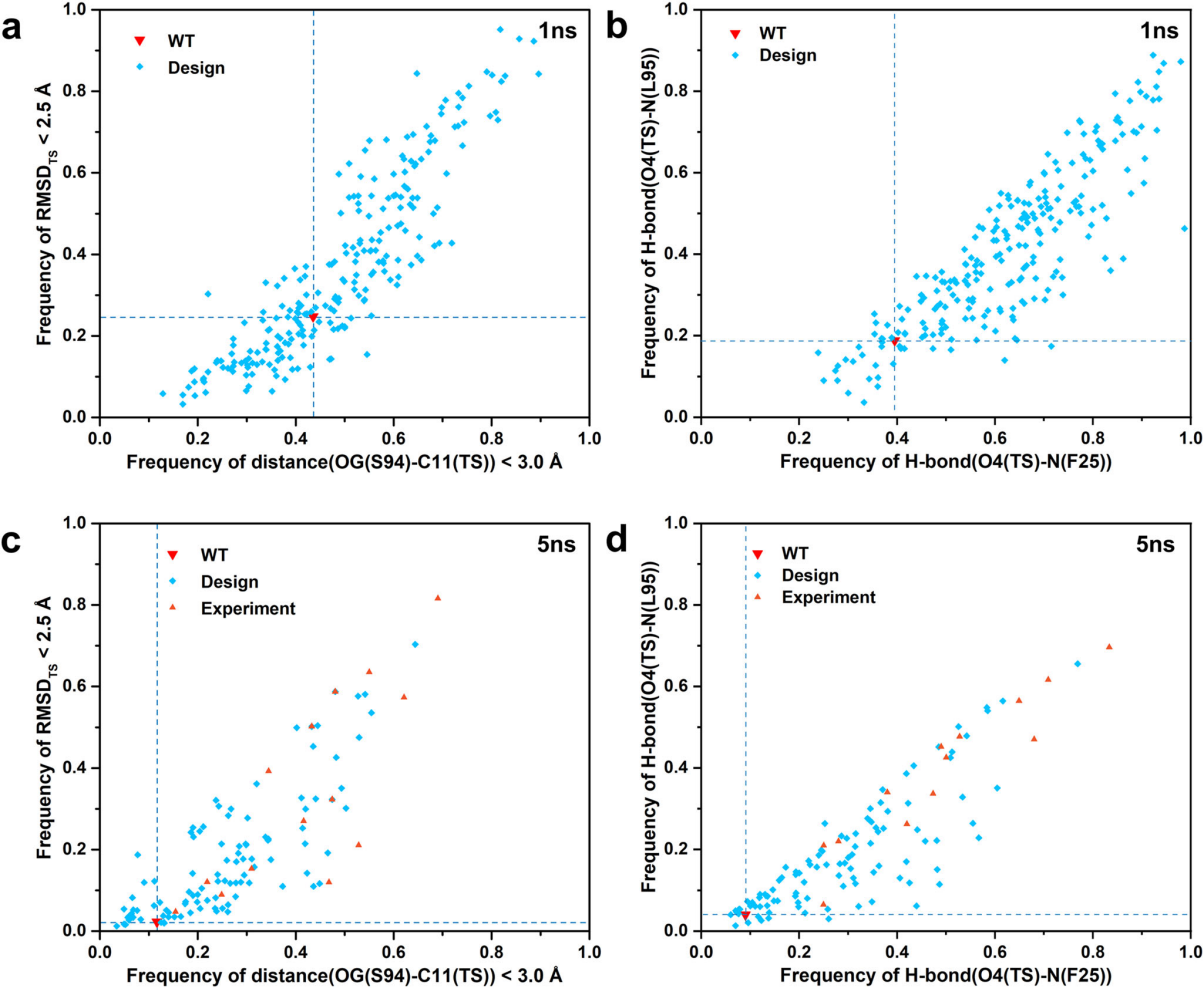
Evaluation of predicted variants based on two rounds of multiple short-time MD simulations. **a**, **b** Scatter plots of four catalytic indicators collected from ten 1 ns MD simulations trajectories conducted for the sequences generated by PRODA. **c**, **d** Scatter plots of four catalytic indicators collected from five 5 ns MD simulations trajectories conducted for the sequences screened by ten 1 ns MD simulations. Each symbol represents one designed variant. The catalytic indicator frequency of RMSD_TS_ < 2.5 Å is calculated with the first frame in the MD simulation trajectory as the reference. The hydrogen bonding criteria used in the catalytic indicators are defined as: the bond length (Donor-Acceptor) is less than 3.5 Å and the bond angle (Donor-H-Acceptor) is greater than 120 °. A graphical illustration of the catalytic indicators can be found in Fig. S3. Details can be seen in Tables S11, S12.

Based on the selection criteria, a total of 237 sequences were further virtually screened through two rounds of high-throughput MD simulations. The selection criteria were: (i) the calculated binding energy change ΔΔG_bind_ < 0 kcal/mol and the folding energy change ΔΔG_fold_ < 8 kcal/mol or the total energy change ΔΔG_total_ ranked in the top 30 among the sequence set; (ii) diversity of mutations (Fig. S4); and (iii) inspection of the computed binding geometries. MD simulations were used to evaluate the sequences produced by PRODA to eliminate the potentially false positive predictions. Four catalytic indicators (defined in Table S11, Fig. S3) were developed based on the catalytic mechanism of serine hydrolases^63,65^, including the RMSD of the TS, the distance of the nucleophilic attack, and the strength of the two oxyanion hole hydrogen bonds. The catalytic indicators were used to monitor the massive and high-dimensional MD simulation data for all 237 selected sequences and the WT Est30 (Fig. 4). The four catalytic indicators calculated based on 10 × 1 ns (10 independent 1 ns) MD simulations for 237 sequences are presented in Fig. 4a, b. In total, there were 118 sequences in the intersection set of the first quadrant of Fig. 4a and the first quadrant of Fig. 4b, which implies simultaneous improvement of the four catalytic indicators compared with the WT. Therefore, these sequences were selected for further validation by using longer MD simulation. The four catalytic indicators calculated based on 5 × 5 ns (5 independent 5 ns) MD simulations for 118 sequences are presented in Fig. 4c, d. Most of the sequences (94/118) produced simultaneous improvement compared with the WT according to the four catalytic indicators. This states that 60% (143) of the sequences produced by PRODA calculation (247) were identified as potential false positive sequences by the high-throughput MD simulations and eliminated. Finally, considering both the PRODA calculations and the MD simulations, a total of 14 variants were selected for experimental assessment according to the following ranking criteria: (i) a lower calculated binding energy change ΔΔG_bind_ and a lower folding energy change ΔΔG_fold_; (ii) the mutational statistical significance values of all sequences calculated by PRODA; (iii) the top-ranked catalytic indicators in the MD simulation; (iv) the sequence diversity of the variants; and (v) inspection of the catalytic conformation in the MD simulation trajectory. The top-ranked 14 in silico designs are labeled out in Fig. 4c, d, and they were subjected to further experimental validation.

### Characterization of the activity, stability, and structure of each in silico-designed variant

**Fig. 5:**
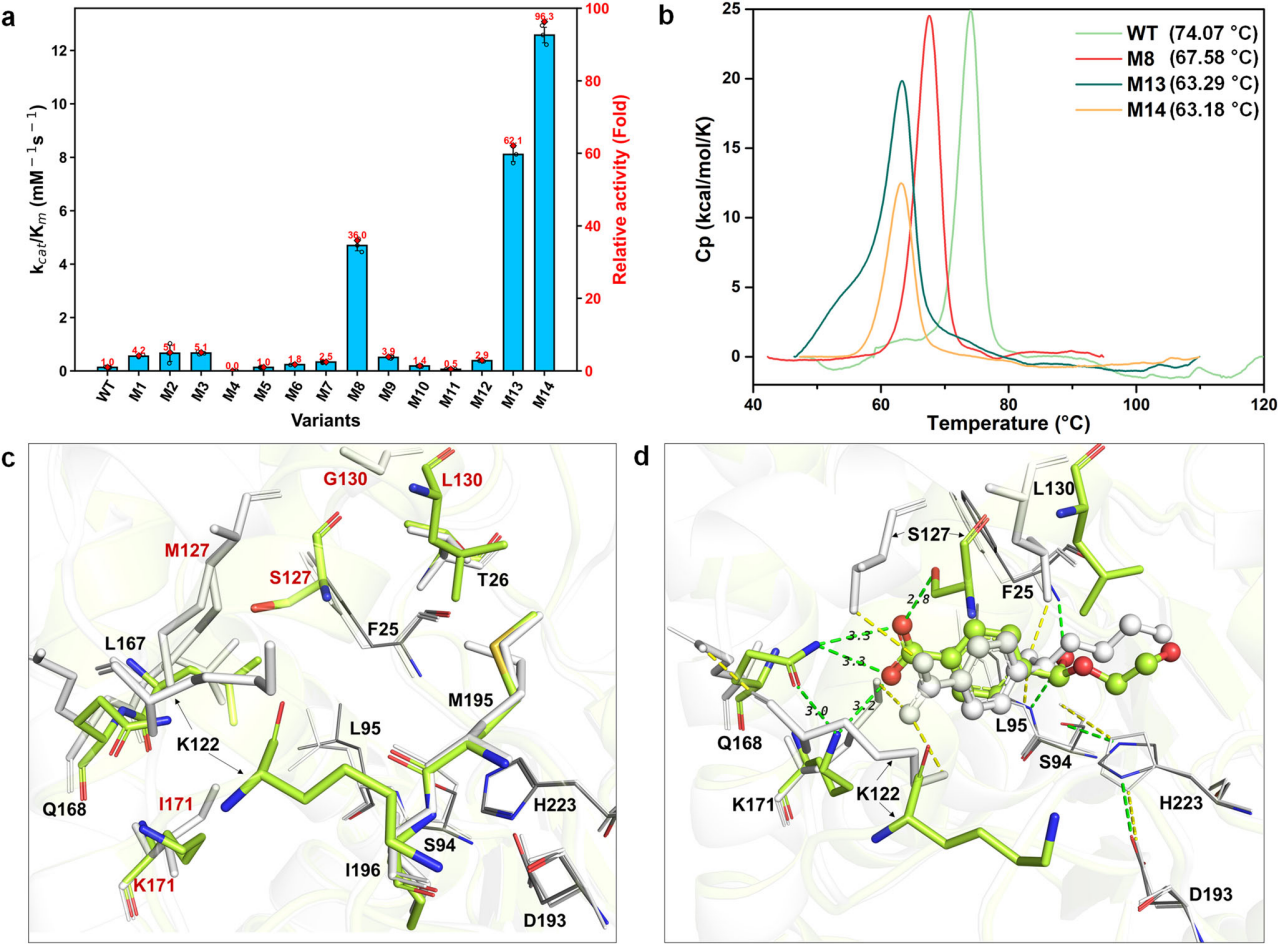
Characterization of activity, stability, and structure of wild type Est30 and designed variants. **a** Catalytic efficiency of Est30 and the designed variants towards MHET hydrolysis at 50 °C. The reactions were performed in triplicate over 10 min with 0.5 μM purified enzyme in 50 mM sodium phosphate, pH 7.5. The activity fold of designed variants relative to WT is shown in right ordinate and labeled with red numbers. **b** Calorimetric enthalpy curves of WT and the designed variants M8, M13, and M14 by DSC. Samples were heated from 30 to 120 °C at a rate of 3 °C per min during the measurement. **c** Crystal structure alignment between wild type Est30 (PDB Code:1TQH, colored white) and the variant M14(I171K/G130L/M127S) (PDB Code: 8ILT, colored lemon). **d** Computed binding geometries of variant M14 based on wild type structure 1TQH (colored white) and variant structure 8ILT (colored lemon). The protein structures are shown as cartoon. The binding sites are shown in stick model and the catalytic triad and oxyanion hole residues are shown as lines. The TS of MHET is shown in ball-and-stick model. The hydrogen bonds are shown in green and yellow dashed lines, around which the numbers indicate the distances (Å). The mutations are labeled in red.

The kinetic parameters of the WT carboxylesterase Est30 and 14 in silico-designed variants with regard to MHET hydrolysis at 50 °C and pH 7.5 are presented in Table 1, Fig. 5a, and Table S13. The reaction temperature (50 °C) and pH value (7.5) for MHET hydrolysis were determined based on the optimal reaction conditions of the FAST-PETase with regard to PET depolymerization. Most of the in silico-designed variants (11/14) had improved catalytic efficiencies compared with the WT. Among the variants M8 (I171K/G130L, *k*_cat_/*K*_m_ = 4.70 mM^−1^·s^−1^), M13 (I171K/M127S/G130F, *k*_cat_/*K*_m_ = 8.11 mM^−1^·s^−1^), and M14 (I171K/M127S/G130L, *k*_cat_/*K*_m_ = 12.58 mM^−1^·s^−1^) achieved 36.0, 62.1, and 96.3 folds higher catalytic activities than the WT (*k*_cat_/*K*_m_ = 0.13 mM^−1^·s^−1^). The amino acid sequences of these variants are shown in Table S14. The increases in catalytic activity of M8 (*k*_cat_ = 21.43 s^−1^), M13 (*k*_cat_ = 20.99 s^−1^), and M14 (*k*_cat_ = 21.76 s^−1^) can be attributed predominantly to their rate enhancements relative to the WT (*k*_cat_ = 1.17 s^−1^). This is consistent with the calculations shown in Table 1, because our design strategy was to decrease the energy barriers via TS stabilization^73^. In addition, the nucleophilic attack distances of the three variants monitored in the 5 ns MD simulation exhibited a predominant conformational distribution at 2.6–3.6 Å, which was required for a catalytically productive state, whereas the nucleophilic attack distance of the WT was distributed over larger distances, and could not be maintained in the catalytically productive conformation (Fig. S10), indicating less efficient catalysis^49,62,74^. The protein melting temperature (*T*_m_) values of the WT and 14 variants were determined by differential scanning calorimetry (DSC), and are presented in Table 1, Fig. 5b, and Table S13. All the variants except for M1 (G130L, *T*_m_ = 76.39 °C) exhibited decreased thermal stability compared with the WT (*T*_m_ = 74.07 °C), but all the variants were well expressed, as confirmed by the sodium dodecyl sulfate–polyacrylamide gel electrophoresis results (Fig. S24). The mutations I171K and M127S, which were present in the highly efficient variants M8 (*T*_m_ = 67.58 °C), M13 (*T*_m_ = 63.29 °C), and M14 (*T*_m_ = 63.18 °C), introduced additional charged or polar functional groups to the hydrophobic cavity to form hydrogen bonds with the carboxyl group of MHET, as shown in Fig. 3d–f. This may explain the sacrifice of stability, though these mutations did contribute to the promotion of activity (Table 1) via TS stabilization. The introduction of charged or polar residues inside the active pocket generally disrupts the hydrophobic environment of protein^75^, which may explain the reduced thermal stability of the variants. The catalytic activities of variants M8, M13, and M14 decreased quickly when the hydrolytic temperature increased to 70 °C (Fig. S8). No secondary structure loss was observed at 50 °C for variant M8 (Fig. 5b). Besides, the heat inactivation experiments of variant M8 at 50 °C (Fig. S13) showed that M8 retained 75% residual activity after 96 h of incubation at 50 °C. All these results indicate that variant M8 is potentially compatible with FAST-PETase (*T*_m_ = 67.80 °C) at 50 °C for PET depolymerization. Furthermore, the temperature (Fig. S11) and pH activity (Fig. S12) profile of variant M8 confirmed that 50 °C and pH 7.5 are the optimal reaction conditions for M8 to catalyze the hydrolysis of MHET.

**Table 1:** Computational and experimental characterization of wild type Est30 and designed variants for MHET hydrolysis.

Variants	Mutations	ΔΔG_bind_^a^ (kcal/mol)	ΔΔG_fold_^b^ (kcal/mol)	Dist fraction^c^	*k*_cat_^d^ (s^−1^)	*K*_m_^d^ (mM)	*k*_cat_/*K*_m_^d^ (mM^−1^s^−1^)	Fold increase in *k*_cat_/*K*_m_^d^	*T*_m_^e^ (°C)
WT	——	——	——	11.66%	1.17 ± 0.14	8.98 ± 2.49	0.13	1.0	74.07 ± 0.14
M1	G130L	4.00	−14.31	15.45%	2.97 ± 0.21	5.36 ± 1.13	0.55	4.2	76.39 ± 0.36
M2	I171K	−5.55	4.85	24.87%	35.98 ± 18.20	53.70 ± 34.77	0.67	5.1	65.08 ± 0.33
M8	I171K/G130L	−0.08	−12.01	55.01%	21.43 ± 1.12	4.56 ± 0.75	4.70	36.0	67.58 ± 0.13
M13	I171K/G130F/M127S	−14.98	6.24	48.06%	20.99 ± 0.83	2.59 ± 0.41	8.11	62.1	63.29 ± 0.23
M14	I171K/G130L/M127S	−8.01	−3.43	34.45%	21.76 ± 0.62	1.73 ± 0.24	12.58	96.3	63.18 ± 0.20

^a^Difference of computed binding energy between variant and wild type.

^b^Difference of computed folding energy between variant and wild type.

^c^Fraction of the nucleophilic attack distance less than 3.0 Å observed during 5ns-MD simulation.

^d^Enzyme reactions were performed in triplicate over 10 min at 50 °C, using 0.5 μM purified enzyme in 50 mM sodium phosphate, pH 7.5.

^e^*T*_m_ was determined by differential scanning calorimetry from 30 to 120 °C and the assay conditions are 50 mM sodium phosphate, pH 7.5 and 0.5–1.0 mg·mL^−1^ enzyme. *T*_m_ values correspond to the average of two measurements.

The crystal structure of the most active variant M14 (I171K/M127S/G130L) was obtained to determine whether the computational design was realized in the actual experiments. The overall structure of M14 (PDB: 8ILT) and its alignment with that of the WT (PDB:1TQH) are shown in Fig. S14. The RMSDs of the main-chain atoms of M14 were only 1.51 Å, and the hydrolase domains of the two structures almost overlapped with each other. Compared with the WT, the two α-helices of the lid domain of M14 shifted further towards the hydrolase domain. This was consistent with the catalytic mechanism of a hydrolase, whereby the lid domain moves between the open and closed conformations depending on the substrate binding^63–65,68^. The alignment of the active sites of M14 and the WT in apo form are shown in Fig. 5c. The catalytic triad and oxyanion hole residues of M14 had similar conformations to those of the WT, with a RMSD of only 0.62 Å, implying that the rigid backbone assumption in our computational enzyme design method held. A recapitulation design was performed based on the crystal structure of M14, and the computed binding geometries (Fig. 5d) were aligned with that based on the crystal structure of the WT. The three introduced mutations I171K, M127S, and G130L in variant M14 were all in the lid domain, and their backbones were displaced from those of the WT. However, the computationally designed interactions, i.e., the hydrogen bonds between residues K171 or S127 and the carboxyl terminus of TS, and the hydrophobic stacking between L130 and the benzene ring of the TS all remained in the recapitulation to stabilize the TS in perfect catalytic binding geometry. This indicates that our computational enzyme design tool PRODA is robust enough to tolerate a certain degree of backbone fluctuation.

### Synergy between designed MHET hydrolase and PET hydrolase for PET depolymerization

**Fig. 6:**
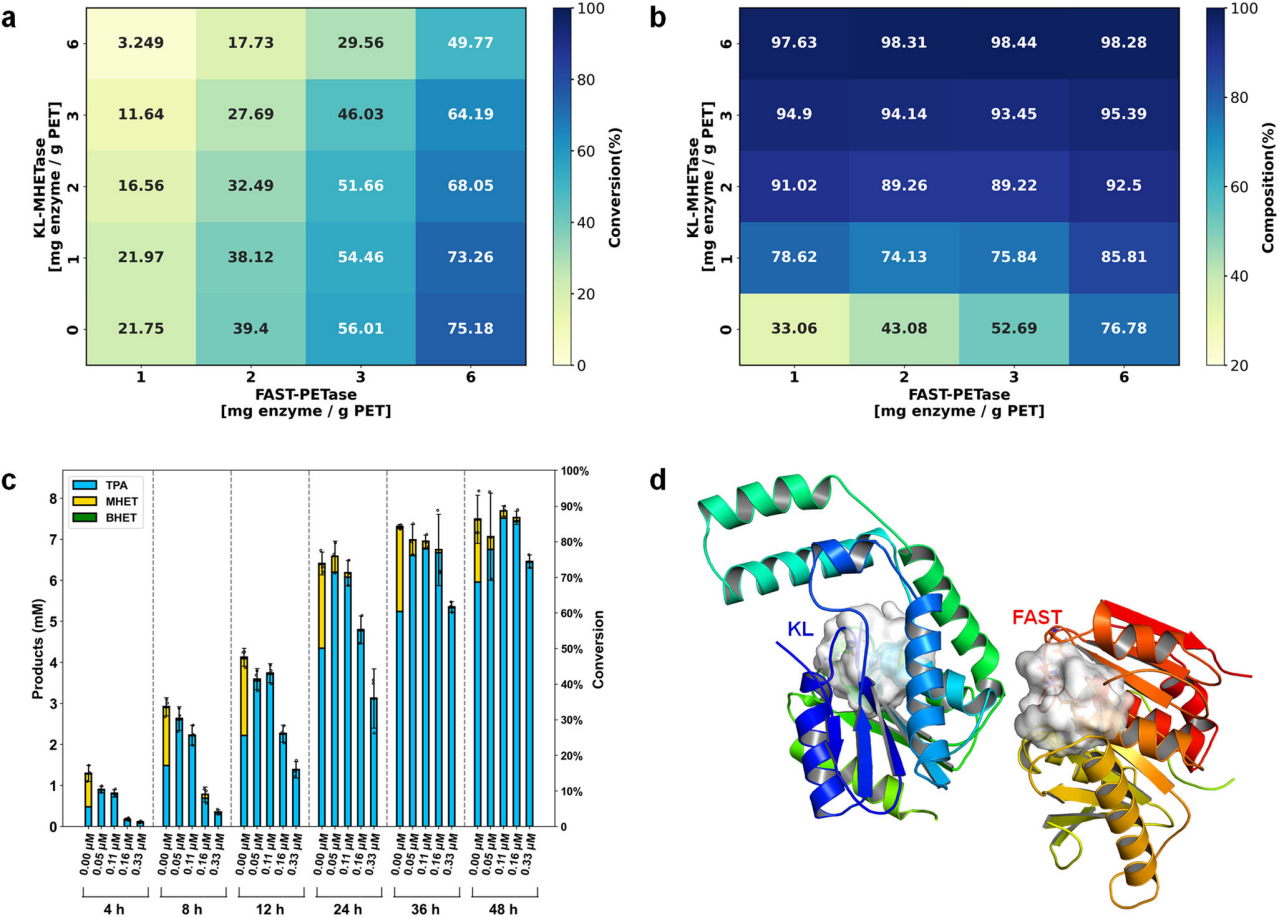
Synergy of FAST-PETase and KL-MHETase for PET degradation. **a** The conversion heatmap of synergistic PET degradation by FAST-PETase and KL-MHETase over 24 h at 50 °C. **b** The composition heatmap of synergistic PET degradation by FAST-PETase and KL-MHETase over 24 h at 50 °C. Abscissa: FAST-PETase loading (mg/g PET), Ordinate: KL-MHETase loading (mg/g PET). **c** Time course analysis of released PET degradation products, BHET (green), MHET (yellow) and TPA (blue) at 50 °C over 48 h with different KL-MHETase loadings. The concentration of FAST-PETase for all reactions was 0.35 μM (6 mg enzyme/g PET), concentrations of KL-MHETase were shown on abscissa. 0.33 μM of KL-MHETase corresponded to an enzyme loading of 6 mg enzyme/g PET. The PET conversion was shown on ordinate. Reactions were carried out in triplicate on post-consumer bottle grade PET powder (100–200 μm) at pH 8.0 in 100 mM sodium phosphate buffer. **d** Predicted complex structure of FAST-PETase and KL-MHETase by AlphaFold-Multimer^67^. The protein domains are shown as cartoon and colored. The active site residues are shown as white surface to indicate the substrate binding pocket.

Although the PET hydrolases (LCC-ICCG and FAST-PETase) had weak catalytic activities with regard to the hydrolysis of MHET (Table S13), their low catalytic efficiencies caused pronounced accumulation of MHET during PET depolymerization. The perceived inhibitory effect of MHET on the PET hydrolases led to inefficient PET depolymerization and a low TPA yield (Fig. S15). To overcome the aforementioned obstacles to PET depolymerization using a single enzyme, the synergistic effect of a mixed two-enzyme system was investigated by measuring the extent of hydrolysis of a post-consumer bottle grade PET powder substrate (Pc-PET, particle size 100–200 μm, crystallinity 2.9%, Table S17) over 24 h at 50 °C using FAST-PETase and the designed MHET hydrolase M8 (here denoted as KL-MHETase) at various concentrations (Fig. 6a, b). Over the range of enzyme loadings tested (1–6 mg FAST-PETase/g PET, 0–6 mg KL-MHETase/g PET), the conversion of PET, as determined by concentration of aromatic product released (the sum of BHET, MHET, and TPA), scaled with FAST-PETase loading (Fig. 6a), and the composition of TPA, as determined by concentration of TPA in total products, scaled with KL-MHETase loading (Fig. 6b). The conversion of PET decreased as KL-MHETase loading increased at any particular FAST-PETase loading, indicating that the addition of KL-MHETase affected the binding between the PET polymer substrate and FAST-PETase. The complex structure of the dual enzyme system was predicted using the protein structure prediction tool AlphaFold-Mutimer^67^, as shown in Fig. 6d. The substrate-binding cleft of FAST-PETase was partially blocked by the hydrolase domain of KL-MHETase. Owing to the interaction between KL-MHETase and FASTase, the composition of TPA varied nonlinearly with the increase in FAST-PETase loading at any particular KL-MHETase loading (Fig. 6b), indicating that there was an optimal ratio of KL-MHETase to FAST-PETase for maximum TPA yield. To find the optimal ratio, the time-course of PET depolymerization (Fig. 6c) was measured over the range of KL-MHETase loadings (0–0.33 μM enzyme/g PET) with fixed FAST-PETase loading (0.35 μM enzyme/g PET). The synergy study showed that both the conversion of PET and the yield of TPA over 48 h were at their maximum values when the molar ratio of KL-MHETase (0.11 μM enzyme/g PET) to FAST-PETase (0.35 μM enzyme/g PET) was 2:6, and were higher than the corresponding values obtained using FAST-PETase alone. This optimal value was further corroborated by the synergistic PET degradation by FAST-PETase and KL-MHETase at various ratios over 48 h at 50 °C (Fig. S16a, b). It should be noted that during the first 36 h, the conversion of PET using mixed dual enzyme systems with various ratios of KL-MHETase to FAST-PETase fell behind that using the single enzyme FAST-PETase owing to the deleterious interaction between the two enzymes (Fig. 6d). This defect should be removed to exploit the cascade effect of the dual enzyme system for fast PET degradation.

### Engineering of fusion dual enzyme system for fast PET depolymerization

**Fig. 7:**
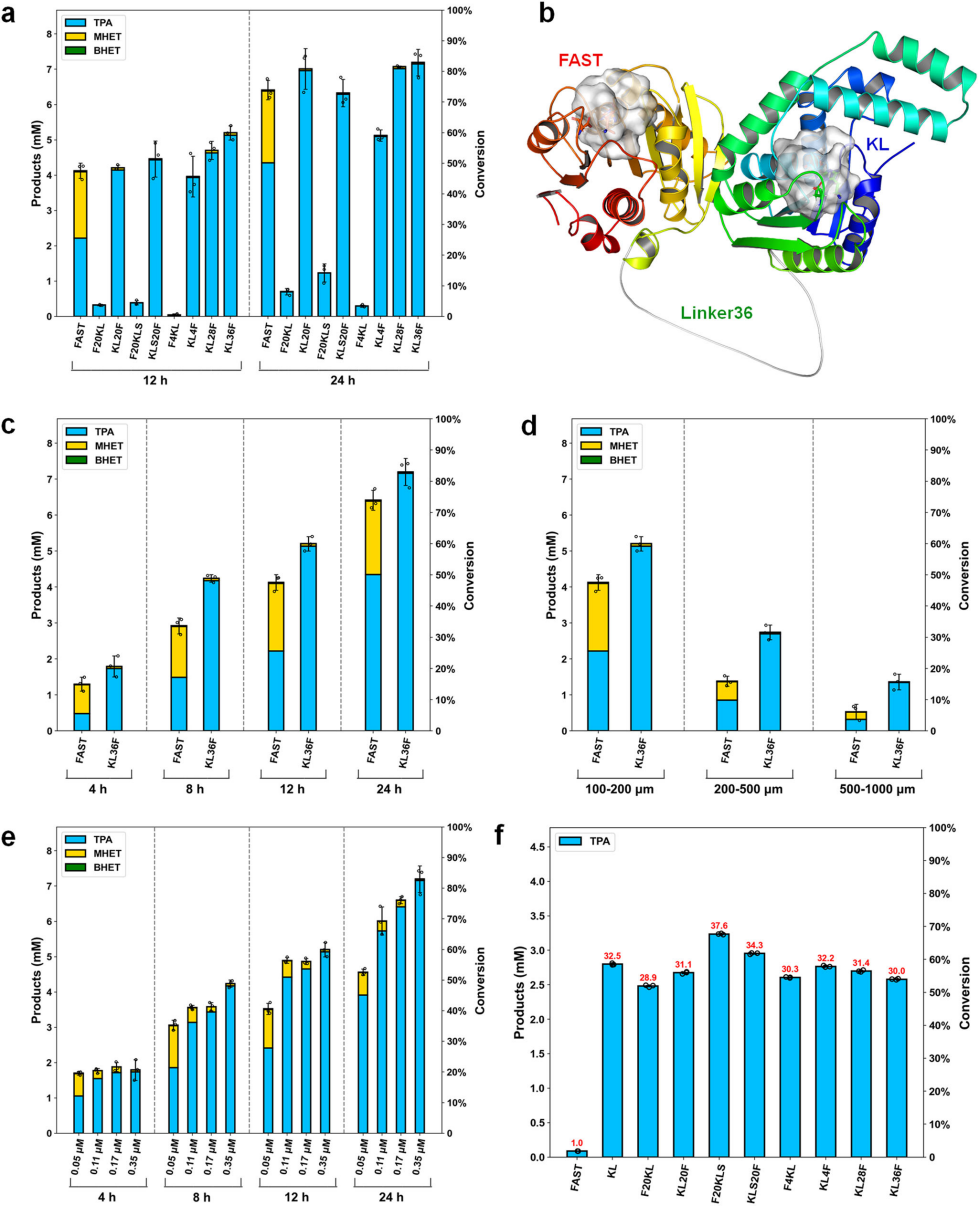
Fused dual enzyme systems of FAST-PETase and KL-MHETase for PET degradation. **a** PET degradation by FAST-PETase and eight fused dual enzyme systems on post-consumer bottle grade PET powder (Pc-PET, 100–200 μm) over 12 h and 24 h, respectively, each using 0.35 μM enzyme loading. **b** Predicted structure of the fusion enzyme KL36F by AlphaFold2^66^. The protein domains are shown as colored cartoon while the linker colored in white. The active site residues for both enzymes are shown as white surface to indicate the substrate binding pockets. **c** Time-course analysis of PET degradation by FAST-PETase and KL36F on Pc-PET powders (100–200 μm) over 24 h, each using 0.35 μM enzyme loading. **d** PET degradation conversion and product composition by FAST-PETase and KL36F on Pc-PET powders with different particle sizes (100–200 μm, 200–500 μm, 500–1000 μm) over 12 h, each using 0.35 μM enzyme loading. **e** Effect of different enzyme loadings (0.05 μM, 0.11 μM, 0.17 μM, 0.35 μM) on PET degradation by KL36F on Pc-PET powders (100–200 μm) over 24 h. **f** Relative activities of FAST-PETase, KL-MHETase and eight fusion enzymes towards MHET hydrolysis. The reactions were performed over 10 min at 50 °C using 4.8 mM MHET and 0.5 μM enzyme loading in 50 mM sodium phosphate, pH 7.5. The activity increase folds of fusion enzymes relative to FAST-PETase are labeled in red numbers. BHET (green), MHET (yellow) and TPA (blue). All PET degradation reactions were performed in pH 8.0, 100 mM sodium phosphate using 0.16% Pc-PET substrate loading (1.66 g·L^−1^) at 50 °C. The reactions were carried out in triplicate. The PET conversion was shown on right ordinate.

It is possible to control the complex structure of a dual enzyme system by introducing a peptide linker between the enzymes^23^. We hypothesized that it would be possible to fully expose the active site of FAST-PETase, which binds the PET polymer substrate, by constructing an appropriate linker between the PET hydrolase and the in silico-designed MHET hydrolases. Herein, the most active variant M14 (denoted as KLS-MHETase) was also invoked, although its *T*_m_ (63.18 °C) is too low for a reaction at 50 °C compared to the *T*_m_ of FAST-PETase. A series of flexible glycine and serine linkers (L20, L28, and L36) of various lengths and one rigid linker (L4) whose amino acid sequences are given in Table S16 were used to covalently link the C terminus of KL-MHETase or KLS-MHETase to the N terminus of FAST-PETase or vice versa, resulting in the construction of a total of eight fused enzymes (Table S16). The PET degradation activities (Fig. 7a) of those eight fusion dual enzyme systems used at the same molecular concentrations were investigated at 50 °C using a Pc-PET powder substrate (particle size 100–200 μm) over 12 or 24 h. The PET degradation activities of the fusion dual enzyme systems were strongly dependent on the linking order between PETase and MHETase, the amino acid sequences of the linkers, and the lengths of the linkers. The three fusion dual enzyme systems KL20F, KL28F, and KL36F demonstrated higher conversion rates than the single enzyme FAST-PETase over 24 h. The complex structures of the eight dual enzyme systems were predicted using the protein structure prediction tool AlphaFold2^66^, as shown in Fig. 7b and S17. As expected, the substrate-binding clefts of the FAST-PETases in KL20F, KL28F, and KL36F were completely exposed, and the active sites of the KL-MHETases in those fusion enzymes were orientated towards the active sites of the FAST-PETases. Furthermore, the active site orientation of two enzymes in KL36F was validated by long time MD simulations (Fig. S18). This facilitated the direct diffusion of the PET degradation intermediate product MHET into the active site of KL-MHETase. The intermediate product MHET was degraded into the constituent monomers TPA and EG in situ, and the inhibitory effect of MHET on FAST-PETase was immediately eliminated, which promoted enzyme synergy and improved catalytic efficiency. It should be noted that the PET degradation rate of the fusion dual enzyme system KLS20F slowed down over 24 h, although it was greater than the activity of the fusion dual enzyme system KL20F over 12 h, probably because KLS20F afforded higher catalytic efficiency (Table S13) with regard to MHET hydrolysis but worse thermal stability than KL20F (Fig. S19, Table S13).

The enzyme cascade effect of the constructed dual enzyme system was investigated by time-course comparative analysis of PET degradation using the most active fusion enzyme KL36F and the single enzyme FAST-PETase (Fig. 7c). Under the same enzyme loadings (molar concentrations), the PET degradation intermediate product MHET hardly accumulated over 24 h using the fusion enzyme KL36F, whereas using the single enzyme FAST-PETase the composition of MHET in the sum of released products was always above 31.7% (Fig. 7c). As a result, the degradation conversion of PET, the yield of TPA, and the composition of TPA in the released products using fusion enzyme KL36F reached 82.9%, 82.5%, and 99.5% after 24 h, respectively, which were 1.12, 1.64, and 1.47 folds greater than the corresponding values using FAST-PETase (74.0%, 50.2%, and 67.8%, respectively). As the particle size of the Pc-PET powder increased, the degradation rate of PET accelerated more with the fusion enzyme, although the crystallinity of the substrate increased (Table S17). When the Pc-PET particle sizes were 200–500 μm and 500–1000 μm, the degradation conversion rates of PET after 12 h using the fusion enzyme KL36F were 2.0 and 2.6 folds higher than those using FAST-PETase, and the TPA yields were 3.1 and 4.2 folds higher, respectively (Fig. 7d). The fusion enzyme KL36F was further compared with FAST-PETase across a range of temperatures (Fig. S20, 40–60 °C) and pH values (Fig. S21, 6.0–9.0). The results suggest that KL36F emerged as a superior candidate for PET degradation because it demonstrated higher activity and thermal stability across the tested conditions.

The purity of TPA in the released products was dependent on the fusion enzyme loadings. The composition of MHET was less than 0.5% after 24 h when the fusion enzyme KL36F loading was 0.35 μM, whereas it increased to 13.9% when the enzyme loading decreased to 0.05 μM (Fig. 7e). The catalytic efficiency of KL36F with regard to MHET hydrolysis was 3.64 mM^−1^·s^−1^ (Table S13), which was comparable to that of KL-MHETase and 52.6-fold higher than that of FAST-PETase. Although the hydrolytic activities of the fusion enzymes with regard to MHET were approximately 28–37-fold higher than the hydrolytic activity of FAST-PETase (Fig. 7f), the dominant degradation pathway of FAST-PETase on PET polymer is random in-chain scission. This is because the PET hydrolase FAST-PETase has a surface-exposed, shallow active site^16^, which results in an exponential rate of production of the degradation intermediate product MHET, leading to the accumulation of MHET when the fusion enzyme loadings are low. This implies that a thermostable MHET hydrolase with even higher catalytic activity is critical for the development of more economical PET recycling processes in which the enzyme loading is reduced.

**Fig. 8:**
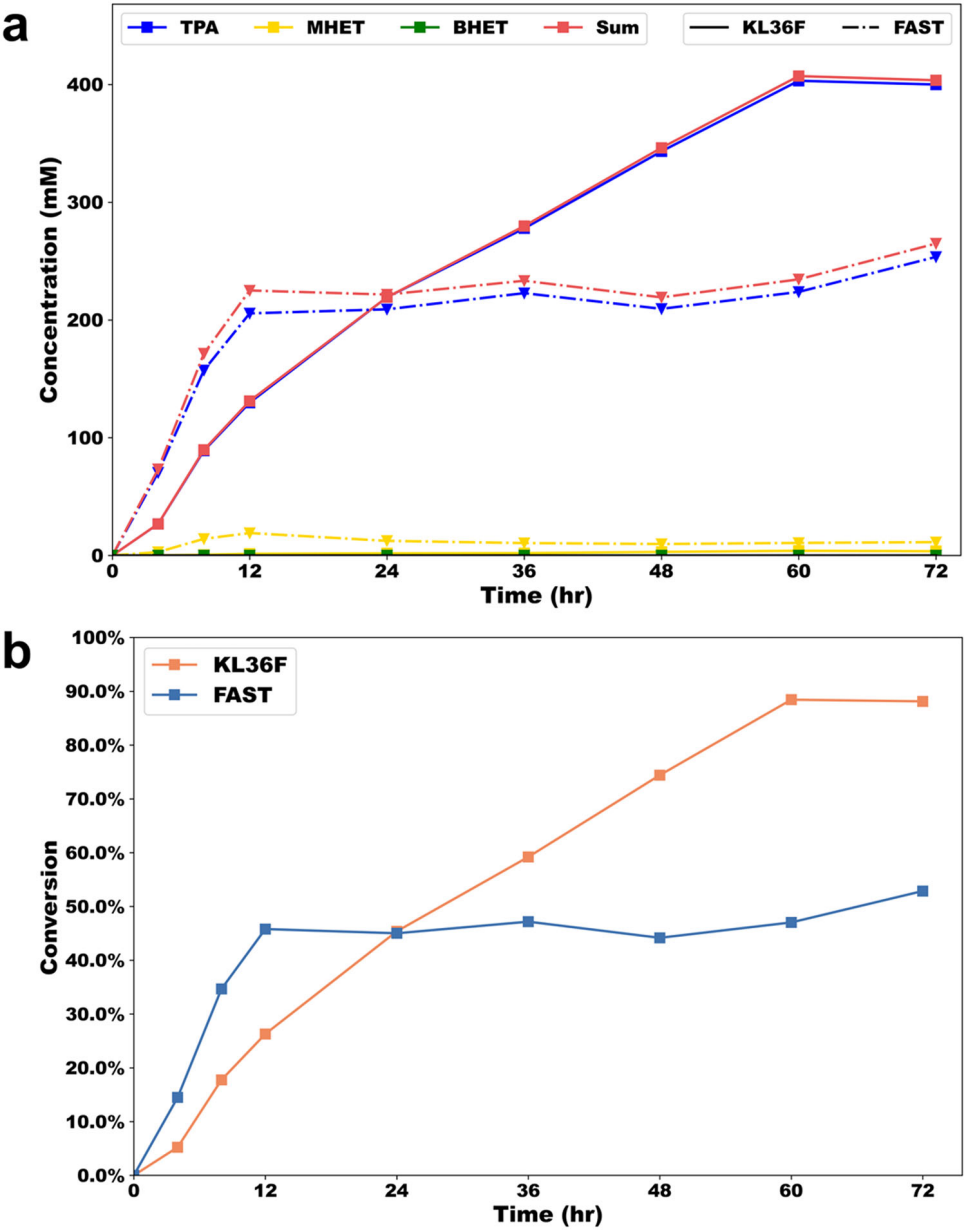
Time course of PET degradation over 72 h for FAST-PETase and KL36F under high solid loadings in bioreactor (50 mL). **a** Time course of aromatic products released. **b** Time course of degradation conversion. Reactions were performed in 100 mM sodium phosphate using 10% Pc-PET (100–200 μm) substrate loading (100 g·L^−1^) and 12 mg FAST/g PET (40.5 μM) or 24 mg KL36F/g PET (40.5 μM) at 50 °C with continuous pH control around 8.0.

We further compared the performance of FAST-PETase and KL36F in large reaction systems (PET substrate concentration of 100 g/L and system volume of 50 mL). As that shown in Fig. 8, KL36F achieved PET conversion and TPA yield of 90% and 89.0%, respectively, at 60 h, which were 1.9 and 1.8 times higher than those of FAST-PETase. Notably, the purity of TPA in the aromatic products obtained in the dual enzyme system exceeded 99% at any time point and the MHET concentration of the system was only up to 4 mM. In contrast, the MHET concentration in the single enzyme system reached up to 20 mM, which may have led to a product inhibition of FAST-PETase, resulting in a stagnant conversion of 53% for PET degradation. Another replicate experiment showed similar results, as that shown in Fig. S22. These results suggest that the fusion enzyme KL36F retains its superior performance over FAST-PETase under high substrate concentrations^76^, thereby demonstrating its potential applicability in the industrial recycling of PET.

## Discussion

Although MHET hydrolases are crucial for PET depolymerization into its constituent monomers, the currently available MHET hydrolases, which are either thermally unstable or have poor activity, cannot match the recently designed PET hydrolases such as FAST-PETase^12^ and LCC-ICCG^11^ at their optimal reaction temperatures (50 or 70 °C). Simultaneously promoting stability and activity is a great challenge for enzyme engineering because the evolution of one is always accompanied by loss of the other^77,78^. Our design strategy was to first search a structural database for a thermophilic scaffold with promiscuous activity with regard to MHET hydrolysis, and then promote its catalytic activity by computational active site redesign. Thanks to the sub-angstrom accuracy and speed of our active site-matching algorithm ProdaMatch, we were able to identify five thermostable scaffolds from the experimental structural database (PDB), which comprised 0.18 million protein entries, and we experimentally confirmed their promiscuous activities with regard to the hydrolysis of MHET at 50 °C. As shown in Fig. S23, the most efficient PET hydrolase (LCC-ICCG) was able to degrade nearly 100% of the PET powder at 70 °C within only 24 h, but MHET still accounted for a large portion (24%) of the aromatic product. Therefore, we anticipate that our active site-matching ProdaMatch algorithm will be used to search more thermostable scaffolds that are able to match LCC-ICCG at 70 °C from the predicted structural database (AlphaFold), which has over 200 million protein entries with acceptable accuracies for scaffold selection.

Although a selected scaffold may be thermostable, active site redesign to promote its catalytic activity may compromise its stability^77^. Therefore, the computational enzyme design algorithm should be able to select mutations that promote the catalytic activity of the scaffold without adversely affecting its melting temperature. The structural characteristics of the substrate MHET suggest that one Lys or Arg should be introduced into the active site of the selected scaffold to build a strong hydrogen bonding network with the terminal carboxyl group of MHET to stabilize the TS. But, the introduction of one positively charged Lys or Arg in the hydrophobic pocket of a protein tends to compromise its stability^75^. In fact, one Lys was introduced in all the highly active variants based on the 1TQH scaffold, i.e., M8 (I171K/G130L), M13 (I171K/M127S/ G130F), and M14 (I171K/M127S/G130L), and we noted that the single mutation I171K in variant M2 (*T*_m_ = 65.08 °C) decreased its melting temperature by 9 °C compared with that of the WT (*T*_m_ = 74.07 °C). Our computational enzyme design algorithm PRODA identified mutations G130L and G130F, and we found that the single variants M1 (G130L) and M3 (G130F) (Table S13) had improved catalytic efficiencies and higher melting temperatures. Moreover, we noted that the mutation G130L in variant M1 promoted the turnover number compared with that of the WT owing to the hydrophobic stacking between L130 and the benzene ring of the TS. The combination of mutations I171K and G130L produced the highly efficient thermostable variant M8, whose rate constant (21.43 s^−1^) at 50 °C is comparable to that of the native MHETase (11.1/31/50.58 s^−1^)^22,23,64^ at 30 °C. The protein melting temperature of M8 (*T*_m_ = 67.58 °C) is comparable to that of FAST-PETase (*T*_m_ = 67.80 °C). Moreover, the catalytic efficiency (4.70 mM^−1^s^−1^, Table S13) of M8 with regard to MHET hydrolysis is 67.14-fold higher than that (0.07 mM^−1^s^−1^, Table S13) of FAST-PETase. Therefore, we identified a MHET hydrolase that could match PET hydrolase FAST-PETase in terms of PET depolymerization activity at 50 °C. All the MHET hydrolases are reported in Table S1. The MHET hydrolases that could operate properly over 50 °C, e.g., TfCa-WA (I69W/V376A) and TTCE, all had modest activities with regard to MHET hydrolysis, i.e., hundreds of times less active than M8 (KL-MHETase). The Est30 scaffold has a smaller lid structural domain compared to the extensive lid structural domain of natural MHETase (Fig. S6), which enables the enzyme binding pocket to capture intermediates (mainly MHET) more readily during PET degradation by PET hydrolases in cascade catalysis. The homologous sequence alignment between the Est30 scaffold and the native MHETase^23^ is shown in Fig. S7, and the low sequence identity of the two scaffolds (17.4%) demonstrates the potential of our computational enzyme design method for inventing new enzymes.

The polymeric characteristics of the PET substrate and protein–protein interactions demand extraordinary control of the orientation of the active sites of MHET hydrolase and PET hydrolase. Moreover, the complex structure spontaneously formed between KL-MHETase and FAST-PETase in a mixed dual enzyme system has restricted PET degradation efficiency compared with that of a single enzyme. With the aid of protein structure prediction tools AlphaFold2^66^ and AlphaFold-Mutimer^67^ we constructed suitable peptides to link KL-MHETase and FAST-PETase. The engineered fused dual enzyme system KL36F depolymerized 82.5% of a Pc-PET powder into its constituent monomer TPA within 24 h, and TPA accounted for more than 99.5% of the released aromatic products. In contrast, the TPA yield and purity were only 50.2% and 67.8%, respectively, for the single enzyme FAST-PETase. In large reaction systems with 100 g/L substrate concentrations, the dual enzyme system KL36F achieved over 90% PET depolymerization into monomers, which was far greater than that (55%) of single enzyme system FAST-PETase. These results suggest that the fusion enzyme KL36F has achieved industrial degradation of PET at 50 °C, and the solid loading capacity reaches the industrial scale demonstrated by LCC-ICCG at 72 °C. The dual enzyme synergistic effect was not detected in the recently reported dual enzyme systems TfCut2-TfCa^37^, immobilized PETase-MHETase^23^, LCC-TTCE^39^, and PETase PM-TfCa WA^40^ because either their MHET hydrolases were not stable at 50 °C or their catalytic activities were too low.

The computational enzyme design strategy developed in the present research is general, and we expect that super-thermostable MHET hydrolases that are stable at the optimal PET degradation temperature (70 °C) will be developed soon. The extremely pure TPA from the conversion of post-consumer PET plastics by dual enzyme systems may be directly used for polymer synthesis after applying simple separation techniques such as centrifugation and crystallization. This will greatly reduce the cost of recycling PET. Therefore, the present study will help to expedite the industrialization of the enzymatic recycling of PET plastics, and will ultimately contribute to a circular economy.

## Methods

### Structural data

In total, 104 protein structures used for scaffold selection and computational enzyme design were obtained from the PDB^79^. The crystal structure of the designed enzyme KLS-MHETase was determined in the present study and deposited in the PDB (PDB Code: 8ILT). Water molecules in the original 1TQH scaffold for active site redesign were removed and the amino acid hydrogen atoms were added using PRODA based on the topology parameters of the all-atom CHARMM 22 force field^80^. The atomic coordinate of substrate MHET was taken from PubChem (https://pubchem.ncbi.nlm.nih.gov), and hydrogen atoms were added using the molecular modeling software Discovery Studio. The protonation state of the residues of the 1TQH scaffold was assigned after carefully examining the environment around each residue and referring to the pKa values given by PROPKA 3.0^81^ at the reference pH (7.5).

### Scaffold selection

A thermophilic hydrolase scaffold library with 104 protein entities was collected from the PDB based on the following criteria: (i) it was possible to express the protein in *Escherichia coli*; (ii) it was possible to obtain the protein structure at a structural resolution of better than 3.0 Å by X-ray crystallography; (iii) the length of the amino acid sequence was between 200 and 800; (iv) the sequence identity in the set was lower than 95%; (v) the results were determined by searching the keywords: “thermophilic, thermophile, thermophila, thermophilus”; and (vi) the enzyme classification name was hydrolases. For each scaffold, the three largest pockets were identified using the cavity-detecting module ProdaPocket in PRODA, with each pocket including up to 100 design positions. The scaffold-independent parameter used to evaluate the geometry penalty was obtained and is shown in Table S4. The fast and accurate active site matching algorithm ProdaMatch^57,58^ in PRODA was used to check whether the active site model could be anchored into the incumbent scaffold. All matches produced in one scaffold were ranked by a scoring function. To identify scaffolds with a catalytic triad motif, catalytic residues Ser and His were anchored at positions with identical amino acid types, whereas Asp was anchored at positions whose native types were either Asp or Glu.

### Computational enzyme design

The computational active site redesign of Est30 was carried out using the PRODA enzyme design protocol^54,56–58^. The amino acid hydrogen atoms were added using PRODA in accordance with the topology parameters of the all-atom CHARMM 22 force field. The sequence selection positions that are allowed variation of the amino acid type, and the residues that provided optimal side-chain conformations are shown in Table S7. The backbone atoms at the selected positions, and all atoms at other positions, were kept rigid and referenced as the scaffold template. A backbone-independent rotamer library^82^ containing 11,810 original rotamers was used to model the side-chain conformations of the design sites. In addition, the crystal conformations at the design positions of the scaffold template were included in the rotamer library. The rotamers of serine, threonine, and tyrosine were expanded owing to the diverse configurations adopted^57^ by the hydroxyl hydrogen atom. The substrate structure of MHET (Fig. S3) in the TS was built by changing the central ester atom so that it had an intermediate tetrahedron geometry. The atomic van der Waals parameters for MHET were obtained from the model molecules of the CHARMM 22 force field, and the atomic partial charges were assigned based on the PARSE models^83^. A library of TS conformers for the computational model was generated and screened in accordance with the placing rules (Table S5) and catalytic geometrical constraints (Table S6) using the previously proposed small-molecule placement approach^55,72^. The free energy of the enzyme–substrate complex in the TS was calculated using the energy functions mentioned in our earlier work^55–57^. The mutation sequences corresponding to the global or near-global minimum energy conformations (GMEC) of the enzyme–substrate complex in the TS were computed using the dead-end elimination/linear programming/mixed-integer linear programming-based deterministic global optimization algorithm developed in our previous work^55–57^. To adequately search the conformational and sequence space, a large number of sequences around the global minimum energy sequence were generated by imposing the restriction whereby a predefined number of rotamer types were different for any two sequences. The free energy of the bound enzyme-TS complex system (∆*G*_bound_) was the sum of binding energy (∆*G*_bind_) and folding energy (∆*G*_fold_). The binding energy (∆*G*_bind_) was calculated as the energy difference between the bound enzyme-TS system and the unbound enzyme-TS system, as shown in Eq. 1:

1 $$\Delta {G}_{{{{{{\rm{bind}}}}}}}=\Delta {G}_{{{{{{\rm{bound}}}}}}}-\Delta {G}_{{{{{{\rm{unbound}}}}}}}$$

where ∆*G*_bound_ and ∆*G*_unbound_ are the free energies of the complex and the unbound enzyme-TS systems.

The folding energies are the free energies of the apo-form enzyme, equal to ∆*G*_unbound_, as shown in Eq. 2:

2 $$\Delta {G}_{{{{{{\rm{fold}}}}}}}=\Delta {G}_{{{{{{\rm{unbound}}}}}}}$$

The binding energy change upon mutation ($\Delta \Delta {G}_{{{{{{\rm{bind}}}}}},{{{{{\rm{Mut}}}}}}}$) is computed as the difference of the binding energies of the wild-type ($\Delta {G}_{{{{{{\rm{bind}}}}}},{{{{{\rm{WT}}}}}}}$) and mutant enzyme-TS system ($\Delta {G}_{{{{{{\rm{bind}}}}}},{{{{{\rm{Mut}}}}}}}$), as shown in Eq. 3:

3 $$\Delta \Delta {G}_{{{{{{\rm{bind}}}}}},{{{{{\rm{Mut}}}}}}}=\Delta {G}_{{{{{{\rm{bind}}}}}},{{{{{\rm{Mut}}}}}}}-\Delta {G}_{{{{{{\rm{bind}}}}}},{{{{{\rm{WT}}}}}}}$$

The folding energy charge upon mutation ($\Delta \Delta {G}_{{{{{{\rm{fold}}}}}},{{{{{\rm{Mut}}}}}}}$) is computed as the difference of the folding energies of the wild-type ($\Delta {G}_{{{{{{\rm{fold}}}}}},{{{{{\rm{WT}}}}}}}$) and mutant enzymes ($\Delta {G}_{{{{{{\rm{fold}}}}}},{{{{{\rm{Mut}}}}}}}$), as shown in Eq. 4:

4 $$\Delta \Delta {G}_{{{{{{\rm{fold}}}}}},{{{{{\rm{Mut}}}}}}}=\Delta {G}_{{{{{{\rm{fold}}}}}},{{{{{\rm{Mut}}}}}}}-\Delta {G}_{{{{{{\rm{fold}}}}}},{{{{{\rm{WT}}}}}}}$$

More details can be found in the SI.

### Molecular dynamics simulation

The MD simulations were carried out using GROMACS 2019.4^84^. The initial complex coordinates of the designs were copied from the results computed by PRODA. The topologies of the designs were generated by GROMACS using the CHARMM36 all-atom force field. The topology of the corresponding small molecule, i.e., the TS, was prepared using the online CGenFF program 1.0.0^85,86^ and its atom types, bond parameters, and atomic partial charges were built to be consistent with the definitions of the CHARMM General Force Field 3.0.1^87,88^. The δ nitrogen of the catalytic histidine residue (His223) was protonated in accordance with the catalytic mechanism of an esterase. The new protein–TS complex structure was immersed in a dodecahedral box whose size was determined by setting the distance between the solute and the box to 10 Å, and the box was filled by the addition of approximately 12,000 explicit water molecules represented by the water model TIP3P^89^. The charge of the system was neutralized by adding an appropriate number of Na^+^ or Cl^−^ counter ions to the solvent box.

Each simulation was independently initiated with energy minimization (EM) of the system using the steepest descent minimization algorithm, and EM continued until the potential energy was negative and the maximum force F_max_ was not greater than 1000 kJ∙mol^−1^∙nm^−1^. Next, a position-restrained NVT phase (NVT = constant temperature and constant volume) simulation was performed using simulated annealing to warm the system. For the 1 and 5 ns MD simulations, the temperature was gradually increased from 0 to 343 K within 150 ps, then stabilized at 343 K for a further 30 ps. Subsequently, the system underwent a position-restrained NPT (NPT = constant temperature and constant pressure) simulation of 100 or 200 ps, and 1 or 5 ns production MD simulation, respectively. The time step was set to 1 fs for NVT and 2 fs for NPT. Constant temperature was maintained using the velocity rescaling thermostat^90^ and constant pressure was maintained using the Berendsen barostat^91,92^. In the position-restrained simulations and MD simulations, the LINCS algorithm was used to impose constraints on the bonds and angles of the complex. The Particle Mesh Ewald method^93,94^ was used to model long-range electrostatic effects, and short-range van der Waals interactions were cut off at 12 Å. The differential equations of motion were integrated by a leap-frog algorithm. The 1 and 5 ns MD simulations were repeated 10 and 5 times, respectively; the time step was 2 fs, and the coordinates of all the atoms in the system were saved every 10 ps, which generated a total of 100 frames and 500 frames, respectively. Post-simulation data extraction and analysis were performed using the GROMACS analysis tools. Snapshots of enzyme interactions with MHET were analyzed and visualized using PyMOL 2.4.0 software (https://pymol.org/). More details can be found in the SI.

### Protein structure prediction

The prediction tool AlphaFold2^66^ was used to predict the protein structure of each fusion protein. AlphaFold-Multimer^67^ was used to predict the structure of the KL-MHETase and FAST-PETase protein complexe. The number of models was set at five. The “max_template_data” was set at “2022-10-10”. The “preset” parameter was set at “full_dbs” to balance the time of prediction and the quality of the result. The result model with the highest pLDDT score was selected for further visual inspection and analysis.

### Chemicals

Bis(2-hydroxyethyl) terephthalate (BHET), mono (hydroxyethyl) terephthalate (MHET), and terephthalic acid (TPA) were obtained from Bide Pharmatech Ltd (Shanghai, China).

### Gene cloning, plasmid construction, and site-directed mutagenesis

The genes of selected scaffolds were obtained from Genebank (https://www.ncbi.nlm.nih.gov /genbank/), and the codon was optimized using a web tool (http://genomes.urv.es/OPTIMIZER/). All genes were synthesized by the Beijing Genomics Institution (Beijing, China). The pET-22b (+) vector was combined with each synthesized gene to create an ampicillin-resistant clone. The synthesized gene fragment was ligated into the *NdeI* and *XhoI* sites on the vector, and the C-terminal hexa-histidine tag was ligated into the WT enzyme and its mutants. Variants of Est30 were created by site-directed mutagenesis and the mutations were validated by automated DNA sequencing techniques.

### Enzyme expression and purification

The *Escherichia coli* strain BL21(DE3) provided the host cells for protein cloning and expression of the WT and mutant enzymes, and the strains were grown overnight (over 14 h) at 37 °C in solid lysogeny broth (LB) medium containing 75 μg·mL^−1^ ampicillin. Single colonies growing well were transferred into 50 mL of liquid LB medium with 75 μg·mL^−1^ ampicillin, and incubated at 37 °C and 200 rpm for 7 h. Next, 2 mL of each liquid bacterial germ was transferred to 200 mL of fresh liquid LB medium with 75 μg·mL^−1^ ampicillin, and incubated at 37 °C and 200 rpm until the OD600 (the optical density of a sample measured at a wavelength of 600 nm) reached 0.6–0.8. Protein expression was induced with 0.5 mM isopropyl-β-thiogalactopyranoside, and cells were harvested after a further incubation at 18 °C and 200 rpm for over 16 h. After centrifugation, the cell pellet was resuspended in 50 mM sodium phosphate buffer (pH 7.5) and subjected to 30 min of ultrasonication cycles (4 s on and 6 s off) at 60% amplitude. The periplasmic extracts were centrifuged at 10,000 *g* for 30 min, and the insoluble cell debris was removed. The clarified lysate was then purified in a 5 mL HisTrap HP (Cytiva) column using an ӒKTA Pure chromatography system (Cytiva) according to the manufacturer’s instructions. Subsequently, the fractions containing the purified enzyme were desalted three times using a 10 kDa cut-off Amicon® Ultra 15 mL Centrifugal Filter device (EMD Millipore Corporation). The protein concentration was then determined using a BCA^95^ Protein Assay Kit (Beyotime Biotechnology) and an Infinite M200 PRO microplate reader (Tecan) to measure the absorbance (A562 nm) of each assay mixture. The presence and purity of the purified proteins were assessed by sodium dodecyl sulfate–polyacrylamide gel electrophoresis (Fig. S24).

### PET powder preparation

Post-consumer bottle grade PET was obtained from commercial bottles (body only; the neck and base were removed from the bottle) from the Coca-Cola Company (China). The PET was cut into a 10 × 10 mm pieces, washed with water, 0.5% sodium dodecyl sulfate, and ethyl alcohol, dried, and shredded into particles using an AM500S high-speed rotary mill (Ants Scientific Instruments (Beijing) Co., Ltd) operated at 8000 rpm. The particles were sifted using 100, 200, 500, and 1000 μm sieves to obtain post-consumer bottle grade PET powders with particle sizes in the ranges 100–200, 200–500, and 500–1000 μm.

### PET depolymerization assay

Different particle sizes in the ranges 100–200, 200–500, and 500–1000 μm of post-consumer bottle grade PET powders were used for depolymerization. An enzyme solution (3 mL) in 100 mM sodium phosphate (pH 8.0) was incubated with appropriately 5.0 mg of the PET substrate in a 5 mL glass bottle. The concentration of FAST-PETase was 0.35 μM (6 mg enzyme/g PET) in the single enzyme experimental group, while the concentration of KL-MHETase was 0.05, 0.11, 0.16, 0.33 μM in the dual enzyme experimental group. The concentration of all fusion enzymes used was 0.35 μM, while the concentration of KL36F was 0.05, 0.11, 0.17, 0.35 μM in the experimental group for testing effect of enzyme loadings. The test bottle was tightly capped and wrapped in parafilm to minimize volatilization. The bottle was then incubated in a heat block (Jingxin, Shanghai) at 50 or 70 °C and agitated at 800 rpm for the indicated time (4, 8, 12, 24, 36 or 48 h). At each specified time-point, the reaction was terminated by adding an equal volume of methanol and the supernatant was collected by centrifugation (10,000 g for 3 min). Prior to analysis, each sample was diluted 10-fold, and filtered through a 0.25 μm filter plate using a vacuum syringe into a 2 mL collection plate, which was sealed with a silicone mat. The amounts of the products of PET hydrolysis, i.e., BHET, MHET, and TPA, were quantified by high-performance liquid chromatography (HPLC). All samples were analyzed in triplicate in each independent experiment, and the average value and standard deviation were calculated.

### PET depolymerization bioreactor assay

The large scale reaction was carried out in 50 mL phosphate buffer (100 mM, pH 8.0) in duplicate in a three-necked flask. 5 g (10%) of 100–200 μm post-consumer PET powder was added. The amount of FAST-PETase added was 60.0 mg, resulting in a final concentration of 12 mg/g PET (40.5 μM), while the amount of KL36F added was 120.0 mg, resulting in a final concentration of 24 mg/g PET (40.5 μM). The three-necked flask was placed in a heat-collecting constant temperature magnetic stirrer to stir the reaction solution and keep the reaction temperature at 50 °C. Meanwhile, a pH meter was used to measure the pH value of the reaction solution, and 4 M NaOH solution was added to adjust the pH of the reaction solution among 7.8–8.2. Sample volumes of 500 μl were removed at 4, 8, 12, 24, 36, 48, 60, 72 h, and then quenched, diluted to an appropriate concentration, centrifuged, and filtered as described above. After reaction, the remained PET substrate was collected by filtration through 0.45 μm jar-top filter units and dried at 50 °C before testing the crystallinity of the PET powder using DSC.

### Kinetic measurements of MHET hydrolysis

The enzyme reactions were performed in triplicate over 10 min using the purified enzyme (0.1–2 μM) and a MHET substrate (2–20 mM) in 50 mM sodium phosphate (pH 7.5) at 50 °C. Each reaction was terminated using an equal volume (600 μL) of 100% methanol. In the control group, 600 μL of methanol was added in advance to 300 μL of the enzyme solution to deactivate the enzyme. The product and substrate were quantified by HPLC. The initial reaction velocities were calculated from the TPA produced over time, and the kinetic parameters were determined by nonlinear regression of the initial velocities and substrate concentrations fitted to the Michaelis–Menten equation (Eq. 1).

5 $${{{\mbox{v}}}}_{0}/\left[{{\mbox{E}}}\right]={k}_{{{\mbox{cat}}}}\left[{{\mbox{S}}}\right]/\left({K}_{{{\mbox{m}}}}+\left[{{\mbox{S}}}\right]\right)$$

The Michaelis–Menten equation curve fittings used to determine the kinetic parameters of the WT Est30 and the variants are shown in Fig. S9.

### pH profile of MHET hydrolysis activity

For variant M8 (I171K/G130L), the pH dependency of MHET hydrolysis activity was determined at pH values of 6.0, 6.5, 7.0, 7.5, and 8.0. The enzyme reactions were performed in triplicate with 200 nM purified enzyme in 600 μL of 50 mM sodium phosphate buffer at the various pH values and at 50 °C. The MHET concentration was 4.8 mM. After 10 min, each reaction was terminated using an equal volume (600 μL) of 100% methanol.

### Temperature profile of MHET hydrolysis activity

The effect of temperature on MHET hydrolysis activity was determined at 40, 45, 50, 55, 60, 65, and 70 °C. The pH was 7.5 and the other conditions were the same as those used in the pH profile experiment.

### HPLC method

The contents of TPA, MHET, and BHET were determined using a CMB-20A system (Shimadzu) connected to an SPD-20A UV/Vis detector and a SunFire™ C18 column (5 μm, 4.6 × 250 mm) with a gradient comprising acetonitrile and 0.1% (v/v) formic acid in water at 30 °C after injection of each 10 µL sample. The acetonitrile content was increased from 5% to 44% over 13 min, and then to 70% after 18 min, at which point the ratio was kept constant for 5 min. TPA, MHET, and BHET were detected at 254 nm (Fig. S25) and quantified using calibration curves.

### Crystal structure determination

The Est30 variant KLS-MHETase was concentrated to 15 mg·mL^−1^ in 10 mM Tris-HCl (pH 8.0), 200 mM NaCl, and 5 mM dithiothreitol. Crystals were grown using the hanging-drop vapor diffusion method. Crystals of KLS-MHETase were grown at 18 °C by mixing an equal volume of the protein (15 mg·mL^−1^) with a reservoir solution containing 0.2 M zinc acetate tetrahydrate and 20% w/v polyethylene glycol 3350 (pH 6.4). The crystals appeared overnight and grew to a maximum size in approximately 2–3 days. The crystals were cryoprotected in a reservoir solution containing 20% glycerol before transfer to liquid nitrogen. All data were collected at Shanghai Synchrotron Radiation Facility beamlines BL02U1 and BL19U1, integrated and scaled using the HKL2000 package^96^. The initial model of KLS-MHETase was obtained by modeling using AlphaFold2^66^. The structure of KLS-MHETase was solved by molecular replacement, and was refined manually using COOT^97^. The structure was further refined with PHENIX^98^ using non-crystallographic symmetry and stereochemical information as constraints. The final structure was obtained through several rounds of refinement. The data collection and structure refinement statistics are summarized in Table S15. The accession number for the coordinate and structure factors reported in the present paper is PDB: 8ILT (KLS-MHETase).

### Melting temperature (*T*_m_) values of the enzymes determined by DSC

The melting temperature (*T*_m_) of each enzyme was determined using a MicroCal PEAQ DSC system (Malvern Panalytical, Malvern, UK). The purified proteins were diluted to 0.5–1.0 mg·mL^−1^ in 50 mM sodium phosphate buffer (pH 7.5), and degassed immediately prior to testing. The samples were heated from 30 to 120 °C at a rate of 3 °C per min during the measurement. The baseline value of the buffer without protein was removed from the protein trace. Each *T*_m_ value is the average of two measurements.

### Analytical method for determining PET crystallinity

DSC was used to determine the crystallinity of each PET sample investigated in the present paper. The PET sample (4–6 mg) was placed on an aluminum pan with a solid sample cover. It was first heated from 30 to 300 °C at 10 °C·min^−1^, maintained at 300 °C for 1 min, cooled from 300 to 30 °C at −10 °C·min^−1^, and held at 30 °C for 1 min in a DSC 8000 system (PerkinElmer) with an automatic liquid nitrogen chiller. The enthalpy of melting (ΔH_*m*_) and the enthalpy of cold crystallization (ΔH_*cc*_) were used to determine the percentage crystallinity during the heating scan, according to the following equation (Eq. 2):

6 $$\% {{\mbox{crystallinity}}}=[\Delta {H}_{m}-\Delta {H}_{{cc}}]/\Delta {H}_{m}^{^\circ }\times 100 \%$$

where $\Delta {H}_{m}^{^\circ }$ is the enthalpy of melting of a 100% crystalline PET sample (i.e., 140.1 J·g^−1^)^99^. Each crystallinity value is the average of two measurements.

### Statistics and reproducibility

Enzyme kinetic data, MHET hydrolysis assay activity, and PET hydrolysis assay activity were obtained from the average of three parallel experiments. The feature values for the 1 ns and 5 ns MD simulations were averaged over 10 and 5 replications, respectively.

### Reporting summary

Further information on research design is available in the Nature Portfolio Reporting Summary linked to this article.

### Supplementary information

Peer Review File

Supplementary Information

Description of Supplementary Materials

Supplementary Data 1

Supplementary Data 2

Reporting Summary

## Data Availability

The data that support this study are available from the corresponding author (Y.Z.) upon request. The source data underlying Figs. 2–8 are provided as a Supplementary Data file. The atomic coordinates and structure factors have been deposited in the Protein Data Bank, (https://www.wwpdb.org/) with PDB ID code 8ILT. The design results of the experimental mutants by PRODA and AlphaFold models for enzyme complexes are available at https://github.com/zhangjun19thu/SI_Structures.
