# Supplementary material for: Computational design of highly efficient thermostable MHET hydrolases and dual enzyme system for PET recycling
Source: Commun Biol. 2023 Nov 9;6:1135. doi: 10.1038/s42003-023-05523-5 (PMC10636135; doi:10.1038/s42003-023-05523-5)
Supplement: Supplementary file 2 — Supplementary Information [file 42003_2023_5523_MOESM2_ESM.pdf]

Supplementary Information

Computational design of highly efficient thermostable MHET hydrolases and dual enzyme system for PET recycling

Jun Zhang[a,b,#], Hongzhao Wang [a,#], Zhaorong Luo [c,#], Zhenwu Yang[a], Zixuan Zhang[a], Pengyu Wang[b], Mengyu Li[a], Yi Zhang[c], Yue Feng[c], Diannan Lu[*,b], Yushan Zhu[*,a,d]

[a] College of Life Science and Technology, Beijing University of Chemical Technology, Beijing 100029, China

[b] Department of Chemical Engineering, Tsinghua University, Beijing 100084, China

[c] Beijing Advanced Innovation Center for Soft Matter Science and Engineering, State Key Laboratory of Chemical Resource Engineering, Beijing University of Chemical Technology, Beijing 100029, China

[d] National Energy R&D Center for Biorefinery, Beijing University of Chemical Technology, Beijing 100029, China

[#] These authors contribute equally.

[*] Corresponding Authors. E-mail: ludiannan@tsinghua.edu.cn ; zhuys@mail.buct.edu.cn

Table of Contents

Supplementary Tables 3
Supplementary Figures 29
Supplementary Methods 55
References 59

Supplementary Tables

Table S1. The catalytic activity and thermal stability of the reported MHET hydrolytic enzymes.

Enzyme	Microbial source	GenBank code	PDB ID	Reaction temperature(°C)	Reaction pH	k_{cat} (s^{-1})	K_m (mM)	k_{cat}/K_m ($mM^{-1}s^{-1}$)	T_m (°C)	Ref.
*Is*MHETase	Ideonella sakaiensis	GAP38911.1	6QZ3	30	7.5	50.28	0.023	2186.1	50.6[1]	[2]
CALB	Candida antarctica	Z30645	5A71	50/60	7.0	—	—	—	58[3]	[4]
TfCa	Thermobifida fusca	AAZ54921	7W1K	50	8.0	0.014 ±5.2E-5	1.1±0.087	0.01	80	[5]
TTCE	Thermus thermophilus	—	1UFO	60	8.0	3 folds lower than LCC	—	—	80	[6]
feruloyl esterase	Comamonas thiooxydans	WP_080747404.1	—	30	7.5	40.181	0.175	229.6	—	[2]
feruloyl esterase	Hydrogenophaga sp. PML113	WP_083293388.1	—	30	7.5	5.342	0.041	130.29	—	[2]
BsCE	Bacillus subtilis	P37967.2	1QE3	50	8.0	0.056± 0.015	1.3± 0.51	0.04	56	[5]
Bs2Est	Bacillus subtilis	P37967	—	30	7.5	6.46	5.10	1.27	—	[7]
Mle046	uncultured bacterium	QXL91156	—	30	7.5	80.9	2.6	31.1	—	[8]
KL-MHETase	Geobacillus stearothermophilus	—	—	50	7.5	21.43	4.56	4.70	67.58	this study

Table S2. The protein library constructed for scaffold selection.

Substrate	Active site residues [a]	PDB ID of scaffolds [b]
Mono(2-hydroxyethyl) terephthalate (MHET)	Ser1, His2, Asp3, Ala4, Ala5, Ser6	1C7I,1EE8,1SH7,4AEE,1ZJC,1HIZ,2QR5,1JJI,1UI1,3ORW,1EVQ,4RG8,4ICS,4UHF,1NP2,5AXG,5Y3X,6JHP,4EPQ,3X2E,2W11,4WY5,4W5U,1WZA,3B7M,6ANE,4WY8,1UMG,4FDM,1SDO,1KWG,4PVA,5NG7,2C7B,7BFR,6WNI,6NZS,7FBT,5NFQ,3AIK,3RJY,1DC1,3P1Y,3FZI,6V6Y,6LTQ,1N82,3TK1,3B01,2PLQ,7CTR,5GY0,2D3Y,2D0A,7CF6,6Y2K,6DG4,3W24,4KFR,4MKI,3ZDJ,4V2I,4X0V,4FNQ,4EL8,2WSP,1TQH,5C0Q,4C1P,4D2I,3TEO,6FAO,5FBY,3UG5,5FRD,5AY7,1DK4,1H0B,3T2G,2BS9,1Y0R,4UQ9,3W6L,3WVJ,1B0I,4B88,1L1Y,5DT5,4E2O,5A5L,1VK2,4PTX,2YGK,1KEA,1VBR,6IFE,4XUP,2VUJ,4OJY,5WVU,3W7V,4DOE,5FOS,4CJ1

[a] These residues correspond to those in the active-site matching model (Figure 2A).
[b] A total of 104 unique scaffolds were collected.

Table S3. Geometrical catalytic constraints for scaffold selection.

Interacting Pair	Constraint Type	Atom1 [a]	Atom2 [a]	Atom3 [a]	Atom4 [a]	Standard Value [b]	Deviation
Ser1-TS	Distance	OG	#C11			2.2 Å	0.2 Å
	Angle	CB	OG	#C11		100°	10°
	Angle	OG	#C11	#O4		110°	10°
	Torsion	OG	#O4	#C11	#O3	120°	10°
His2-Ser1	Distance	#OG	NE2			2.7 Å	0.1 Å
	Angle	#CB	#OG	NE2		102°	10°
	Angle	#OG	NE2	CD2		170°	10°
Asp3-His2	Distance	#ND1	OD2			2.7 Å	0.4 Å
	Distance	#HD1	OD2			2.0 Å	0.6 Å
	Angle	#CG	#ND1	OD2		124°	20°
	Angle	#ND1	OD2	CG		102°	30°
	Angle	#ND1	#HD1	OD2		146°	30°
Ala4-TS	Distance	#O4	HN			1.9 Å	0.4 Å
	Angle	#C11	#O4	HN		124°	10°
	Angle	#O4	HN	N		160°	20°
Ala5-TS	Distance	#O4	HN			1.8 Å	0.4 Å
	Angle	#C11	#O4	HN		95°	10°
	Angle	#O4	HN	N		155°	20°
Ser6- TS	Distance	#O2	OG			2.6 Å	0.6 Å
	Angle	#C10	#O2	OG		120°	30°
	Angle	#O2	OG	CB		110°	30°

[a] Atoms on the latter residue within a site pair are prefixed with '#'.

[b] The distance and angle parameters are determined based on the crystal structure of natural MHETase (PDB ID: 6QGA[9] or 6JTT[1]) and the complex structures of serine proteases and their transition state analogues[10].

Table S4. Scaffolds selected by ProdaMatch for MHET hydrolysis.

PDB code	Matched_sites[a]	Vdw_backbone[b]	Vdw_self[c]	Catacons[d]	Total_score[e]	Match_num[f]	Temp[g]/℃
1EVQ	A155 A282 A210 A84 A83 A252	39.51	28.95	1.53	11.05	3	70[11]
1TQH	A94 A223 A130 A25 A95 A193	19.06	20.55	2.32	12.40	5	70[12]
4WY8	A164 A291 A213 A90 A165 A261	30.51	27.33	2.36	13.23	6	50[13]
5FRD	A89 A228 A202 A90 A30 A200	27.25	22.61	2.63	14.06	8	80[14]
4UHF	A101 A250 A105 A35 A102 A222	68.80	19.61	2.03	14.60	6	80[15]
2C7B	A154 A281 A214 A82 A83 A251	33.33	18.58	2.95	15.90	2	80[16]
4V2I	A158 A285 A211 A87 A86 A255	31.94	34.00	3.03	16.39	1	45[17]
1C7I	A189 A399 A273 A107 A190 A310	61.51	17.54	5.77	28.68	1	60[18]

[a] Matched_sites represents the matched positions of the catalytic residues in the active site model.

[b] The VDW_backbone represents the penalty of the collision between TS and the protein backbone.

[c] The VDW_self represents the penalty of the repulsion between the catalytic residue and TS and the repulsion between the catalytic residues.

[d] The Catacons score represents the penalty of the deviation of the catalytic constraint variable from its optimal value, and is calculated by a harmonic potential, as $v(c) = (c - c_0)^2$, where c_0 is the optimal value of the constraint variable c. Here, the constraint variable c refers to bond length, angle, or torsion.

[e] The Total_score is a linear weighted average of the collision energy scores and the catalytic constraint score. A lower score represents a better match.

[f] Match_num represents the number of matchs identified in the protein scaffold.

[g] Optimal temperature reported in the literature.

Table S5. Variation rules for generating TS library.

Type	Atom1[a]	Atom2[a]	Atom3[a]	Atom4[a]	Min[b]	Max[b]	Step[b]
Distance	#OG	C11			1.60	2.20	0.10
Angle	#CB	#OG	C11		105.0	115.0	2.5
Torsion	#CA	#CB	#OG	C11	-180.0	179.0	20.0
Angle	#OG	C11	O4		105.0	115.0	2.5
Torsion	#CB	#OG	C11	O4	-180.0	179.0	20.0
Torsion	O3	C11	C4	C3	-180.0	179.0	30.0
Torsion	C6	C1	C10	O1	-15.0	15.0	15.0
Torsion	C4	C11	O3	C7	-180.0	179.0	30.0
Torsion	C11	O3	C7	C8	-180.0	179.0	30.0
Torsion	O3	C7	C8	O9	-180.0	179.0	30.0

[a] Atoms on anchored residue Ser94 are prefixed with '#'.

[b] Distance measurements are given in Å. Angle and torsion measurements are given in degrees.

Table S6. Catalytic geometrical constraints for design.

Interacting Pair	Constraint Type	Atom1 [a]	Atom2 [a]	Atom3 [a]	Atom4 [a]	Min Value [c]	Max Value [c]
Ser94-TS	Distance	OG	#C11			1.6 Å	2.6 Å
	Angle	OG	#C11	#O4		80°	140°
Hsd223-Ser94	Distance	NE2	#OG			2.6 Å	3.6 Å
	Angle	HE2	#OG	#CB		90°	130°
	Angle	NE2	HE2	#OG		140°	180°
Hsd223-Asp193 [b]	Distance	ND1	#OD1			2.6 Å	3.4 Å
	Angle	HD1	#OD1	#CG		100°	140°
	Angle	ND1	HD1	#OD1		140°	180°
Phe25-TS	Distance	N	#O4			2.6 Å	3.2 Å
	Angle	HN	#O4	#C11		100°	140°
	Angle	H	HN	#O4		140°	180°
Leu95-TS	Distance	N	#O4			2.6 Å	3.2 Å
	Angle	HN	#O4	#C11		100°	140°
	Angle	H	HN	#O4		140°	180°

[a] Atoms on the latter residue within a site pair are prefixed with '#'

[b] Either site pair of Hsd223-Asp193 is needed.

[c] The distance and angle parameters are determined based on the crystal structure of natural MHETase (PDB ID: 6QGA[9] or 6JTT[1]) and the complex structures of serine proteases and their transition state analogues[10].

Table S7. Scheme for the active-site redesign in scaffold 1TQH.

Scaffold	Substrate	Catalytic residues	Binding residues	
			Sequence selection residues	Conformation optimization residues
1TQH	Mono(2-hydroxyethyl) terephthalate	S94[a], H223[a], D193[a], F25[b], L95[b]	T26, K122, M127, G130, L167, I171, M195, I196	H23, N28, D31, L93, M115, C116, I121, S123, T126, V131, Q168, E194, V224

[a] S94, H223 and D193 are the catalytic triad residues.

[b] F25 and L95 are the oxyanion hole residues.

Table S8. Calculated free energy changes for single variants compared to wild type Est30. The unit of $\Delta\Delta G_{bind}$ and $\Delta\Delta G_{fold}$ is in kcal/mol.

Index	Mutations	$\Delta\Delta G_{bind}$ [a]	$\Delta\Delta G_{fold}$ [b]	Index	Mutations	$\Delta\Delta G_{bind}$ [a]	$\Delta\Delta G_{fold}$ [b]
1	G130H	-1.76	-11.37	53	T26L	-2.40	8.88
2	G130V	-1.57	-8.77	54	L167R	-3.12	9.65
3	G130I	4.10	-14.44	55	M127I	-0.69	7.36
4	G130L	4.00	-14.31	56	T26V	-2.56	9.27
5	G130F	-5.25	-4.40	57	M127Q	2.05	4.69
6	G130C	-1.94	-6.61	58	L167A	-1.06	7.82
7	G130M	3.55	-11.65	59	I171F	-3.39	10.29
8	G130T	-1.49	-6.27	60	T26A	-1.81	8.73
9	G130A	-1.84	-5.56	61	L167T	-1.69	8.73
10	G130S	-1.77	-5.01	62	M127T	-0.63	8.05
11	G130W	-4.32	-1.87	63	I196A	-1.01	8.49
12	G130N	-1.55	-3.85	64	I171T	-0.82	8.55
13	L167F	-4.91	1.42	65	K122N	6.32	1.42
14	M127K	-7.83	5.46	66	I171C	-1.10	8.97
15	G130Q	4.42	-6.20	67	I171W	4.67	3.25
16	G130Y	3.10	-4.19	68	I171A	-1.05	8.98
17	I171K	-5.55	4.85	69	M195Y	-1.95	10.39
18	L167K	-2.15	2.38	70	I196S	-0.85	9.48
19	L167M	-1.10	2.07	71	L167S	-1.48	10.14
20	I171M	-2.39	3.53	72	L167Q	0.19	8.61
21	M195V	-0.23	1.91	73	M127Y	10.07	-1.23
22	M195L	-0.76	2.44	74	M195G	0.80	8.08
23	T26M	-2.52	4.39	75	I171S	-0.96	10.10
24	M127H	4.19	-1.98	76	M127N	0.22	8.96
25	M195A	0.49	1.73	77	K122L	5.15	4.10
26	M195I	-0.71	3.68	78	I171N	-0.81	10.32
27	L167H	-1.39	4.58	79	L167N	-1.22	11.10
28	I171V	-1.27	4.51	80	M195F	2.09	8.04
29	I171R	-8.00	11.25	81	K122V	5.70	5.06
30	M195T	0.09	3.26	82	K122S	6.41	4.53
31	L167W	4.68	-1.07	83	K122M	4.89	6.06
32	M195S	0.67	3.34	84	I171Y	-2.87	13.86
33	M127V	-0.62	4.80	85	K122C	6.30	4.84
34	L167V	-1.93	6.29	86	K122A	6.36	5.02
35	I196V	-1.36	6.07	87	K122I	5.08	6.65
36	I171L	-1.51	6.24	88	K122T	5.37	8.13
37	T26S	-1.54	6.33	89	T26G	-1.61	15.68
38	K122Q	5.46	-0.12	90	M127G	-0.04	14.15
39	I171H	-1.10	6.55	91	M127R	10.44	3.96
40	I196T	-1.19	6.72	92	I196G	-0.96	15.51
41	M127S	-2.96	8.54	93	L167G	-1.70	16.75
42	I171Q	-4.13	9.82	94	I171G	-1.06	16.57
43	L167Y	1.35	4.49	95	T26W	-1.38	17.63
44	K122H	6.15	-0.31	96	M127W	21.89	-4.04

45	T26I	-3.02	8.95	97	K122G	6.45	11.82
46	M127C	-0.31	6.27	98	T26Y	8.26	14.05
47	M195W	-3.86	10.02	99	I196L	-1.09	24.85
48	M127L	7.23	-1.02	100	I196W	31.03	6.41
49	L167C	-1.71	7.94	101	T26F	-5.04	54.73
50	L167I	-1.13	7.50	102	I196M	-2.71	61.14
51	M127A	-0.17	6.55	103	I196Y	44.29	17.07
52	M127F	7.35	-0.91	104	I196F	3.83	91.03

[a] $\Delta\Delta G_{bind}$ is the difference of the binding energy of the enzyme−substrate complex in the TS of variant minus that of wild type.

[b] $\Delta\Delta G_{fold}$ is the difference of the folding energy of the enzyme−substrate complex in the TS of variant minus that of wild type.

Table S11. The catalytic indicators and computed free energy changes of the variants with a large enhancement of the indicators screened by MD-based evaluation. The indicators are the average of those in five independent 5 ns MD simulations.

Variants*	Fre_dist [a]	Fre_rmsd [b]	Fre_oxy1 [c]	Fre_oxy2 [d]	Fre_K122 [e]	Fre_intro [f]	$\Delta\Delta G_{bind}$ [g]	$\Delta\Delta G_{fold}$ [g]
WT	11.66%	2.28%	10.18%	4.59%	34.28%	0.00%	———	——
G130F	30.98%	15.29%	30.50%	18.20%	52.89%	0.00%	-5.25	-4.40
G130L	15.45%	4.75%	24.95%	6.47%	43.27%	0.00%	4.00	-14.31
L167K	52.85%	21.08%	67.82%	46.55%	84.43%	17.09%	-2.15	2.38
I171K	24.87%	8.94%	27.98%	22.20%	44.71%	27.98%	-5.55	4.85
I171R	41.60%	26.99%	37.76%	34.05%	64.55%	63.87%	-8.00	11.25
M127S	21.92%	12.02%	25.07%	20.56%	54.33%	32.30%	-2.96	8.54
M127K	16.97%	16.17%	29.46%	11.50%	33.73%	97.41%	-7.83	5.46
I171K_G130L	55.01%	63.51%	64.91%	56.45%	69.62%	66.75%	-0.08	-12.01
M127S_G130F	62.12%	57.29%	70.86%	61.64%	62.87%	58.12%	-7.96	1.31
M127K_G130T	43.27%	50.18%	50.02%	42.55%	37.00%	91.30%	-7.84	-3.30
I171R_G130F	69.02%	81.56%	83.35%	69.58%	61.44%	79.92%	-11.77	4.43
M195L_M127S	47.43%	32.30%	48.98%	45.23%	51.42%	45.59%	-1.96	4.82
I171M_G130H	46.79%	11.98%	47.35%	33.69%	65.39%	0.00%	-2.67	-10.33
L167F_I171L	33.81%	23.07%	36.65%	31.50%	46.99%	0.00%	-2.89	3.53
M195V_M127S	34.93%	17.52%	34.53%	30.02%	37.33%	33.41%	-0.63	4.61
L167K_G130F	44.07%	32.46%	55.49%	26.43%	61.84%	52.69%	-5.92	-4.52
M195L_L167K	41.92%	21.44%	60.44%	35.09%	73.13%	4.83%	-1.39	1.76
M195T_L167K	44.87%	11.66%	53.37%	32.85%	67.78%	39.76%	-0.57	2.63
M195S_L167K	37.33%	10.98%	34.73%	26.75%	63.83%	33.01%	0.03	2.75
L167K_M127H	43.55%	45.31%	43.39%	40.56%	71.78%	76.21%	3.85	-0.20
I171R_G130H	54.17%	58.08%	61.60%	56.41%	52.69%	80.00%	-8.28	-2.53
L167M_I171R	32.02%	36.13%	37.05%	34.69%	60.40%	89.50%	-6.69	7.40
I171R_M127S	44.47%	50.42%	48.50%	45.15%	42.08%	99.88%	-8.85	11.08
I171R_M127C	40.20%	49.90%	50.90%	42.51%	70.06%	90.66%	-6.21	9.01
M195V_I171R	52.93%	47.50%	58.48%	54.01%	66.83%	80.00%	-5.22	8.35
I171R_T26M	55.45%	53.53%	52.53%	50.14%	63.47%	97.84%	-7.56	10.91
I171R_G130C	46.55%	19.20%	51.22%	43.91%	75.85%	71.34%	-8.46	2.23
M195S_I171R	48.26%	42.59%	54.21%	47.86%	62.99%	68.78%	-4.33	9.82
I171K_G130H	41.16%	32.69%	53.05%	35.93%	64.43%	69.18%	-5.83	-8.98
L167F_M127K	30.14%	27.74%	56.69%	22.87%	95.29%	44.99%	-9.63	2.00
I171K_G130C	28.26%	19.12%	37.09%	24.39%	43.97%	46.19%	-6.02	-4.18
I171K_G130W	49.34%	35.05%	40.36%	29.14%	84.93%	50.10%	-8.57	0.69
I171K_M127K	52.73%	57.64%	58.32%	54.81%	19.36%	99.36%	-9.79	5.67
I171K_M127S	32.46%	26.63%	38.08%	29.34%	67.11%	35.61%	-7.85	10.92
L167H_M127K	64.39%	70.34%	76.89%	65.55%	47.98%	89.06%	-5.57	4.34
M195A_M127K	26.79%	29.98%	35.69%	25.35%	50.10%	82.99%	-3.61	0.90
M127K_G130W	24.27%	30.66%	29.70%	22.75%	44.95%	91.18%	-10.67	1.16

M195W_L167F_G130M	24.83%	21.40%	31.54%	23.91%	44.15%	0.00%	-5.28	-9.65
M195W_L167F_G130V	34.33%	22.28%	44.11%	24.83%	92.69%	0.00%	-5.85	-7.95
I196V_L167F_G130M	50.26%	30.14%	41.88%	38.60%	92.50%	0.00%	-4.38	-7.76
M195I_L167F_G130C	34.41%	22.59%	33.97%	27.62%	61.52%	0.00%	-2.98	-8.86
I171K_M127S_G130F	48.06%	58.72%	52.77%	47.70%	45.67%	98.48%	-14.98	6.24
I171K_M127S_G130L	34.45%	39.24%	42.00%	26.23%	51.54%	77.33%	-8.01	-3.43

[a] The frequency of the nucleophilic attack distance between the atom OG of Ser94 and the atom C11 of TS less than 3.0 Å.

[b] The frequency of the all-atom RMSD value of TS less than 2.5 Å, calculated with the first frame of the MD simulation as the reference.

[c] The frequency of hydrogen bond formed between the main-chain N of F25 and the atom O4 of TS.

[d] The frequency of hydrogen bond formed between the main-chain N of L95 and the atom O4 of TS.

[e] The frequency of hydrogen bond formed between the atom NZ of K122 and the carboxyl atom O1 or O2 of TS.

[f] The frequency of hydrogen bond formed between the polar residue introduced by mutation and the carboxyl atom O1 or O2 of TS.

[g] The difference of the binding energy and the folding energy of variant minus that of wild type.

[*] The experimental variants are colored in orange. The darker green color indicates that the indicators of the variants were improved. The hydrogen bond is defined as: the bond length (Donor–Acceptor) is less than 3.5 Å and the bond angle (Donor–H–Acceptor) is greater than 120 °.

Table S12. MD-based evaluation of wild-type Est30 and 14 experimentally tested variants. The indicators are average of those in five independent 5 ns MD simulations.

Variants	Mutations	Fre_dist [a]	Fre_rmsd [b]	Fre_oxy1 [c]	Fre_oxy2 [d]	Fre_K122 [e]	Fre_intro [f]
WT	——	11.66%	2.28%	10.18%	4.59%	34.28%	0.00%
M1	G130L	15.45%	4.75%	24.95%	6.47%	43.27%	0.00%
M2	I171K	24.87%	8.94%	27.98%	22.20%	44.71%	27.98%
M3	G130F	30.98%	15.29%	30.50%	18.20%	52.89%	0.00%
M4	L167K	52.85%	21.08%	67.82%	46.55%	84.43%	17.09%
M5	I171R	41.60%	26.99%	37.76%	34.05%	64.55%	63.87%
M6	M127S	21.92%	12.02%	25.07%	20.56%	54.33%	32.30%
M7	I171R/G130F	69.02%	81.56%	83.35%	69.58%	61.44%	79.92%
M8	I171K/G130L	55.01%	63.51%	64.91%	56.45%	69.62%	66.75%
M9	M127K/G130T	43.27%	50.18%	50.02%	42.55%	37.00%	91.30%
M10	M195L/M127S	47.43%	32.30%	48.98%	45.23%	51.42%	45.59%
M11	I171M/G130H	46.79%	11.98%	47.35%	33.69%	65.39%	0.00%
M12	M127S/G130F	62.12%	57.29%	70.86%	61.64%	62.87%	58.12%
M13	I171K/G130F/M127S	48.06%	58.72%	52.77%	47.70%	45.67%	98.48%
M14	I171K/G130L/M127S	34.45%	39.24%	42.00%	26.23%	51.54%	77.33%

[a] The frequency of the nucleophilic attack distance between the atom OG of Ser94 and the atom C11 of TS less than 3.0 Å.

[b] The frequency of the all-atom RMSD value of TS less than 2.5 Å, calculated with the first frame of the MD simulation as the reference.

[c] The frequency of hydrogen bond formed between the main-chain N of F25 and the atom O4 of TS.

[d] The frequency of hydrogen bond formed between the main-chain N of L95 and the atom O4 of TS.

[e] The frequency of hydrogen bond formed between the atom NZ of K122 and the carboxyl atom O1 or O2 of TS.

[f] The frequency of hydrogen bond formed between the polar residue introduced by mutation and the carboxyl atom O1 or O2 of TS.

Table S13. Kinetic parameters of wild type Est30 and all designed variants, as well as FAST-PETase, LCC-ICCG and the fusion enzymes towards MHET hydrolysis at 50 °C in 50 mM sodium phosphate buffer (pH 7.5).

Variants	Mutations	k_{cat} (s^{-1})	K_m (mM)	k_{cat}/K_m ($mM^{-1}s^{-1}$)	Fold increase in k_{cat}/K_m	T_m (°C)
WT	——	1.17±0.14	8.98±2.49	0.13	1.0	74.07±0.14
M1	G130L	2.97±0.21	5.36±1.13	0.55	4.2	76.39±0.36
M2	I171K	12.08±6.87	18.40±3.47	0.66	5.1	65.08±0.33
M3	G130F	0.75±0.05	1.12±0.45	0.66	5.1	75.82±0.25
M4	L167K	ND	ND	ND	ND	69.33±0.36
M5	I171R	1.18±0.20	8.90±2.71	0.13	1.0	63.90±0.26
M6	M127S	1.27±0.07	5.38±0.71	0.24	1.8	69.64±0.33
M7	I171R/G130F	2.80±0.27	8.40±1.95	0.33	2.5	66.18±0.21
**M8**	**I171K/G130L**	**21.43±1.12**	**4.56±0.75**	**4.70**	**36.0**	**67.58±0.13**
M9	M127K/G130T	2.72±0.24	5.35±1.39	0.51	3.9	68.69±0.16
M10	M195L/M127S	1.00±0.11	5.44±1.76	0.18	1.4	69.37±0.37
M11	I171M/G130H	0.56±0.05	9.15±1.87	0.06	0.5	73.30±0.26
M12	M127S/G130F	1.41±0.07	3.72±0.48	0.38	2.9	71.33±0.19
**M13**	**I171K/G130F/M127S**	**20.99±0.83**	**2.59±0.41**	**8.11**	**62.1**	**63.29±0.23**
**M14**	**I171K/G130L/M127S**	**21.76±0.62**	**1.73±0.24**	**12.58**	**96.3**	**63.18±0.20**
FAST-PETase	——	2.34±0.14	33.87±2.82	0.07	——	67.80[19]
LCC-ICCG*	——	1.41±0.13	20.13±3.69	0.07	——	94.00[20]
KL36F	——	16.45±0.79	4.52±0.67	3.64	——	68.67±0.11
KL28F	——	13.99±0.74	3.92±0.69	3.57	——	68.78±0.17
KL20F	——	13.97±0.65	3.96±0.61	3.53	——	68.86±0.35
KLS20F	——	20.20±0.82	1.65±0.34	12.27	——	65.90±0.29

* The reaction temperature for LCC-ICCG was 70 °C.

Table S14. Protein sequences of wild type Est30 and the designed variants.

Identifier	Number of mutations	Mutations	Expressed amino acid sequence*
Est30-WT	0	None	MMKIVPPKPFFFEAGERAVLLLHGFTGNSADVRMLGRF<br>LESKGYTCHAPIYKGHGVPPEELVHTGPDDWWQDVMN<br>GYEFLKNKGYEKIAVAGLSLGGVFSLKLGYTVPIEGIVT<br>MCAPMYIKSEETMYEGVLEYAREYKKREGKSEEQIEQE<br>MEKFKQTPMKTLKALQELIADVRDHLDLIYAPTFVVQA<br>RHDEMINPDSANIIYNEIESPVKQIKWYEQSGHVITLDQE<br>KDQLHEDIYAFLESLDWLEHHHHHH
Est30-KL	2	I171K/G130L	MMKIVPPKPFFFEAGERAVLLLHGFTGNSADVRMLGRF<br>LESKGYTCHAPIYKGHGVPPEELVHTGPDDWWQDVMN<br>GYEFLKNKGYEKIAVAGLSLGGVFSLKLGYTVPIEGIVT<br>MCAPMYIKSEETMYE**L**VLEYAREYKKREGKSEEQIEQE<br>MEKFKQTPMKTLKALQEL**K**ADVRDHLDLIYAPTFVVQ<br>ARHDEMINPDSANIIYNEIESPVKQIKWYEQSGHVITLDQ<br>EKDQLHEDIYAFLESLDWLEHHHHHH
Est30-KFS	3	I171K/G130F/M127S	MMKIVPPKPFFFEAGERAVLLLHGFTGNSADVRMLGRF<br>LESKGYTCHAPIYKGHGVPPEELVHTGPDDWWQDVMN<br>GYEFLKNKGYEKIAVAGLSLGGVFSLKLGYTVPIEGIVT<br>MCAPMYIKSEET**S**YE**F**VLEYAREYKKREGKSEEQIEQE<br>MEKFKQTPMKTLKALQEL**K**ADVRDHLDLIYAPTFVVQ<br>ARHDEMINPDSANIIYNEIESPVKQIKWYEQSGHVITLDQ<br>EKDQLHEDIYAFLESLDWLEHHHHHH
Est30-KLS	3	I171K/G130L/M127S	MMKIVPPKPFFFEAGERAVLLLHGFTGNSADVRMLGRF<br>LESKGYTCHAPIYKGHGVPPEELVHTGPDDWWQDVMN<br>GYEFLKNKGYEKIAVAGLSLGGVFSLKLGYTVPIEGIVT<br>MCAPMYIKSEET**S**YE**L**VLEYAREYKKREGKSEEQIEQE<br>MEKFKQTPMKTLKALQEL**K**ADVRDHLDLIYAPTFVVQ<br>ARHDEMINPDSANIIYNEIESPVKQIKWYEQSGHVITLDQ<br>EKDQLHEDIYAFLESLDWLEHHHHHH
FAST-PETase	0	None	MNPYARGPNPTAASLEASAGPFTVRSFTVSRPSGYGAGT<br>VYYPTNAGGTVGAIAIVPGYTARQSSIKWWGPRLASHG<br>FVVITIDTNSTLDQPESRSSQQMAALRQVASLNGTSSSPI<br>YGKVDTARMGVMGWSMGGGGSLISAANNPSLKAAAP<br>QAPWHSSTNFSSVTVPTLIFACENDSIAPVNSSALPIYDS<br>MSQNAKQFLEIKGGSHSCANSGNSNQALIGKKGVAWM<br>KRFMDNDTRYSTFACENPNSTAVSDFRTANCSLEHHHH<br>HH

* Letters in red bold depict mutations relative to the original scaffold.

Table S15. Crystallographic data collection and refinement statistics of KLS-MHETase.

	KLS-MHETase (PDB: 8ILT)
**Data collection**	
Space group	P21
Cell dimensions	
a, *b*, *c* (Å)	150.0, 60.0, 166.0
α / β /γ (°)	90.00, 99.24, 90.00
Resolution (Å)	49.49-2.42 (2.48-2.42)
R_{sym} or R_{merge} (%)	19.9 (94.9)
$I/\sigma(I)$	10.8 (2.1)
Completeness (%)	100.0 (100.0)
Redundancy	6.7 (6.8)
**Refinement**	
Resolution (Å)	25.48-2.42 (2.51-2.42)
Unique reflection	111792 (11039)
R_{work} / R_{free}[#]	0.2247/0.2766
No. atoms	18674
Protein	18257
Ligand/ion	0
Water	417
B factors	54.15
Protein	54.46
Ligand/ion	
Water	40.41
R.m.s. deviations	
Bond lengths (Å)	0.008
Bond angles (°)	1.46

For each structure one crystal was used. Values in parentheses are for highest-resolution shell.

[#]R_{free} was calculated with 5 % of the reflections selected.

Table S16. The amino acid sequences of the constructed fusion enzymes.

Identifier	Linker sequence	Length of linker(aa)	Expressed amino acid sequences
KL20F	GGGSGGSG<br>GGSGGGSG<br>GSGS	20	MMKIVPPKPFFFEAGERAVLLLHGFTGNSADVRMLGRFLESKGYTCHAP<br>IYKGHGVPPEELVHTGPDDWWQDVMNGYEFLKNKGYEKIAVAGLSLG<br>GVFSLKLGYTVPIEGIVTMCAPMYIKSEETMYELVLEYAREYKKREGKSE<br>EQIEQEMEKFKQTPMKTLKALQELKADVRDHLDLIYAPTFVVQARHDE<br>MINPDSANIIYNEIESPVKQIKWYEQSGHVITLDQEKDQLHEDIYAFLESL<br>DW**GGGSGGSGGGSGGGSGGSGS**NPYARGPNPTAASLEASAGPFTVRSF<br>TVSRPSGYGAGTVYYPTNAGGTVGAIAIVPGYTARQSSIKWWGPRLASH<br>GFVVITIDTNSTLDQPESRSSQQMAALRQVASLNGTSSSPIYGKVDTARM<br>GVMGWSMGGGGSLISAANNPSLKAAAPQAPWHSSTNFSSVTVPTLIFAC<br>ENDSIAPVNSSALPIYDSMSQNAKQFLEIKGGSHSCANSGNSNQALIGKK<br>GVAWMKRFMDNDTRYSTFACENPNSTAVSDFRTANCSLEHHHHHH
KL28F	GGGSGGSG<br>GGSGGGSG<br>GSGSGGSG<br>GSGS	28	MMKIVPPKPFFFEAGERAVLLLHGFTGNSADVRMLGRFLESKGYTCHAP<br>IYKGHGVPPEELVHTGPDDWWQDVMNGYEFLKNKGYEKIAVAGLSLG<br>GVFSLKLGYTVPIEGIVTMCAPMYIKSEETMYELVLEYAREYKKREGKSE<br>EQIEQEMEKFKQTPMKTLKALQELKADVRDHLDLIYAPTFVVQARHDE<br>MINPDSANIIYNEIESPVKQIKWYEQSGHVITLDQEKDQLHEDIYAFLESL<br>DW**GGGSGGSGGGSGGGSGGSGSGGSGGSGS**NPYARGPNPTAASLEAS<br>AGPFTVRSFTVSRPSGYGAGTVYYPTNAGGTVGAIAIVPGYTARQSSIKW<br>WGPRLASHGFVVITIDTNSTLDQPESRSSQQMAALRQVASLNGTSSSPIY<br>GKVDTARMGVMGWSMGGGGSLISAANNPSLKAAAPQAPWHSSTNFSS<br>VTVPTLIFACENDSIAPVNSSALPIYDSMSQNAKQFLEIKGGSHSCANSGN<br>SNQALIGKKGVAWMKRFMDNDTRYSTFACENPNSTAVSDFRTANCSLE<br>HHHHHH
KL36F	GGGSGGSG<br>GGSGGGSG<br>GSGSGGSG<br>GSGSGGSG<br>GSGS	36	MMKIVPPKPFFFEAGERAVLLLHGFTGNSADVRMLGRFLESKGYTCHAP<br>IYKGHGVPPEELVHTGPDDWWQDVMNGYEFLKNKGYEKIAVAGLSLG<br>GVFSLKLGYTVPIEGIVTMCAPMYIKSEETMYELVLEYAREYKKREGKSE<br>EQIEQEMEKFKQTPMKTLKALQELKADVRDHLDLIYAPTFVVQARHDE<br>MINPDSANIIYNEIESPVKQIKWYEQSGHVITLDQEKDQLHEDIYAFLESL<br>DW**GGGSGGSGGGSGGGSGGSGSGGSGGSGSGGSGGSG**SNPYARGPN<br>PTAASLEASAGPFTVRSFTVSRPSGYGAGTVYYPTNAGGTVGAIAIVPGY<br>TARQSSIKWWGPRLASHGFVVITIDTNSTLDQPESRSSQQMAALRQVASL<br>NGTSSSPIYGKVDTARMGVMGWSMGGGGSLISAANNPSLKAAAPQAPW<br>HSSTNFSSVTVPTLIFACENDSIAPVNSSALPIYDSMSQNAKQFLEIKGGSH<br>SCANSGNSNQALIGKKGVAWMKRFMDNDTRYSTFACENPNSTAVSDFR<br>TANCSLEHHHHHH
KL4F	AEAAKEAA<br>KEAAKA	14	MMKIVPPKPFFFEAGERAVLLLHGFTGNSADVRMLGRFLESKGYTCHAP<br>IYKGHGVPPEELVHTGPDDWWQDVMNGYEFLKNKGYEKIAVAGLSLG<br>GVFSLKLGYTVPIEGIVTMCAPMYIKSEETMYELVLEYAREYKKREGKSE<br>EQIEQEMEKFKQTPMKTLKALQELKADVRDHLDLIYAPTFVVQARHDE<br>MINPDSANIIYNEIESPVKQIKWYEQSGHVITLDQEKDQLHEDIYAFLESL<br>DW**AEAAKEAAKEAAKA**NPYARGPNPTAASLEASAGPFTVRSFTVSRPS<br>GYGAGTVYYPTNAGGTVGAIAIVPGYTARQSSIKWWGPRLASHGFVVIT<br>IDTNSTLDQPESRSSQQMAALRQVASLNGTSSSPIYGKVDTARMGVMGW<br>SMGGGGSLISAANNPSLKAAAPQAPWHSSTNFSSVTVPTLIFACENDSIAP<br>VNSSALPIYDSMSQNAKQFLEIKGGSHSCANSGNSNQALIGKKGVAWMK<br>RFMDNDTRYSTFACENPNSTAVSDFRTANCSLEHHHHHH

F20KL	GGGSGGSG<br>GGSGGGSG<br>GSGS	20	MNPYARGPNPTAASLEASAGPFTVRSFTVSRPSGYGAGTVYYPTNAGGT<br>VGAIAIVPGYTARQSSIKWWGPRLASHGFVVITIDTNSTLDQPESRSSQQ<br>MAALRQVASLNGTSSSPIYGKVDTARMGVMGWSMGGGGSLISAANNPS<br>LKAAAPQAPWHSSTNFSSVTVPTLIFACENDSIAPVNSSALPIYDSMSQNA<br>KQFLEIKGGSHSCANSGNSNQALIGKKGVAWMKRFMDNDTRYSTFACE<br>NPNSTAVSDFRTANCS**GGGSGGSGGGSGGGSGGSGS**MMKIVPPKPFFF<br>EAGERAVLLLHGFTGNSADVRMLGRFLESKGYTCHAPIYKGHGVPPEEL<br>VHTGPDDWWQDVMNGYEFLKNKGYEKIAVAGLSLGGVFSLKLGYTVPI<br>EGIVTMCAPMYIKSEETMYELVLEYAREYKKREGKSEEQIEQEMEKFKQ<br>TPMKTLKALQELKADVRDHLDLIYAPTFVVQARHDEMINPDSANIIYNEI<br>ESPVKQIKWYEQSGHVITLDQEKDQLHEDIYAFLESLDWLEHHHHHH
F4KL	AEAAKEAA<br>KEAAKA	14	MNPYARGPNPTAASLEASAGPFTVRSFTVSRPSGYGAGTVYYPTNAGGT<br>VGAIAIVPGYTARQSSIKWWGPRLASHGFVVITIDTNSTLDQPESRSSQQ<br>MAALRQVASLNGTSSSPIYGKVDTARMGVMGWSMGGGGSLISAANNPS<br>LKAAAPQAPWHSSTNFSSVTVPTLIFACENDSIAPVNSSALPIYDSMSQNA<br>KQFLEIKGGSHSCANSGNSNQALIGKKGVAWMKRFMDNDTRYSTFACE<br>NPNSTAVSDFRTANCS**AEAAKEAAKEAAKA**MMKIVPPKPFFFEAGERA<br>VLLLHGFTGNSADVRMLGRFLESKGYTCHAPIYKGHGVPPEELVHTGPD<br>DWWQDVMNGYEFLKNKGYEKIAVAGLSLGGVFSLKLGYTVPIEGIVTM<br>CAPMYIKSEETMYELVLEYAREYKKREGKSEEQIEQEMEKFKQTPMKTL<br>KALQELKADVRDHLDLIYAPTFVVQARHDEMINPDSANIIYNEIESPVKQI<br>KWYEQSGHVITLDQEKDQLHEDIYAFLESLDWLEHHHHHH
KLS20F	GGGSGGSG<br>GGSGGGSG<br>GSGS	20	MMKIVPPKPFFFEAGERAVLLLHGFTGNSADVRMLGRFLESKGYTCHAP<br>IYKGHGVPPEELVHTGPDDWWQDVMNGYEFLKNKGYEKIAVAGLSLG<br>GVFSLKLGYTVPIEGIVTMCAPMYIKSEETSYELVLEYAREYKKREGKSE<br>EQIEQEMEKFKQTPMKTLKALQELKADVRDHLDLIYAPTFVVQARHDE<br>MINPDSANIIYNEIESPVKQIKWYEQSGHVITLDQEKDQLHEDIYAFLESL<br>DW**GGGSGGSGGGSGGGSGGSGS**NPYARGPNPTAASLEASAGPFTVRSF<br>TVSRPSGYGAGTVYYPTNAGGTVGAIAIVPGYTARQSSIKWWGPRLASH<br>GFVVITIDTNSTLDQPESRSSQQMAALRQVASLNGTSSSPIYGKVDTARM<br>GVMGWSMGGGGSLISAANNPSLKAAAPQAPWHSSTNFSSVTVPTLIFAC<br>ENDSIAPVNSSALPIYDSMSQNAKQFLEIKGGSHSCANSGNSNQALIGKK<br>GVAWMKRFMDNDTRYSTFACENPNSTAVSDFRTANCSLEHHHHHH
F20KLS	GGGSGGSG<br>GGSGGGSG<br>GSGS	20	MNPYARGPNPTAASLEASAGPFTVRSFTVSRPSGYGAGTVYYPTNAGGT<br>VGAIAIVPGYTARQSSIKWWGPRLASHGFVVITIDTNSTLDQPESRSSQQ<br>MAALRQVASLNGTSSSPIYGKVDTARMGVMGWSMGGGGSLISAANNPS<br>LKAAAPQAPWHSSTNFSSVTVPTLIFACENDSIAPVNSSALPIYDSMSQNA<br>KQFLEIKGGSHSCANSGNSNQALIGKKGVAWMKRFMDNDTRYSTFACE<br>NPNSTAVSDFRTANCS**GGGSGGSGGGSGGGSGGSGS**MMKIVPPKPFFF<br>EAGERAVLLLHGFTGNSADVRMLGRFLESKGYTCHAPIYKGHGVPPEEL<br>VHTGPDDWWQDVMNGYEFLKNKGYEKIAVAGLSLGGVFSLKLGYTVPI<br>EGIVTMCAPMYIKSEETSYELVLEYAREYKKREGKSEEQIEQEMEKFKQT<br>PMKTLKALQELKADVRDHLDLIYAPTFVVQARHDEMINPDSANIIYNEIE<br>SPVKQIKWYEQSGHVITLDQEKDQLHEDIYAFLESLDWLEHHHHHH

* Letters in red bold depict the linker sequences.

Table S17. Properties of PET substrates in this study. The crystallinity of different PET samples was determined by DSC. Crystallinity values correspond to the average of two measurements.

Samples	Description	Particle size (μm)	Crystallinity/%	Source
200 μm Pc-PET	Bottle grade PET powder	100-200	2.9	Coca-Cola
500 μm Pc-PET	Bottle grade PET powder	200-500	5.7	Coca-Cola
1000 μm Pc-PET	Bottle grade PET powder	500-1000	8.7	Coca-Cola
Pc-PET	Original Coke bottle discs	6 mm diameter	26.1	Coca-Cola
200 μm Pc-PET	Bottle grade PET powder after large-scale reaction	100-200	6.1	Coca-Cola

Supplementary Figures

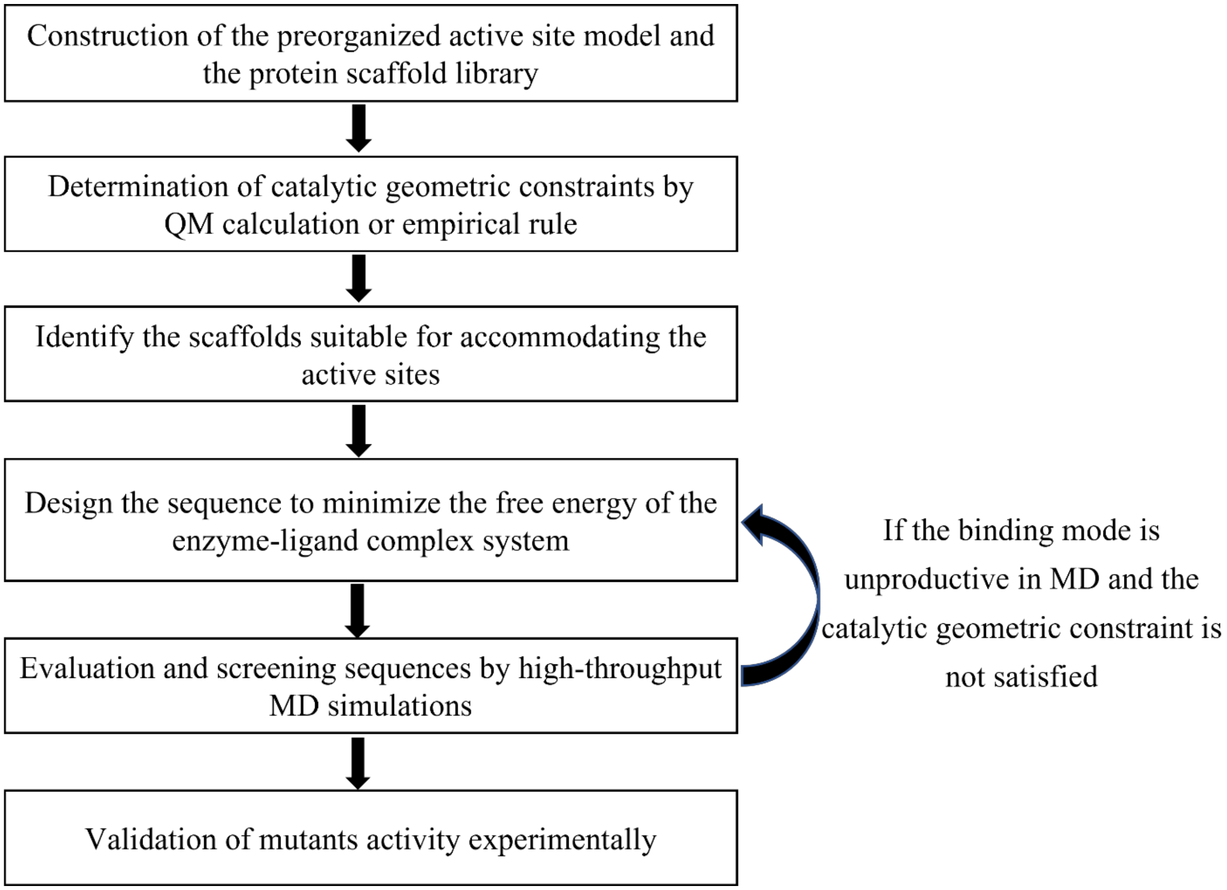

Fig. S1. Computational enzyme design framework in PRODA.

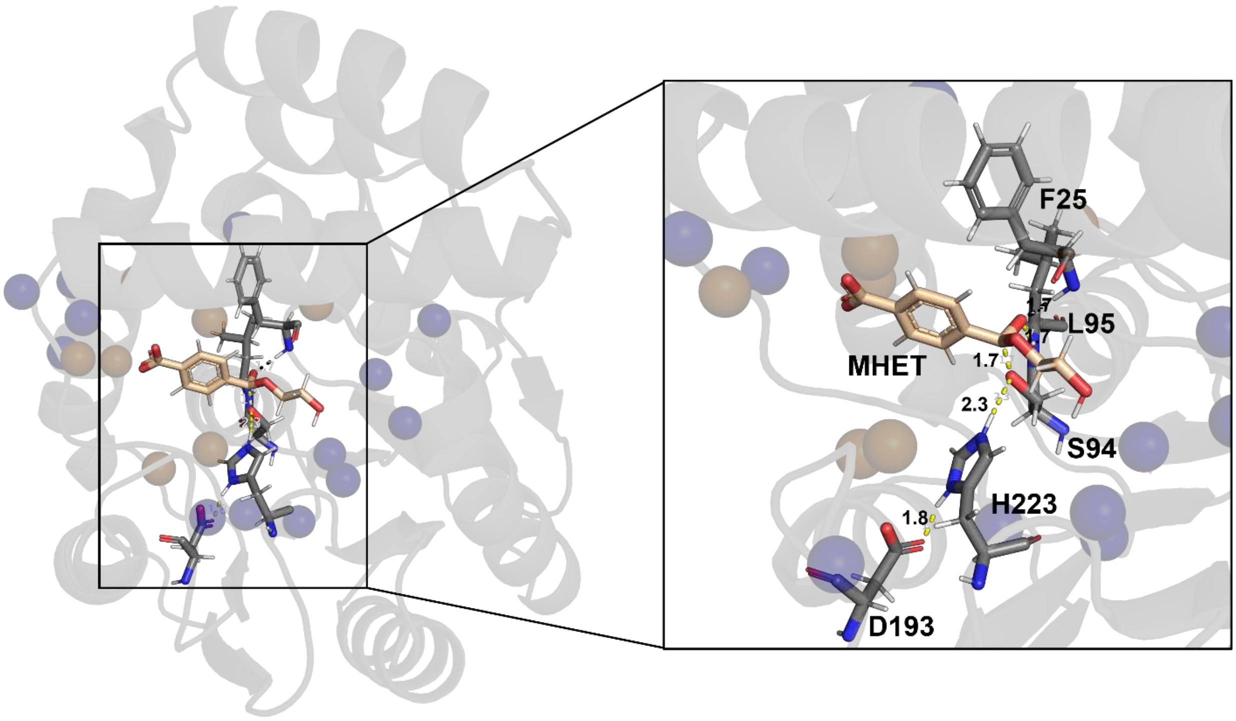

Fig. S2. Locations of the design sites in wild type Est30. The protein structure is shown as cartoon and colored in white. TS is shown in stick model and colored in wheat. Catalytic sites are shown in stick model and colored in black. Sequence selection positions are shown as orange balls, and the side-chain conformation optimization residues are shown as blue balls. The right graph is a close-up of TS. The hydrogen bonds are shown in yellow dashed lines, around which the numbers indicate the distances (Å).

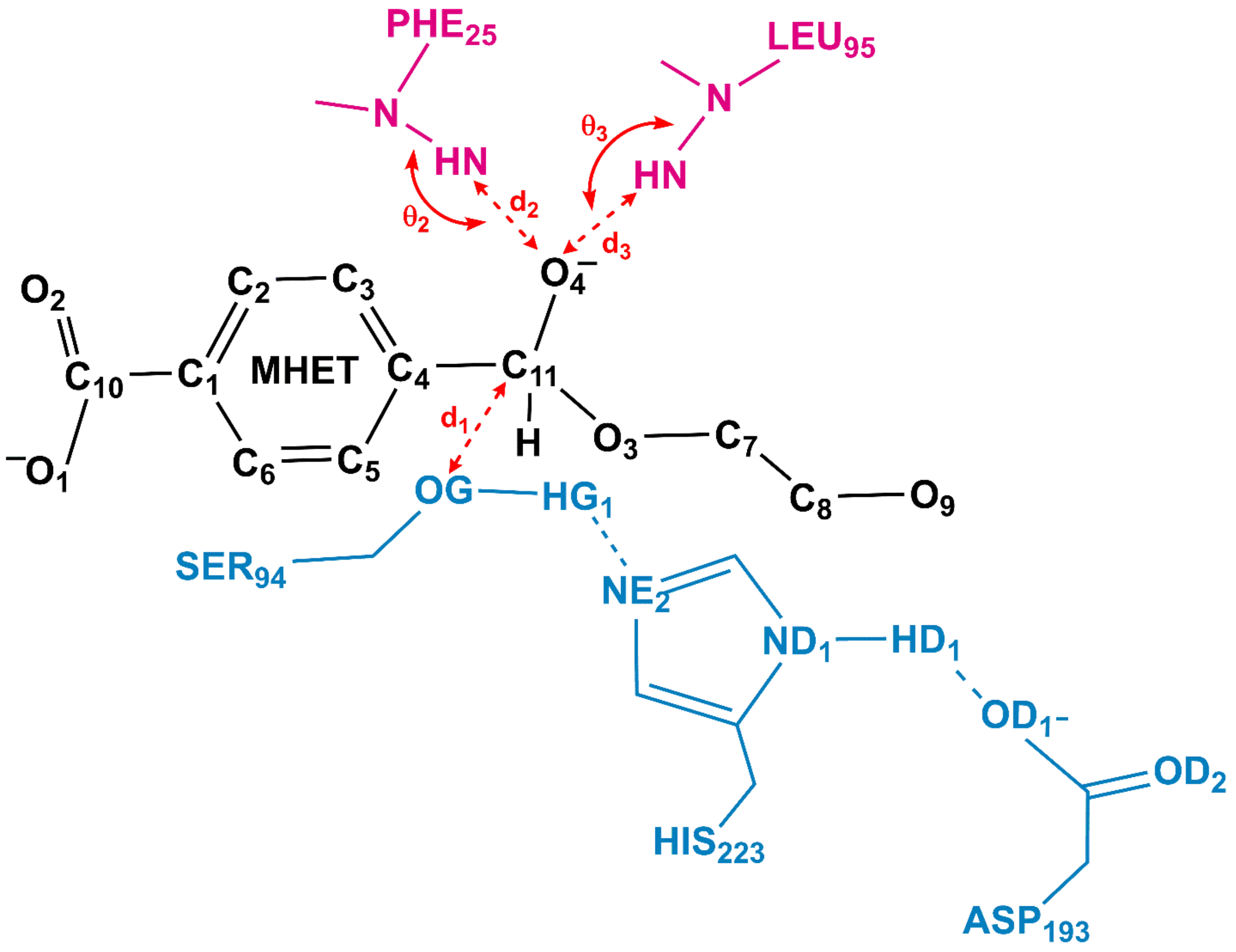

Fig. S3. Representation of the catalytic features for MHET hydrolysis by Est30. The enzyme-TS complex structure is defined to be the catalytic productive conformation in MD simulation when the geometric criteria are simultaneously met (d_1 < 3.0 Å, d_2 < 3.5 Å, θ_2 > 120°, d_3 < 3.5 Å, θ_3 > 120°).

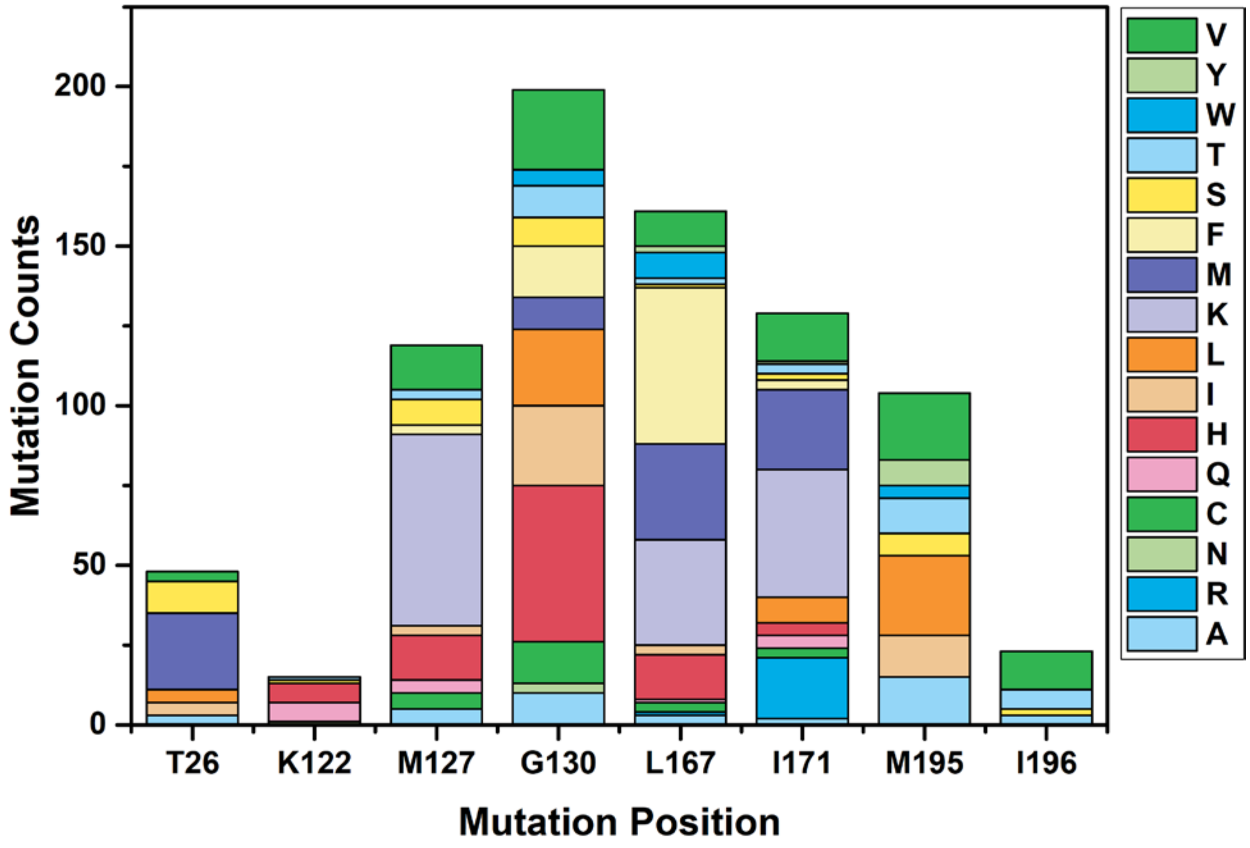

Mutation Counts
0
50
100
150
200
T26
K122
M127
G130
L167
I171
M195
I196
Mutation Position
V
Y
W
T
S
F
M
K
L
I
H
Q
C
N
R
A

(a)

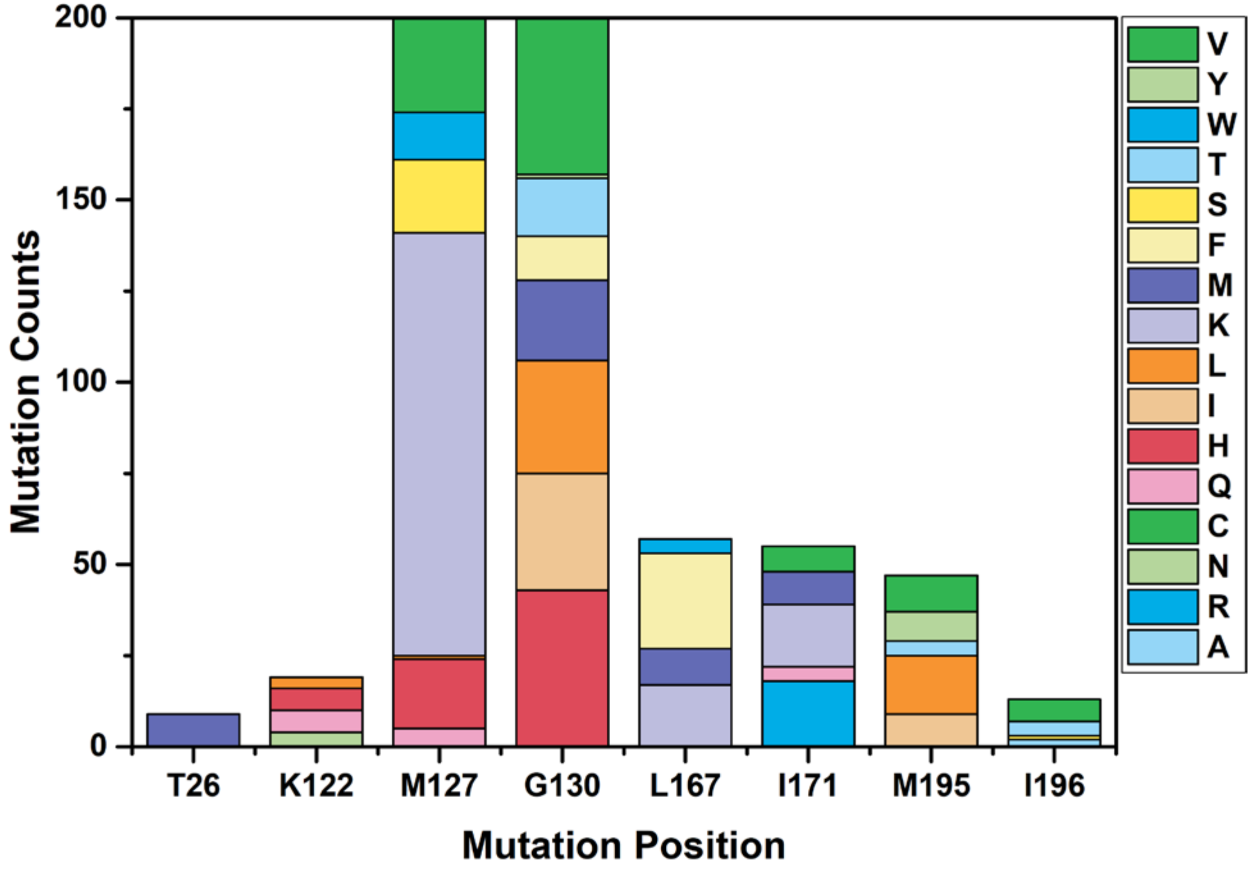

(b)

Fig. S4. Residue mutation counts at design positions among all calculated sequences. (a) double-mutations, and (b) triple-mutations. Mutation counts refer to the number of each amino acid type selected at the particular design position among all calculated sequences.

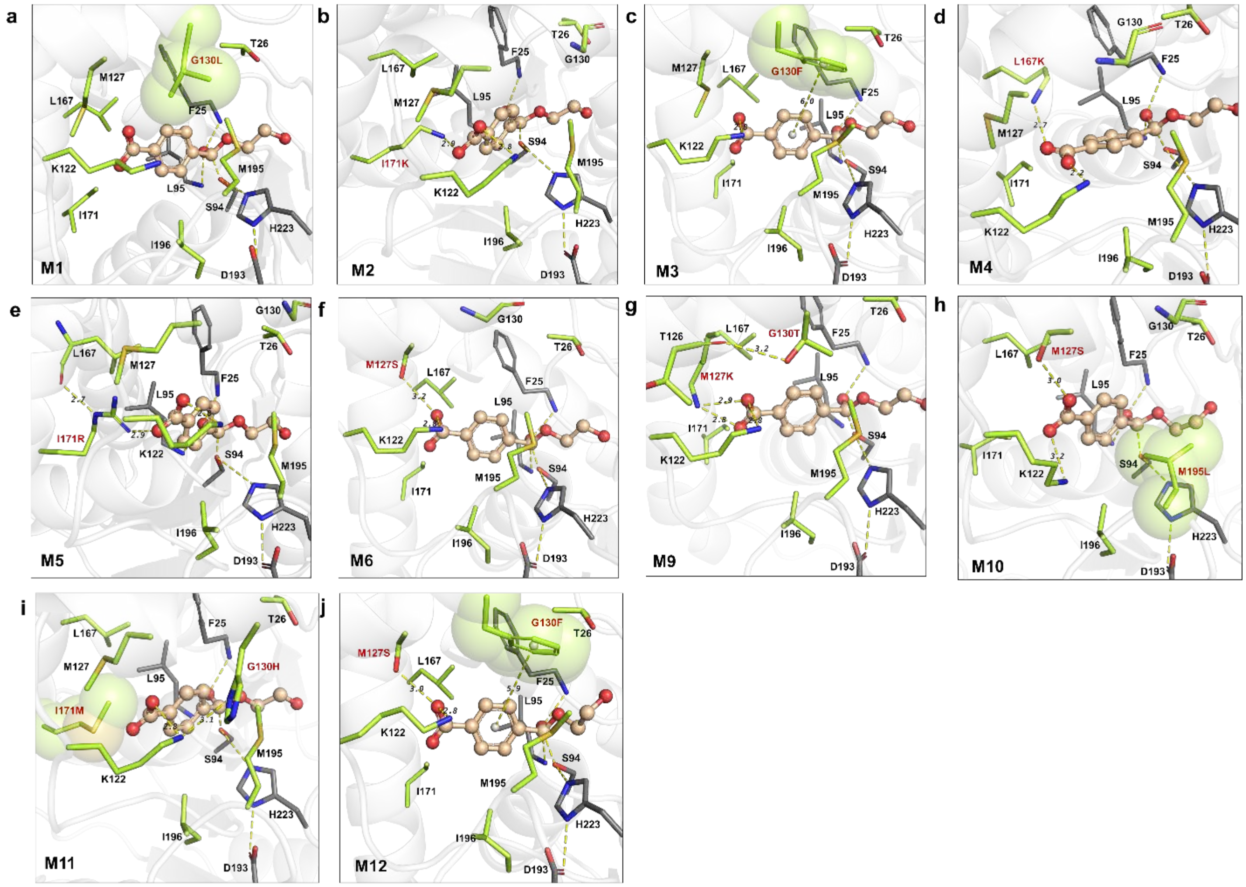

Fig. S5. The computed binding geometries of MHET in scaffold 1TQH and the designed variants. (a) M1(G130L); (b) M2(I171K); (c) M3(G130F); (d) M4(L167K); (e) M5(I171R); (f) M6(M127S); (g) M9(M127K/G130T) (h) M10(M195L/M127S); (i) M11(I171M/G130H); (J) M12(M127S/G130F). The protein structures are shown as cartoon and colored in white. The TS of MHET is shown in ball-and-stick model and colored wheat, while residues are shown in stick model. The sequence selection residues are colored in lemon, while the catalytic triad and oxygen anion hole residues are colored in gray. The hydrogen bonds are shown in yellow dashed lines, around which the numbers indicate the distances (Å).

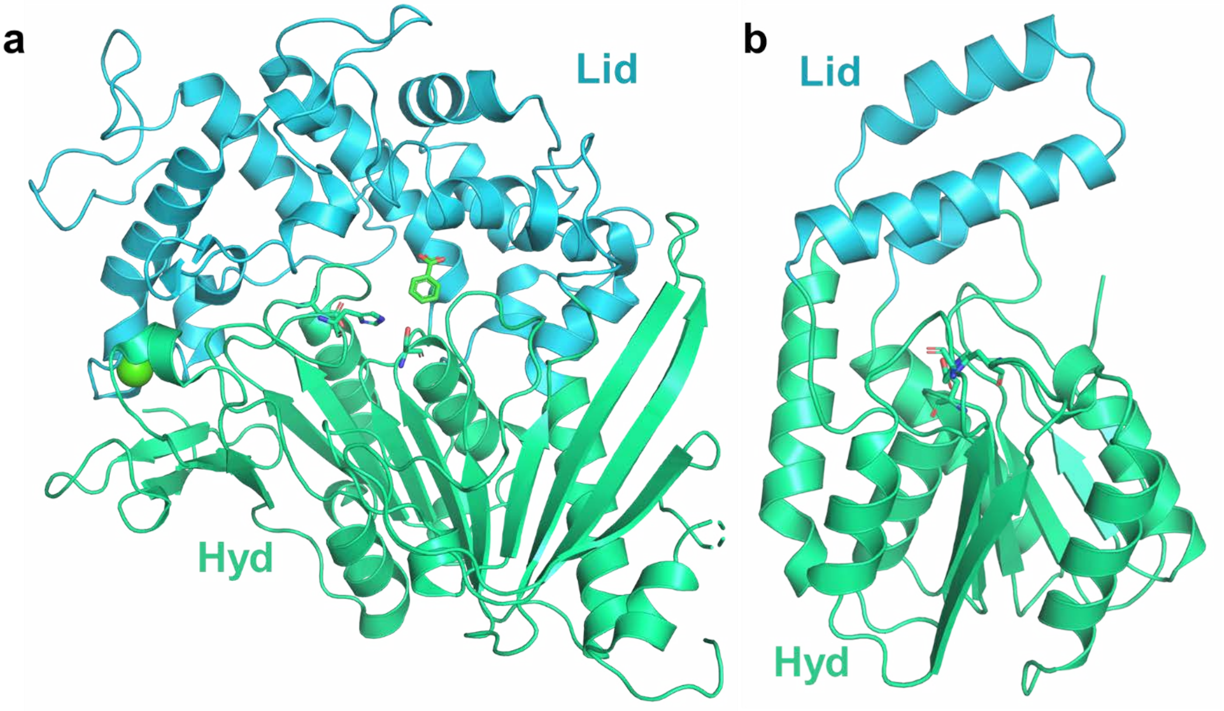

Fig. S6. Comparison of the structural domains of *Is*MHETase and Est30. (a) the crystal structure of *Is*MHETase (PDB Code: 6QZ3), and (b) the crystal structure of Est30 (PDB Code: 1TQH). The protein structures are shown as cartoon. The hydrolase domains (Hyd) are colored in green and the lid domains (Lid) in blue. The catalytic triad residues are shown as sticks.

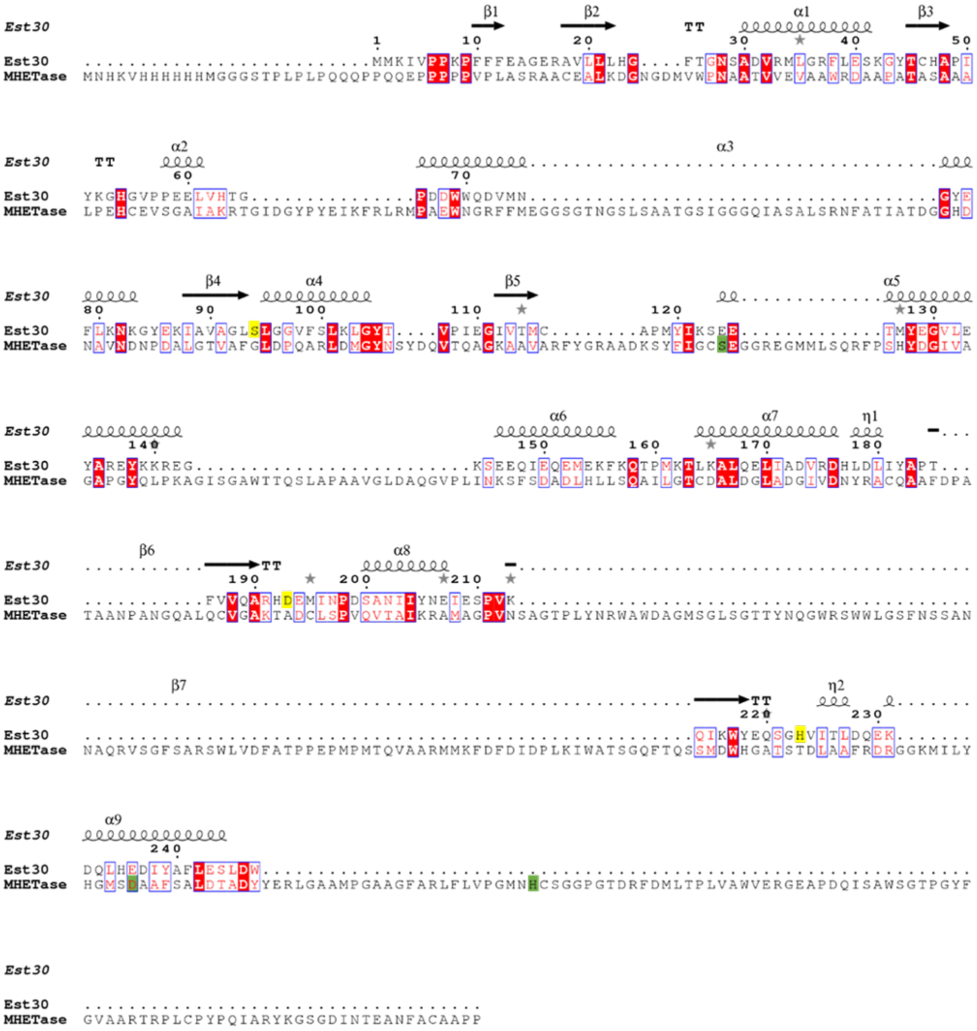

Fig. S7. Sequence alignment of Est30 (PDB Code: 1TQH) and *Is*MHETase (PDB Code: 6QZ3). The sequence analysis is carried out with Clustal Omega[21] and aligned with ESPript[22]. Squares shaded in red refer to the same region, and residues in red text are moderately conserved. The cartoon above the alignment refers to the secondary structure of Est30. The helix refers to the α-helix, the arrow refers to the β-strand, and the "T" refers to the turn. A yellow and green box indicates the catalytic triad residues of Est30 and *Is*MHETase, respectively.

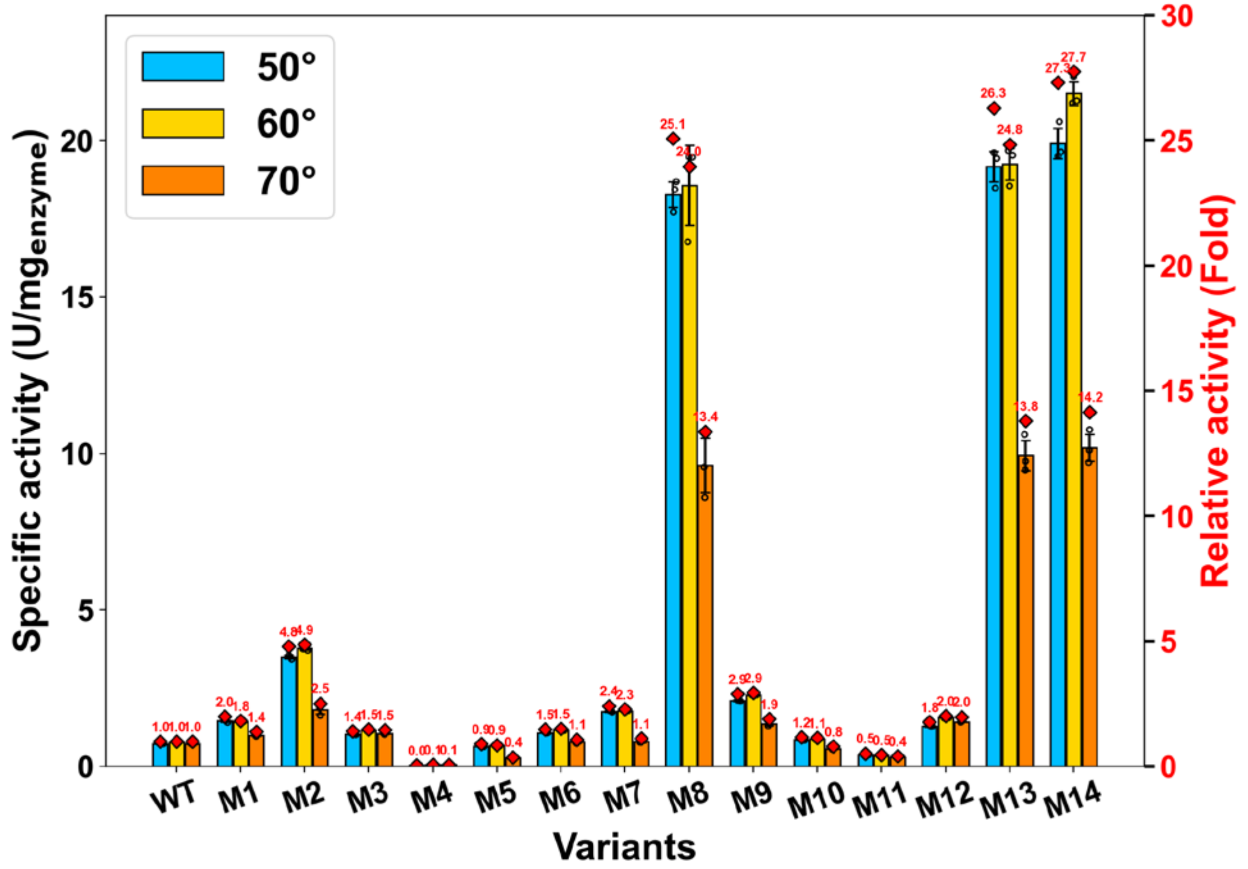

Fig. S8. MHET hydrolytic activities of wild type Est30 and the designed variants at 50 °C, 60 °C and 70 °C, respectively. The reactions were performed in triplicate over 10 min using 0.2-2 μM purified enzyme and 5mM MHET in 50 mM sodium phosphate, pH 7.5.

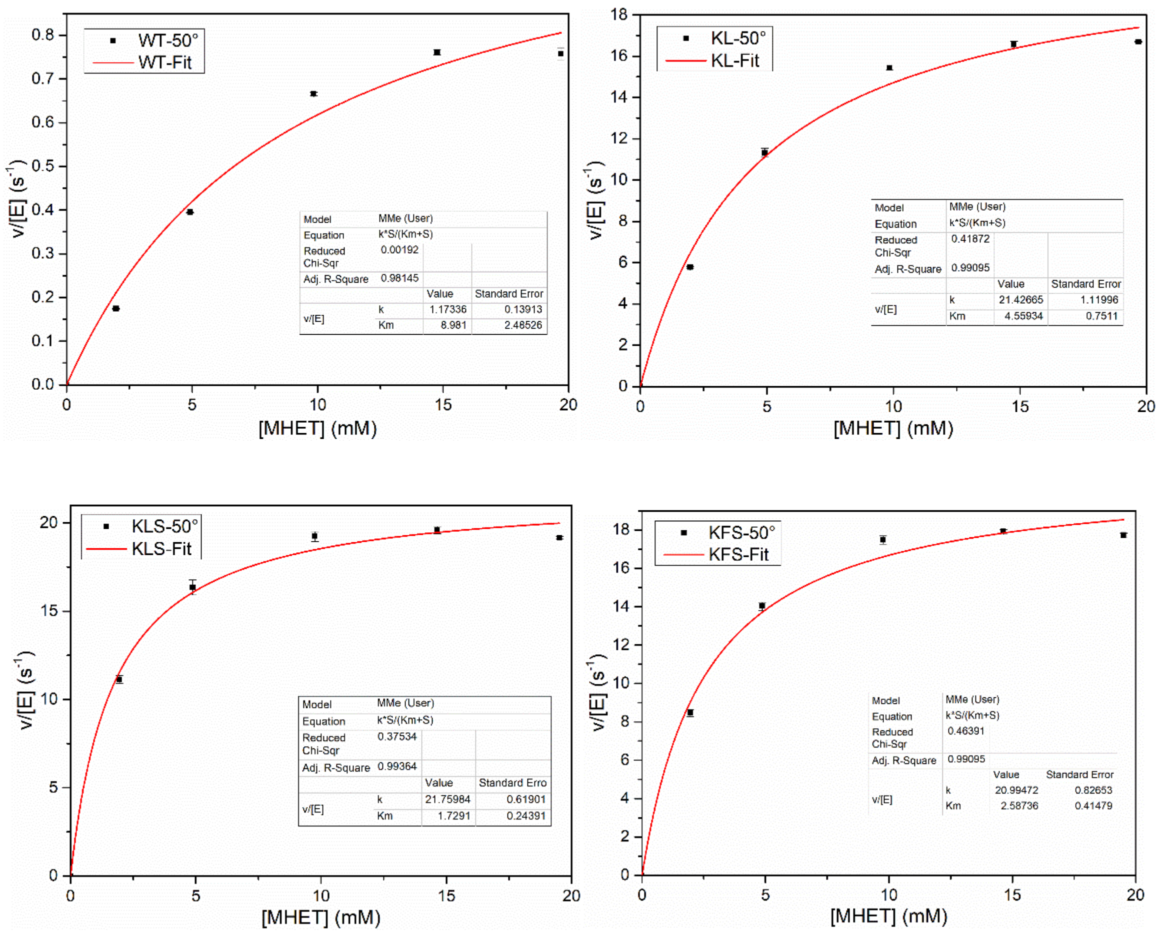

Fig. S9. Michaelis-Menten plots of the wild type Est30 and the designed variants, M8(I171K/G130L, KL), M13(I171K/G130F/M127S, KFS), M14(I171K/G130L/M127S, KLS). The reactions were performed in triplicate at 50 ^{o}C.

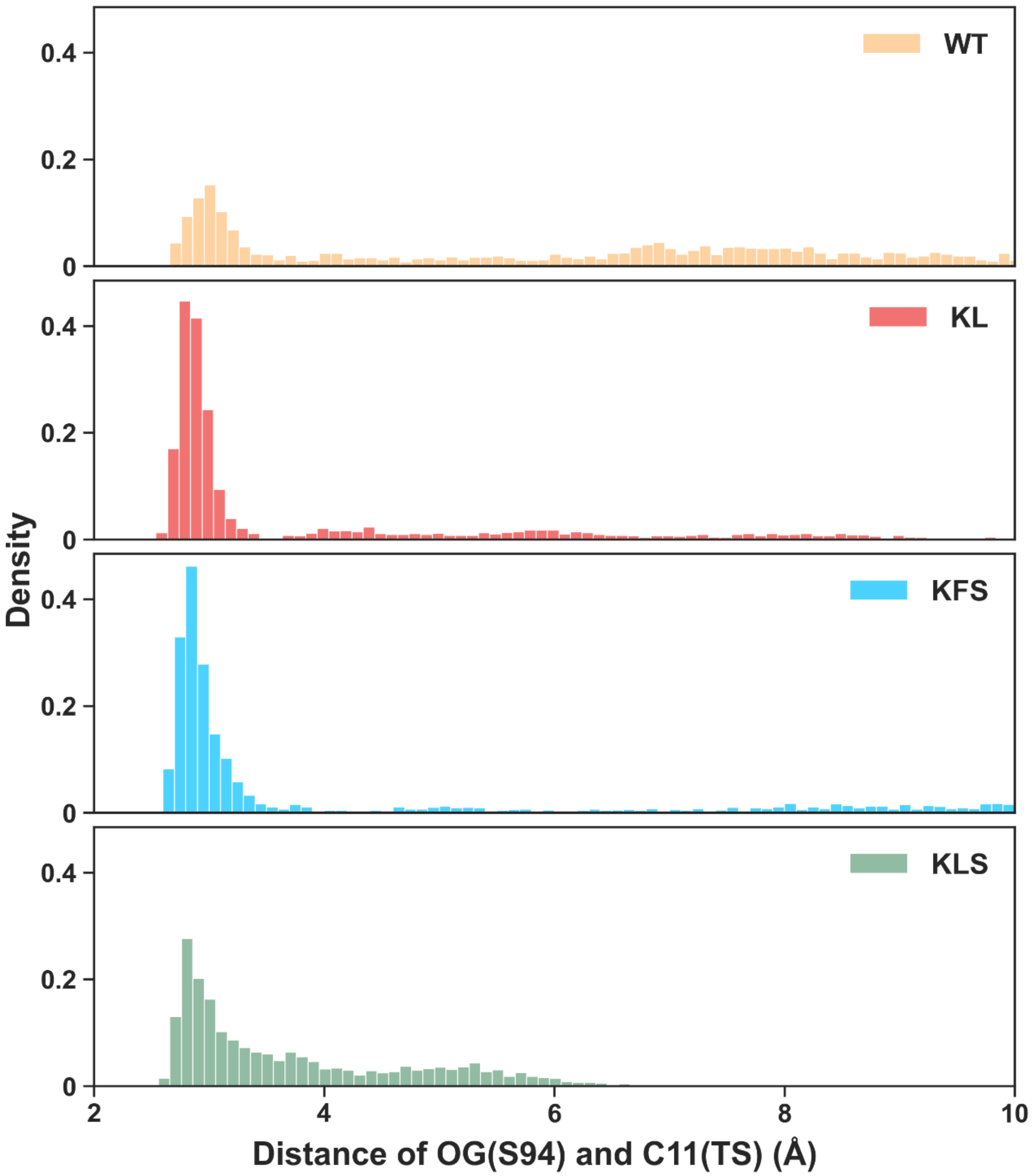

Fig. S10. Comparison of the probability distribution of the nucleophilic attack distance in 5×5 ns MD simulations for the wild type Est30 and three highly active variants M8(I171K/G130L, KL), M13(I171K/G130F/M127S, KFS), M14(I171K/G130L/M127S, KLS).

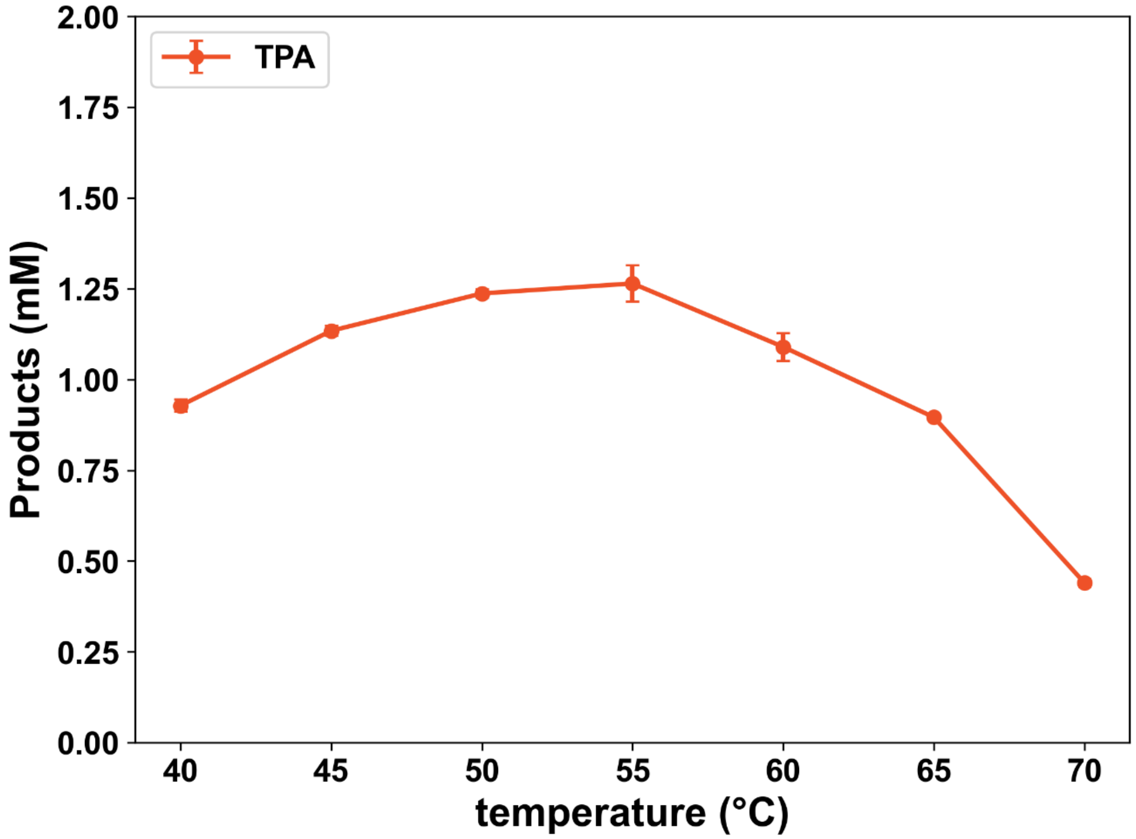

Fig. S11. The hydrolytic activity of KL-MHETase towards MHET at various temperatures. The reactions were performed in triplicate over 10 min using 0.2 μM purified enzyme and 4.8 mM MHET in 50 mM sodium phosphate, pH 7.5.

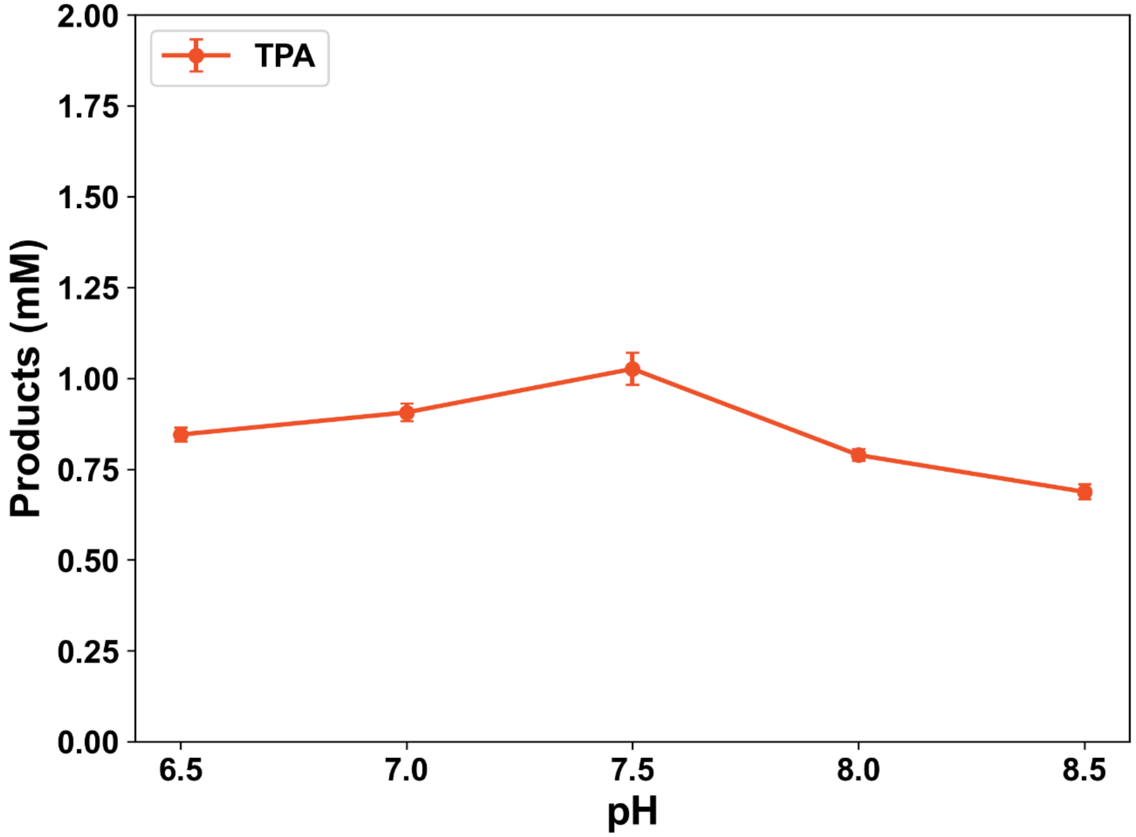

Fig. S12. The hydrolytic activity of KL-MHETase towards MHET at various pH values. The reactions were performed in triplicate over 10 min using 0.2 μM purified enzyme and 4.8 mM MHET in 50 mM sodium phosphate, at 50 °C.

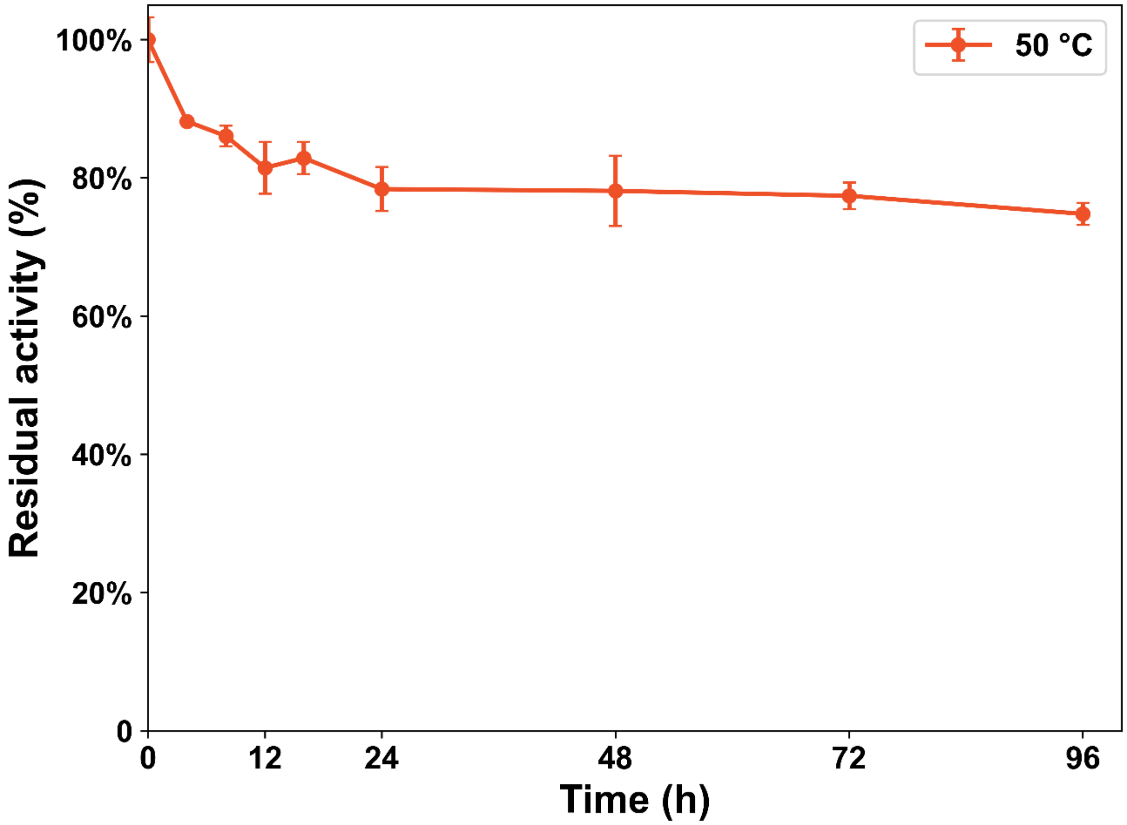

Fig. S13. Thermostability characterization of KL-MHETase. The thermostability of KL-MHETase was determined by measuring the residual activity towards MHET hydrolysis after heating without substrate at 50 °C and pH 7.5 for 0, 4, 8, 12, 16, 24, 48, 72 and 96 h. The initial activity of KL-MHETase without incubation was specified as 100 %. The reactions were performed in triplicate over 20 min using 0.2 μM purified enzyme and 2.0 mM MHET in 50 mM sodium phosphate, at 50 °C.

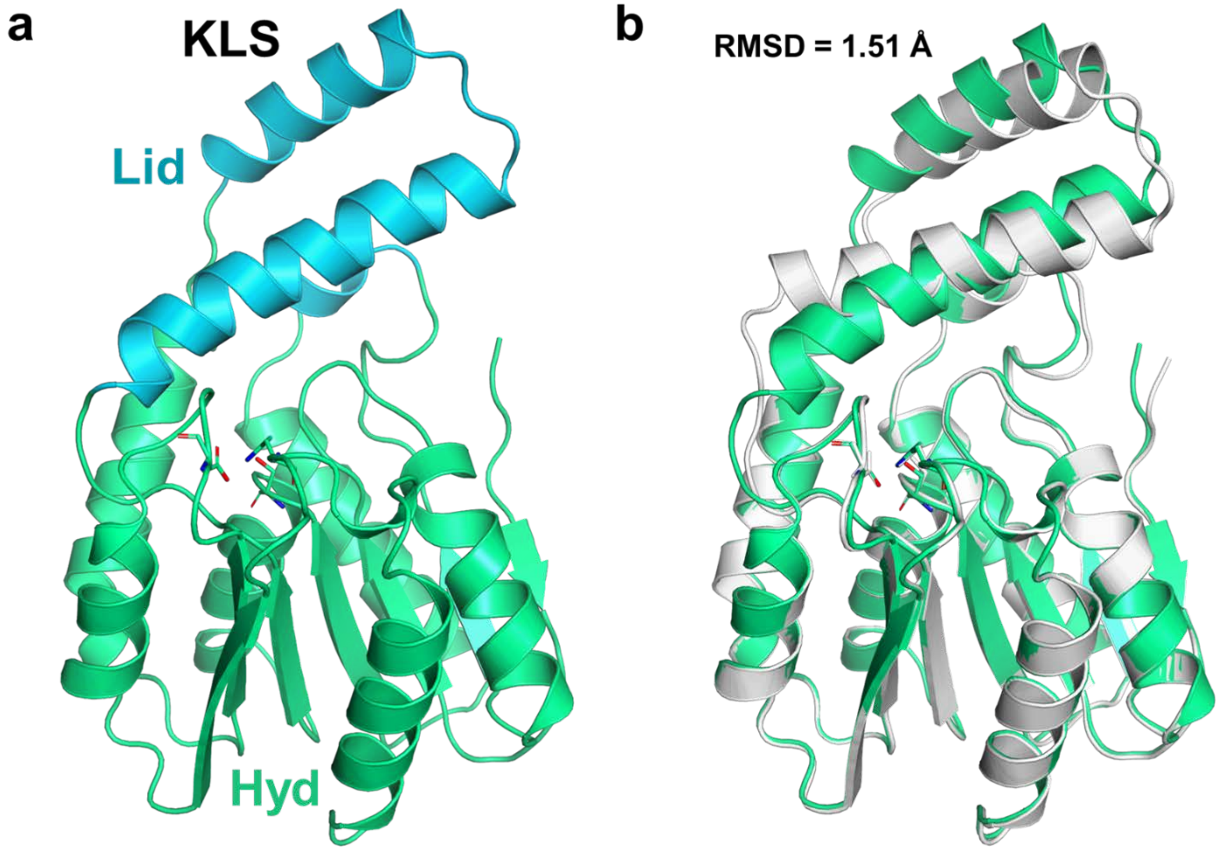

Fig. S14. The crystal structure of KLS-MHETase (PDB Code: 8ILT) and the structure alignment with Est30 (PDB Code:1TQH). (A) The crystallographic structure of KLS-MHETase contains two subdomains: hydrolase domain (Hyd, green) and lid domain (Lid, blue). (B) The structural superimposition of Est30 (white) and the variant KLS (green) indicates a larger change in lid domain. The main-chain RMSD value is 1.51 Å. The protein structures are shown as colored cartoon. The catalytic triad residues S94, H223, and D193 are shown as sticks.

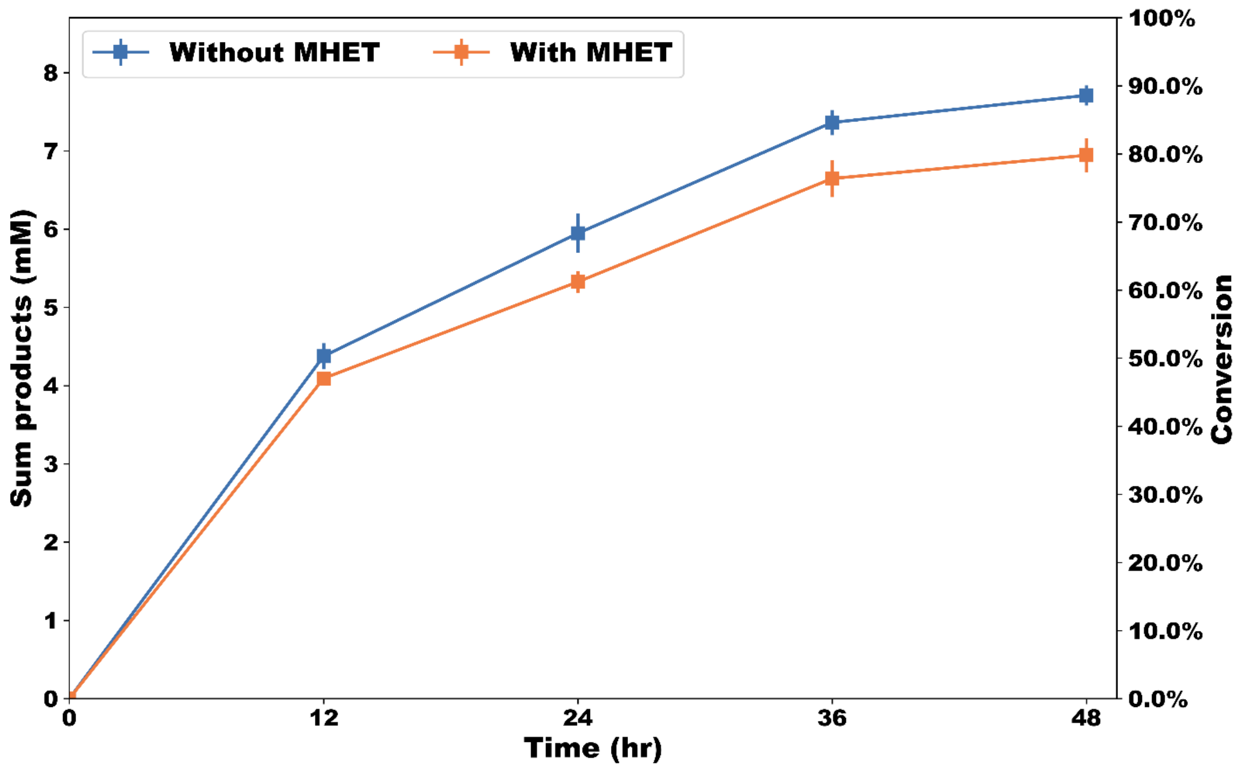

Fig. S15. Inhibition test of MHET on PET degradation by FAST-PETase. Product inhibition was tested through the addition of 2 mM MHET to the reaction mixture along with post-consumer bottle grade PET powder (100-200 um). Reaction progress was monitored by the sum of aromatic products (TPA, MHET and BHET) produced by the degradation of 1.66 g·L-1 post-consumer bottle grade PET powder (100-200 μm) using FAST-PETase (0.35 μM) over 48 hours at 50 °C. Reactions were performed in pH 8.0, 100 mM phosphate buffer, in triplicate. The PET conversion was shown on right ordinate.

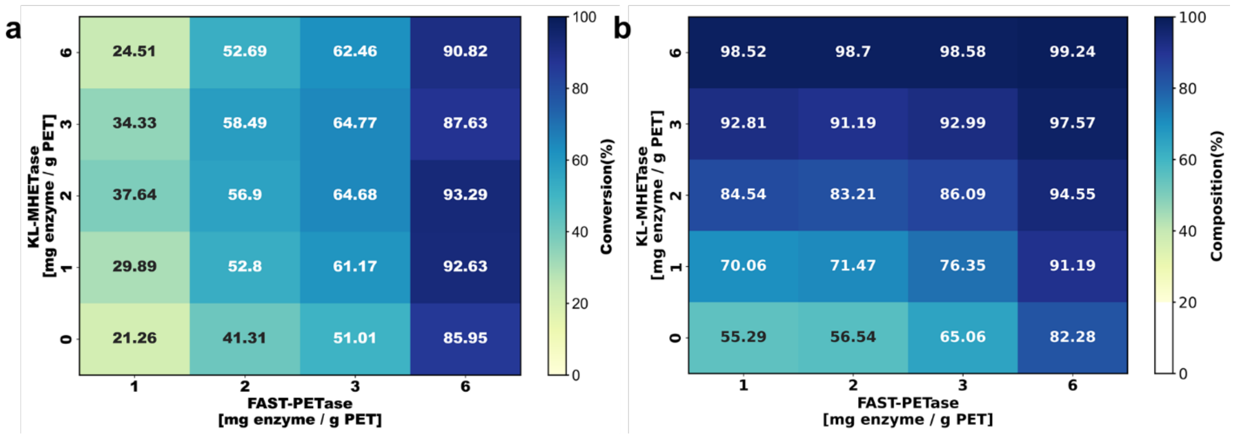

Fig. S16. Synergy of FAST-PETase and KL-MHETase for PET degradation. (a) The conversion heatmap of synergistic PET degradation by FAST-PETase and KL-MHETase over 48 h at 50 ℃. (b) The composition heatmap of synergistic PET degradation by FAST-PETase and KL-MHETase over 48 h at 50 ℃. Reactions were carried out in triplicate on post-consumer bottle grade PET powder (100-200 μm) at pH 8.0 in 100 mM sodium phosphate buffer. Abscissa: FAST-PETase loading (mg/g PET), Ordinate: KL-MHETase loading (mg/g PET).

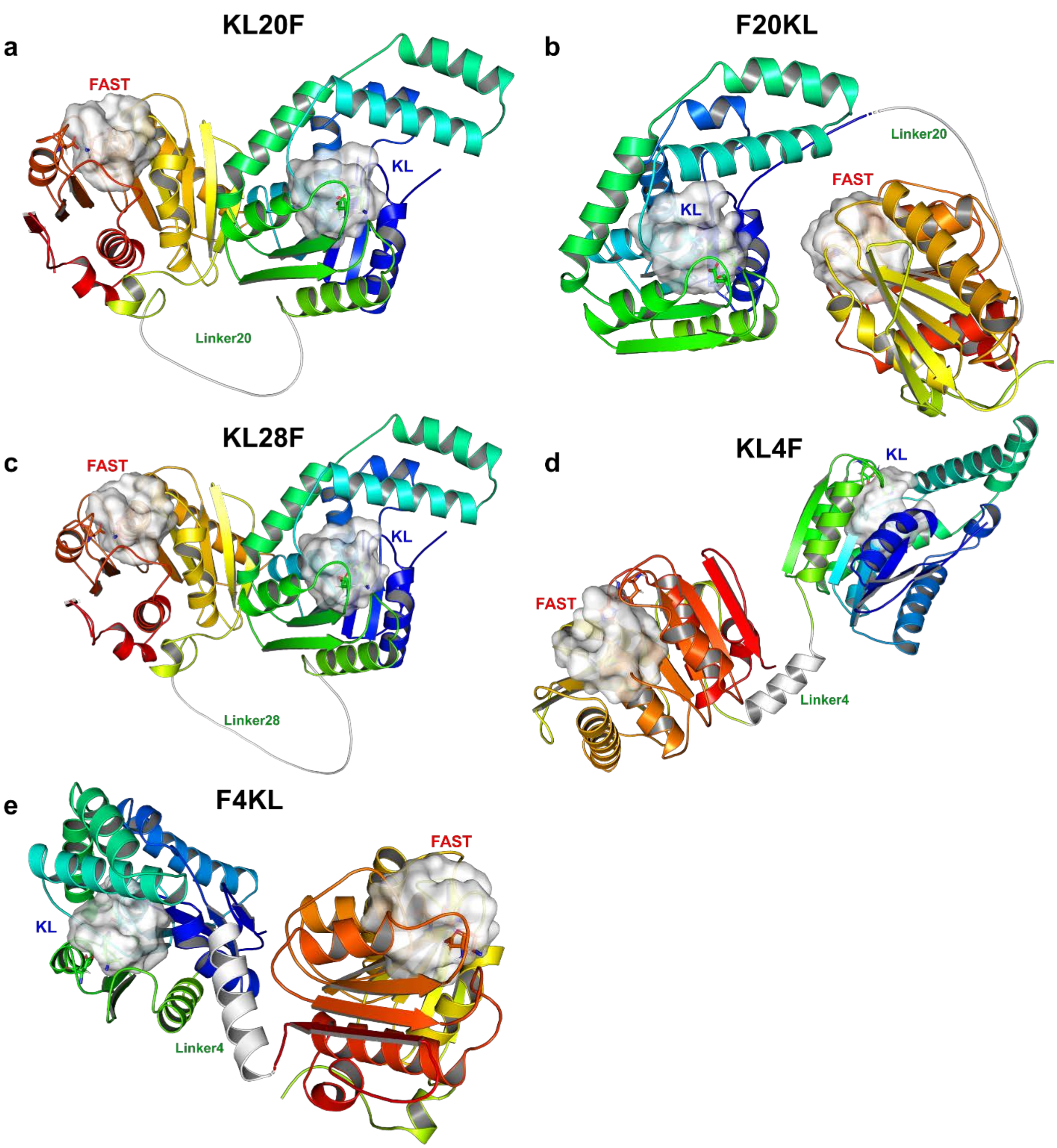

Fig. S17. The predicted structures of constructed fusion enzymes. The protein domains are shown as colored cartoon and linkers shown as white. The active site residues are shown as white surface to indicate the enzyme binding pockets.

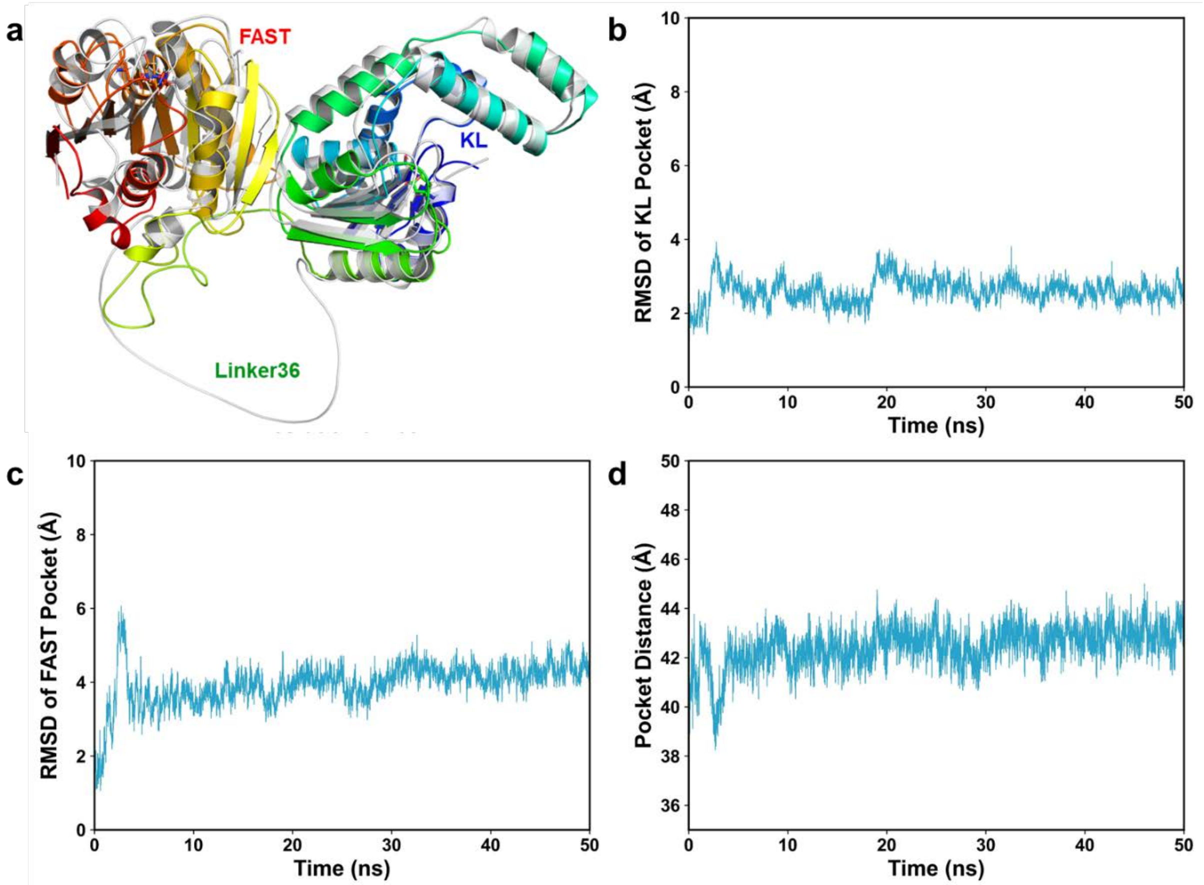

Fig. S18. Conformation change of KL36F in 50 ns MD simulation. (a) Structural overlay of KL36F conformations in the first and last snapshots during a 50 ns MD simulation. The protein domains are shown as cartoon and the residues of the catalytic triads are shown in licorice. The initial conformation of KL36F is shown in white while the snapshot at 50 ns is shown colored. (b-c) The RMSD values for enzyme binding pockets of KL-MHETase (b) and FAST-PETase (c). (d) The distances between two CA atoms of the serine in the catalytic triads of the two enzymes. The values are the average of those in two independent MD simulations.

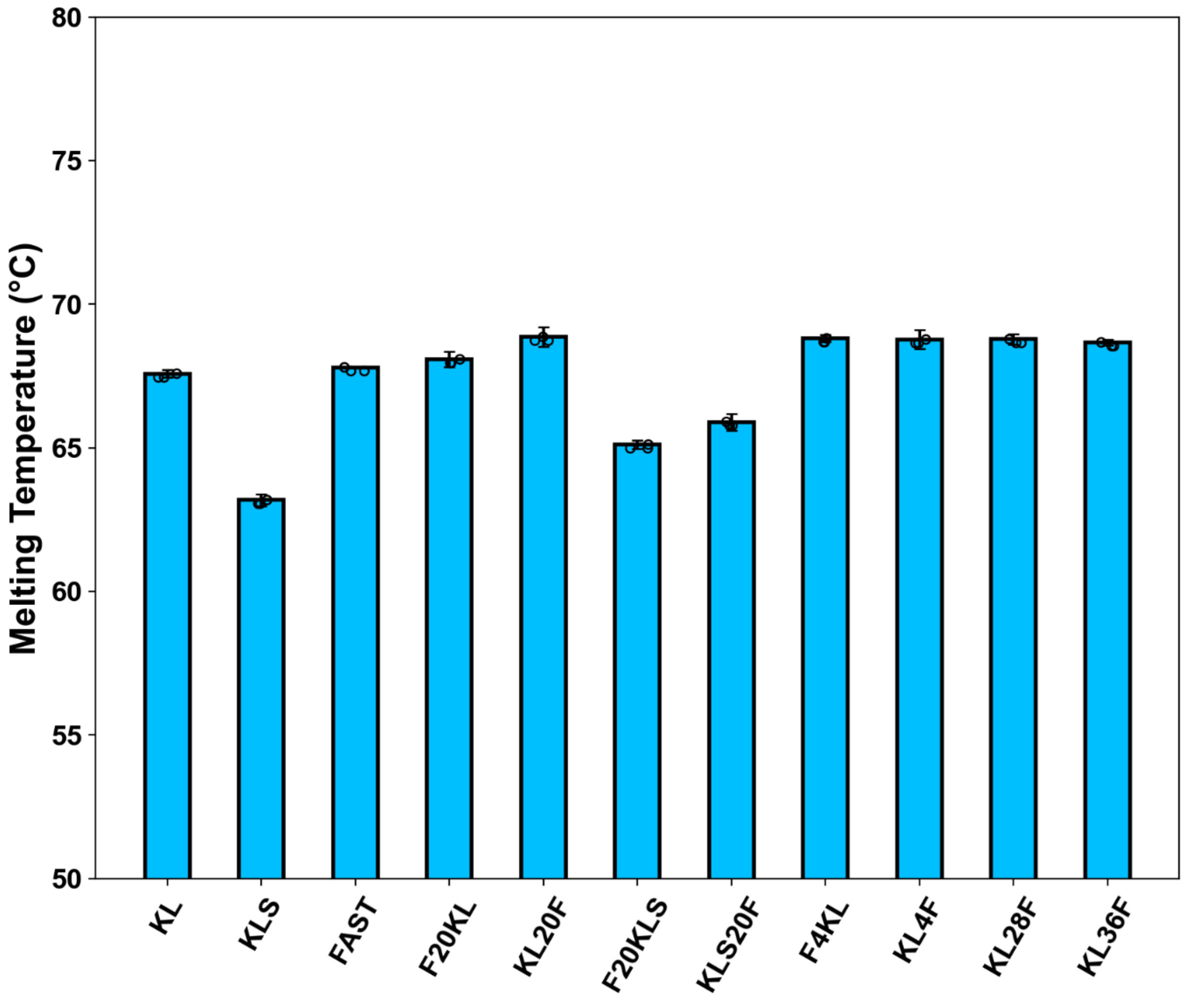

Fig. S19. Comparison of T_m values of KL-MHETase, KLS-MHETase, FAST-PETase and the constructed fusion enzymes. T_m was determined by differential scanning calorimetry from 30 to 120 ℃ and the assay conditions are 50 mM sodium phosphate, pH 7.5 and 0.5–1.0 mg·mL^{-1} enzyme. T_m values correspond to the average of two measurements.

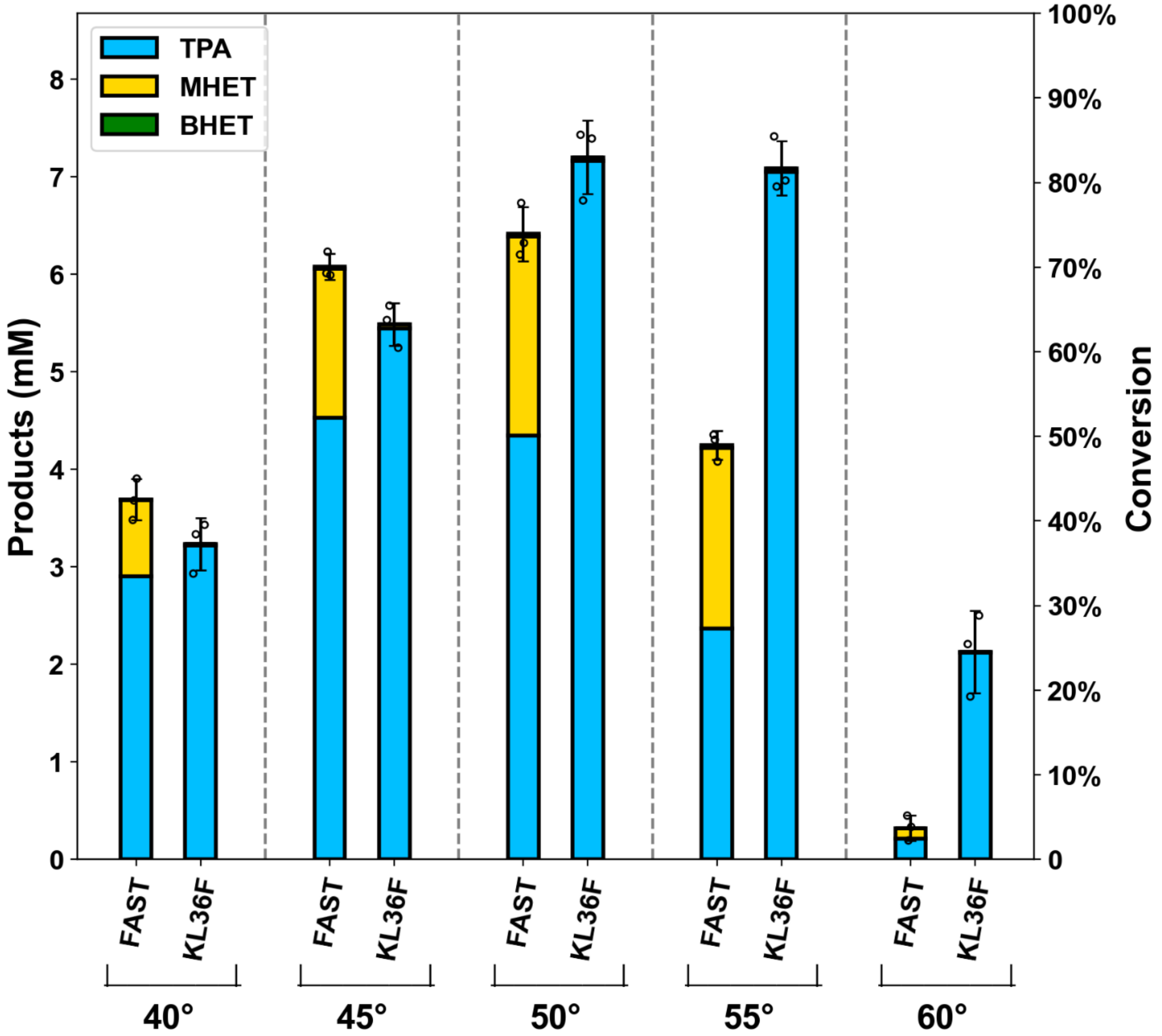

Fig. S20. Comparison of PET degradation activity of FAST-PETase and KL36F at different temperatures. Bar chart shows the concentrations of TPA, MHET and BHET produced by the degradation of 1.66 g·L^{-1} Pc-PET powder (100-200 μm) using FAST-PETase and KL36F over 24 h at different temperatures (40 °C, 45 °C, 50 °C, 55 °C, 60 °C). The concentrations of the enzymes were all 0.35 μM. Reactions were performed in pH 8.0, 100 mM phosphate buffer, in triplicate. The PET conversion was shown on right ordinate.

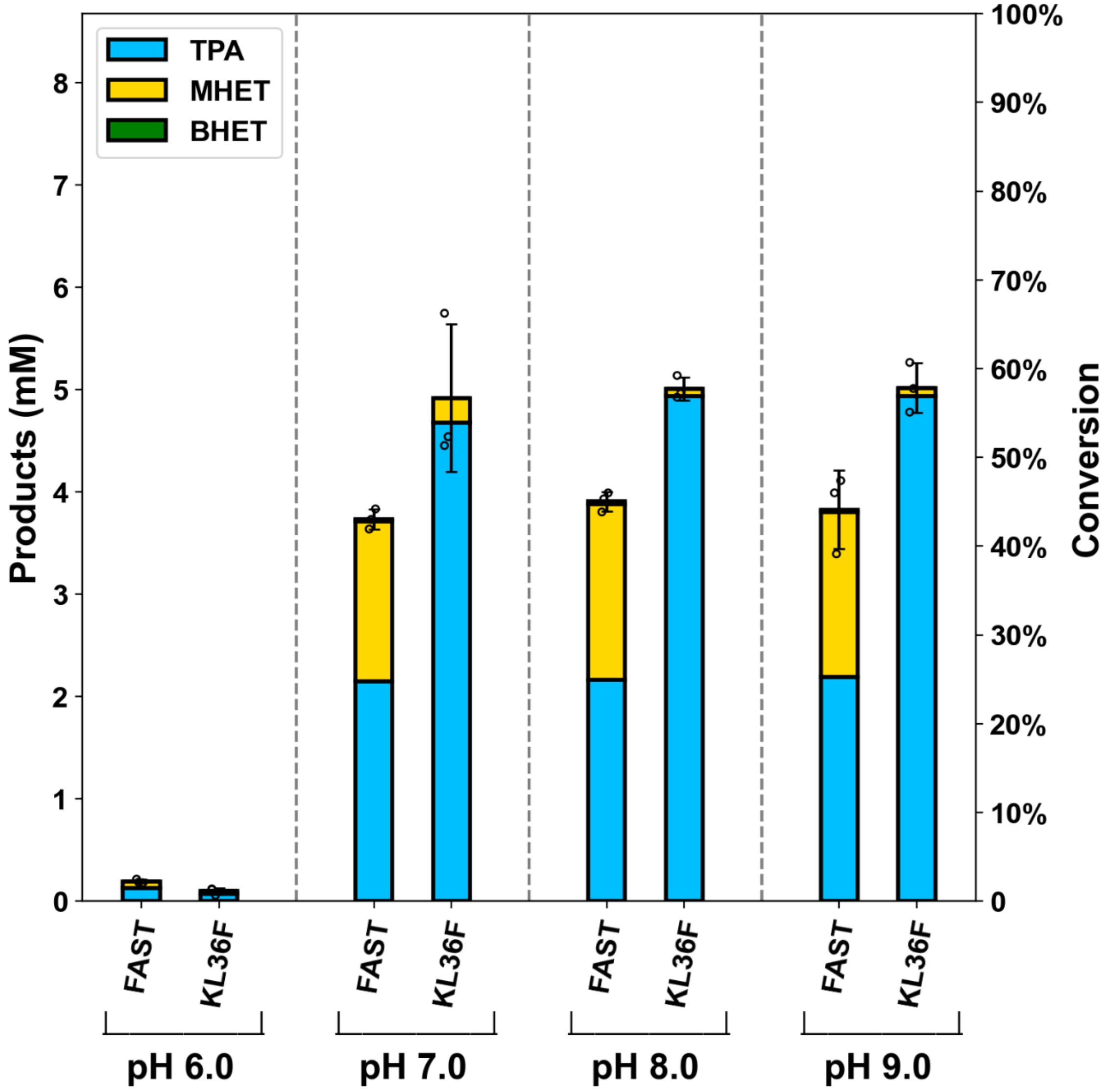

Fig. S21. Comparison of the PET degradation activities of FAST-PETase and KL36F across a range of pH (6.0-9.0). Bar chart shows the concentrations of TPA, MHET and BHET produced by the degradation of 1.66 g·L^{-1} Pc-PET powder (100-200 μm) using FAST-PETase and KL36F at 50 °C over 12 h. The concentrations of the enzymes were all 0.35 μM. Reactions were performed in 100 mM phosphate buffer across a range of pH (6.0-9.0), in triplicate. The PET conversion was shown on right ordinate.

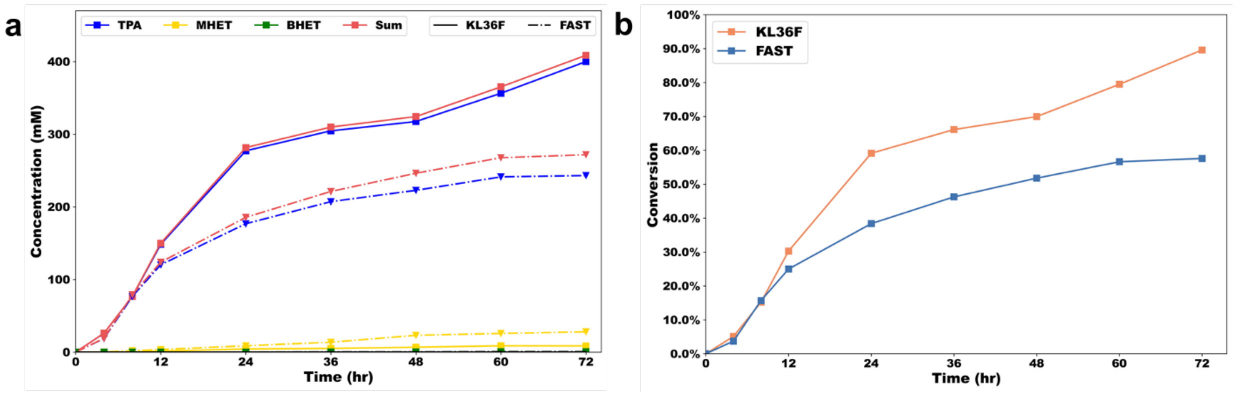

Fig. S22. Time course of PET degradation over 72 h for FAST-PETase and KL36F under high solid loadings in bioreactor (50 mL). (a) Time course of aromatic products released. (b) Time course of degradation conversion. Reactions were performed in 100 mM sodium phosphate using 10% Pc-PET (100-200 μm) substrate loading (100 $g \cdot L^{-1}$) and 12 mg FAST/g PET (40.5 μM) or 24 mg KL36F/g PET (40.5 μM) at 50°C with continuous pH control around 8.0.

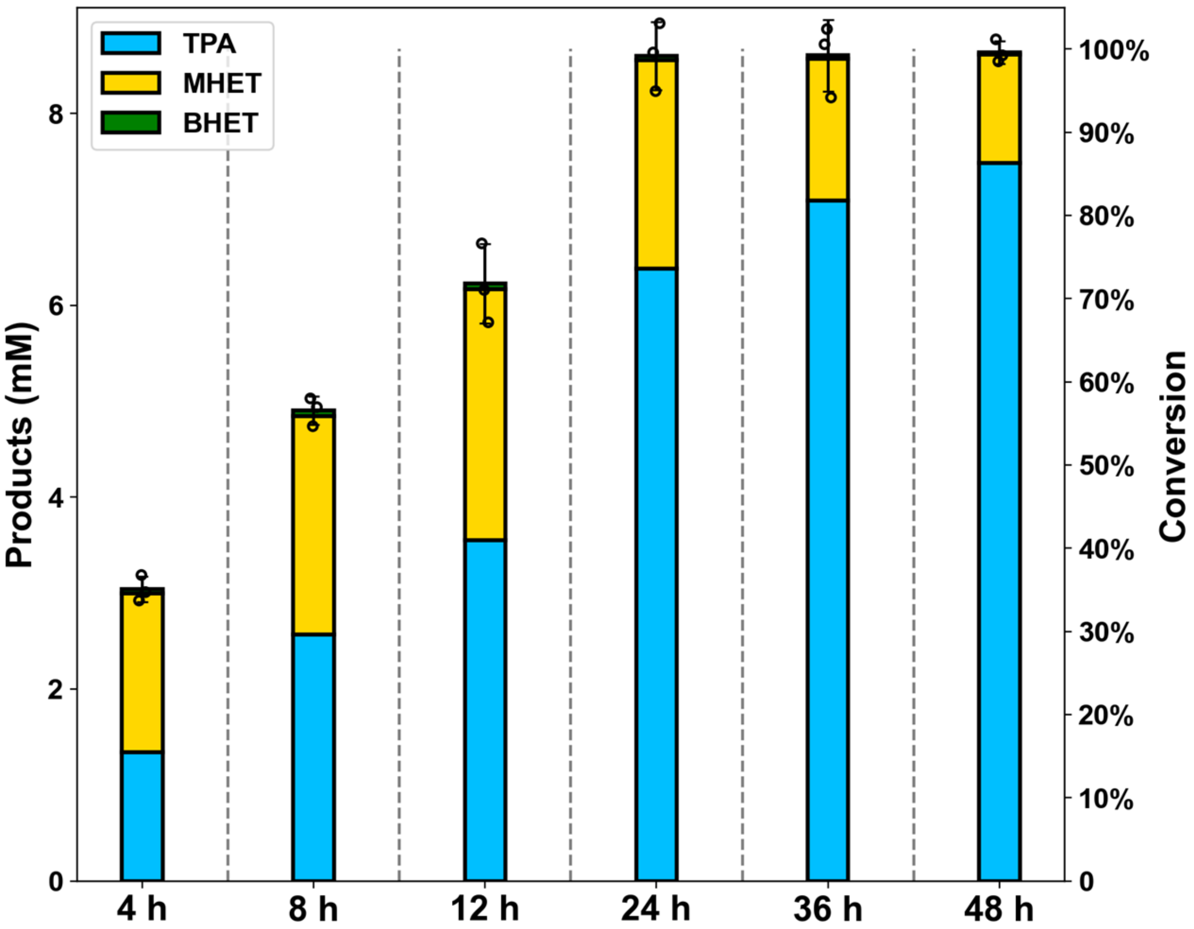

Fig. S23. Time-course of PET degradation activity of LCC-ICCG over 48 h. Bar chart shows the concentrations of TPA, MHET and BHET produced by the degradation of 1.66 g·L^{-1} Pc-PET powder (200-500 μm) using LCC-ICCG over 48 h at 70°C. The concentration of the enzyme was 0.17 μM. Reactions were performed in pH 8.0, 100 mM phosphate buffer, in triplicate. The PET conversion was shown on right ordinate.

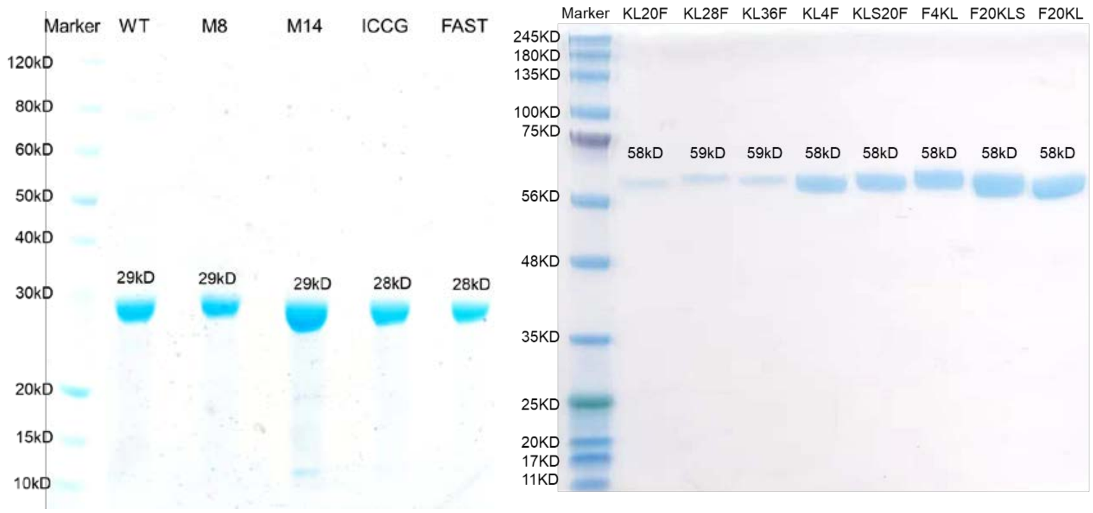

Fig. S24. SDS-PAGE gel of purified wild-type Est30, the designed variants M8(I171K/G130L, KL-MHETase), M14(I171K/G130L/M127S, KLS-MHETase), LCC-ICCG, FAST-PETase and the constructed fusion enzymes.

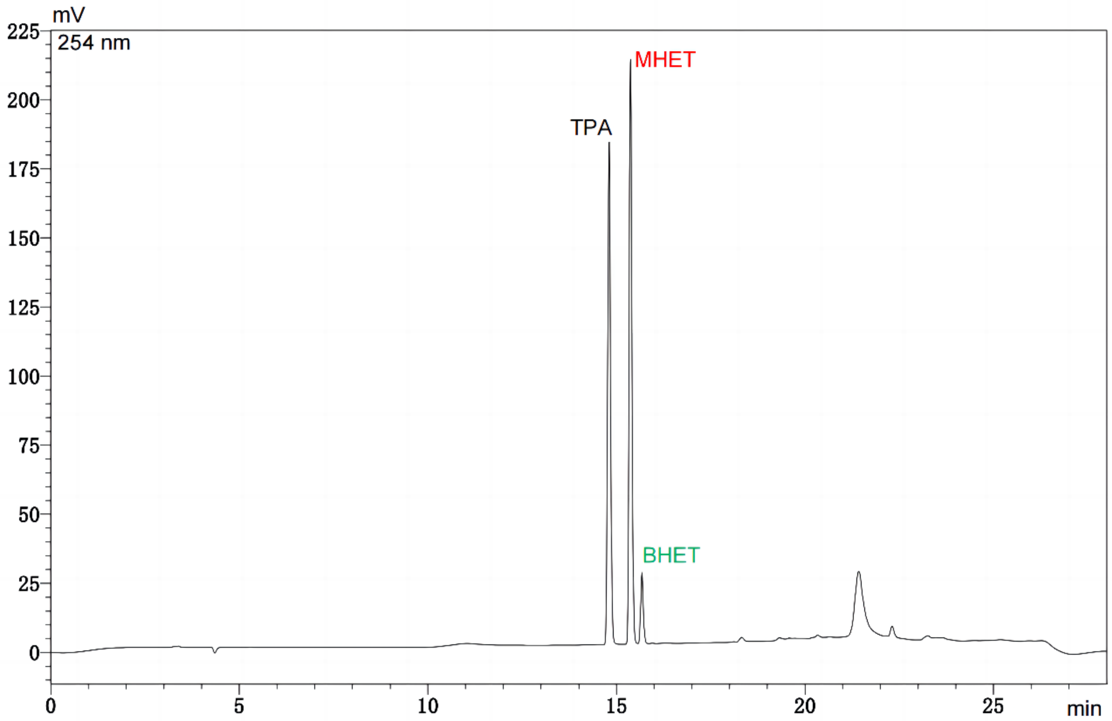

Fig. S25. HPLC chromatogram of the released products from PET degradation. The aromatic products were analyzed by CMB-20A (Shimadzu) connected to a UV/Vis detector (SPD-20A) and C18 column (SunFireTM C18, 5 µm, 4.6 x 250 mm) with a gradient of acetonitrile and 0.1% (v/v) formic acid in water at 30 °C after injection of 10 µL sample. Acetonitrile was increased from 5% to 44% until minute 13 and then to 70% at minute 18 where the ratio remained constant for 5 min.

Supplementary Methods

Computational enzyme design

The computational active site redesign of Est30 and recapitulation of the active sites of KLS (M14) in the crystal structure of KLS were carried out using the PRODA enzyme design protocol[23–26]. PRODA fully searched the huge conformational space of the protein and provided multiple computational mutant sequences of Est30 for further screening. Water molecules in native scaffold 1TQH were removed and the amino-acid hydrogen atoms were added using PRODA based on the topology parameters of the all-atom CHARMM 22 force field.[27] The atomic coordinate of MHET was taken from Pubchem (https://pubchem.ncbi.nlm.nih.gov), and hydrogen atoms were added using the molecular modeling software Discovery Studio. Eight sequence selection positions (T26, K122, M127, G130, L167, I171, M195, I196) were chosen to vary the amino acid types, into which substitutions to any of 17 residues (AGILMFWYVCSTRKHNQnaks) were simultaneously introduced. In addition, 13 positions (H23, N28, D31, L93, M115, C116, I121, S123, T126, V131, Q168, E194, V224), including 5 catalytic residues were subjected to alter rotameric states. The backbone atoms at the selected positions, and all atoms at other positions, were kept rigid and referenced as the scaffold template. A backbone-independent rotamer library[28] containing 11,810 original rotamers, was used to model side-chain conformations of design sites. The crystal conformation of the native amino acid at each design position was also considered as a rotamer. The rotamers for serine, threonine, and tyrosine were expanded as previously described due to the diversity configurations that the hydroxyl hydrogen atom could adopt[25]. The substrate structure of MHET (Figure S3) in the TS was built by changing the central ester atom so that it had an intermediate tetrahedron geometry. The atomic van der Waals parameters for MHET were obtained from the model molecules of the CHARMM 22 force field, and the atomic partial charges were assigned based on the PARSE models.[29] A library of tetrahedral TS conformers for MHET was generated and screened based in accordance with the placing rules (Table S5) and catalytic geometrical constraints (Table S6) using the previously proposed small-molecule placement approach.[30,31]

The protein-ligand interactions of complex system in PRODA were calculated using the Molecular Mechanics-Generalized Born/Surface Area free energy function, with a reference state

of both the scaffold template and isolated ligand in solvent[24]. The total energy was a linear combination of seven energy terms listed in Eq. 3:

$$\Delta G = E_{\mathrm{LJVDWAttr}} + E_{\mathrm{LinearVDWRep}} + E_{\mathrm{HB}} + E_{\mathrm{desolv}} + E_{\mathrm{SC}} + E_{\mathrm{hydrophobic}} + E_{\mathrm{entropy}} \quad (3)$$

The above seven energy items are the Lennard-Jones potential for van der Waals attractive interaction ($E_{\mathrm{LJVDWAttr}}$), the linearized van der Waals repulsive interaction ($E_{\mathrm{LinearVDWRep}}$), orientation dependent hydrogen bonding (E_{HB}), the desolvation energy of polar atoms (E_{desolv}), the screened Coulombic interaction (E_{SC}), the hydrophobic contribution of nonpolar atom burial ($E_{\mathrm{hydrophobic}}$), and the side chain entropy contribution term (E_{entropy}), respectively. The structure of recapitulation of KLS mutant and sequences with single, double and triple mutations corresponding to the global or near-global minimum energy conformations (GMEC) of the enzyme-TS complex system were obtained using the Dead-End Elimination/Linear Programming/Mixed Integer Linear Programming-based deterministic combinatorial optimization algorithm.[24,31]To adequately search the conformational and sequence space, a large number of sequences around the global minimum energy sequence were generated by restricting that a predefined number of rotamer types were different for any two sequences.[32] The free energy of the bound enzyme-TS complex system ($\Delta G_{\mathrm{bound}}$) was the sum of binding energy (ΔG_{bind}) and folding energy (ΔG_{fold}). The binding energy (ΔG_{bind}) was calculated as the energy difference between the bound enzyme-TS system and the unbound enzyme-TS system[12], as shown in Eq. 1:

$$\Delta G_{\mathrm{bind}} = \Delta G_{\mathrm{bound}} - \Delta G_{\mathrm{unbound}} \quad (1)$$

where $\Delta G_{\mathrm{bound}}$ and $\Delta G_{\mathrm{unbound}}$ are the free energies of the complex and the unbound enzyme-TS systems.

The folding energies are the free energies of the apo-form enzyme, equal to $\Delta G_{\mathrm{unbound}}$, as shown in Eq.2:

$$\Delta G_{f\mathrm{old}} = \Delta G_{\mathrm{unbound}} \quad (2)$$

The binding energy change upon mutation ($\Delta\Delta G_{\mathrm{bind,Mut}}$) is computed as the difference of the binding energies of the wild-type ($\Delta G_{\mathrm{bind,WT}}$) and mutant enzyme-TS system ($\Delta G_{\mathrm{bind,Mut}}$), as shown in Eq.3:

$$\Delta\Delta G_{\text{bind,Mut}} = \Delta G_{\text{bind,Mut}} - \Delta G_{\text{bind,WT}} \quad (3)$$

The folding energy charge upon mutation ($\Delta\Delta G_{\text{fold,Mut}}$) is computed as the difference of the folding energies of the wild-type ($\Delta G_{\text{fold,WT}}$) and mutant enzymes ($\Delta G_{\text{fold,Mut}}$), as shown in Eq.4:

$$\Delta\Delta G_{f\text{old,Mut}} = \Delta G_{f\text{old,Mut}} - \Delta G_{\text{fold,WT}} \quad (4)$$

The mutant sequences generated by PRODA were ranked in ascending order of the calculated free energy change of the bound enzyme-TS complex system ($\Delta\Delta G_{\text{bound,Mut}} = \Delta\Delta G_{\text{bind,Mut}} + \Delta\Delta G_{\text{fold,Mut}}$). Mutants were selected for MD simulations characterization abide by the criteria described in text. All calculations are performed on a computer cluster with 256 cores, where each core represents a 2.1 GHz CPU from a sub-cluster with 64 cores sharing 128 GB memory.

Molecular dynamics simulation

The MD simulations were carried out using GROMACS 2019.4[33]. The initial complex coordinates of the designs were copied from the results computed by PRODA. The initial complex coordinates of the fusion enzyme KL36F were copied from the results predicted by AlphaFold2. The topologies of all the proteins were generated by GROMACS using the CHARMM36 all-atom force field. The topology of the corresponding small molecule, i.e., the TS, was prepared using the online CGenFF program 1.0.0[34,35] and its atom types, bond parameters, and atomic partial charges were built to be consistent with the definitions of the CHARMM General Force Field 3.0.1.[36,37] The δ nitrogen of the catalytic histidine residue (His223) was protonated in accordance with the catalytic mechanism of an esterase. The new protein–TS complex structure was immersed in a dodecahedral box whose size was determined by setting the distance between the solute and the box to 10 Å, and the box was filled by the addition of approximately 12,000 explicit water molecules represented by the water model TIP3P.[38] The charge of the system was neutralized by adding an appropriate number of Na^+ or Cl^- counter ions to the solvent box.

Each simulation was independently initiated with energy minimization (EM) of the system using the steepest descent minimization algorithm, and EM continued until the potential energy was negative and the maximum force F_{max} was not greater than 1000 $kJ\cdot mol^{-1}\cdot nm^{-1}$. Next, a position-restrained NVT phase (NVT = constant temperature and constant volume) simulation was performed using simulated annealing to warm the system. For the 1, 5, 50 ns MD simulations, the

temperature was gradually increased from 0 to 343 K within 150, 150, or 350 ps, then stabilized at 343 K for a further 30 ps. Subsequently, the system underwent a position-restrained NPT (NPT = constant temperature and constant pressure) simulation of 100, 200, or 500 ps, and 1, 5, or 50 ns production MD simulation, respectively. The time step was set to 1 fs for NVT and 2 fs for NPT. Constant temperature was maintained using the velocity rescaling thermostat[39] and constant pressure was maintained using the Berendsen barostat[40,41]. In the position-restrained simulations and MD simulations, the LINCS algorithm was used to impose constraints on the bonds and angles of the complex. The Particle Mesh Ewald method[42,43] was used to model long-range electrostatic effects, and short-range van der Waals interactions were cut off at 12 Å. The differential equations of motion were integrated by a leap-frog algorithm. The 1, 5, and 50 ns MD simulations were repeated 10, 5, and 2 times, respectively; the time step was 2 fs, and the coordinates of all the atoms in the system were saved every 10 ps, which generated a total of 100 frames, 500 frames and 5000 frames, respectively.

Post-simulation data extraction was performed using the corresponding GROMACS program, the distances, angles, h-bonds and RMSD of residues or MHET in each snapshot were extracted using the program “gmx distance”, “gmx gangle”, “gmx hbond” and “gmx rms”, respectively. The frequency values in single simulation were calculated based on the following Eq. 1:

$$\text{Freq} = \frac{n}{N} \qquad (5)$$

n is number of snapshots where the correspond hydrogen bond formed, the distance or the RMSD of MHET met correspond criteria. N is the number of snapshots in single simulation. The criteria for hydrogen bonding are that the Donor–Hydrogen–Acceptor angle is greater than 120°, and the Donor–Acceptor length is less than 3.5Å. The feature values of simulations were calculated and analyzed by Python. Snapshots of enzyme interactions with MHET were analyzed and visualized using PyMOL 2.4.0 software (https://pymol.org/).

Supplementary References

1. Sagong, H.-Y. *et al.* Decomposition of the PET Film by MHETase Using Exo-PETase Function. *ACS Catal.* **10**, 4805–4812 (2020).
2. Knott, B. C. *et al.* Characterization and engineering of a two-enzyme system for plastics depolymerization. *Proc Natl Acad Sci USA* **117**, 25476–25485 (2020).
3. Rabbani, G. *et al.* Impact of structural stability of cold adapted Candida antarctica lipase B (CaLB): in relation to pH, chemical and thermal denaturation. *RSC Adv.* **5**, 20115–20131 (2015).
4. Carniel, A., Valoni, É., Nicomedes, J., Gomes, A. da C. & Castro, A. M. de. Lipase from Candida antarctica (CALB) and cutinase from Humicola insolens act synergistically for PET hydrolysis to terephthalic acid. *Process Biochemistry* **59**, 84–90 (2017).
5. Bååth, J. A., Borch, K., Jensen, K., Brask, J. & Westh, P. Comparative Biochemistry of Four Polyester (PET) Hydrolases**. *ChemBioChem* **22**, 1627–1637 (2021).
6. Mrigwani, A., Thakur, B. & Guptasarma, P. Conversion of polyethylene terephthalate into pure terephthalic acid through synergy between a solid-degrading cutinase and a reaction intermediate-hydrolysing carboxylesterase. *Green Chem.* 10.1039.D2GC01965E (2022) doi:10.1039/D2GC01965E.
7. Kim, H. T. *et al.* Chemo-Biological Upcycling of Poly(ethylene terephthalate) to Multifunctional Coating Materials. *ChemSusChem* **14**, 4251–4259 (2021).
8. Meyer-Cifuentes, I. E. & Öztürk, B. Mle046 Is a Marine Mesophilic MHETase-Like Enzyme. *Front. Microbiol.* **12**, 693985 (2021).
9. Palm, G. J. *et al.* Structure of the plastic-degrading Ideonella sakaiensis MHETase bound to a substrate. *Nat Commun* **10**, 1717 (2019).
10. Hedstrom, L. Serine Protease Mechanism and Specificity. *Chem. Rev.* **102**, 4501–4524 (2002).
11. Foglia, F. *et al.* Role of the N-terminal region for the conformational stability of esterase 2 from Alicyclobacillus acidocaldarius. *Biophysical Chemistry* **127**, 113–122 (2007).
12. Liu, P. *et al.* Covalent Reaction Intermediate Revealed in Crystal Structure of the Geobacillus stearothermophilus Carboxylesterase Est30. *Journal of Molecular Biology* **342**, 551–561 (2004).
13. Yang, S., Qin, Z., Duan, X., Yan, Q. & Jiang, Z. Structural insights into the substrate specificity of two esterases from the thermophilic Rhizomucor miehei. *Journal of Lipid Research* **56**, 1616–1624 (2015).
14. Sayer, C. *et al.* Structural and biochemical characterisation of Archaeoglobus fulgidus esterase reveals a bound CoA molecule in the vicinity of the active site. *Sci Rep* **6**, 25542 (2016).
15. Sayer, C., Isupov, M. N., Bonch-Osmolovskaya, E. & Littlechild, J. A. Structural studies of a thermophilic esterase from a new Planctomycetes species, *Thermogutta terrifontis*. *FEBS J* **282**, 2846–2857 (2015).
16. Byun, J.-S. *et al.* Crystal structure of hyperthermophilic esterase EstE1 and the relationship between its dimerization and thermostability properties. *BMC Struct Biol* **7**, 1–11 (2007).
17. De Santi, C. *et al.* Biochemical characterization and structural analysis of a new cold-active and salt-tolerant esterase from the marine bacterium Thalassospira sp. *Extremophiles* **20**, 323–336 (2016).
18. Spiller, B., Gershenson, A., Arnold, F. H. & Stevens, R. C. A structural view of evolutionary divergence. *Proceedings of the National Academy of Sciences* **96**, 12305–12310 (1999).
19. Lu, H. *et al.* Machine learning-aided engineering of hydrolases for PET depolymerization. *Nature* **604**, 662–667 (2022).
20. Tournier, V. *et al.* An engineered PET depolymerase to break down and recycle plastic bottles. *Nature* **580**, 216–219 (2020).
21. Madeira, F. *et al.* Search and sequence analysis tools services from EMBL-EBI in 2022. *Nucleic Acids Research* **50**, W276–W279 (2022).
22. Robert, X. & Gouet, P. Deciphering key features in protein structures with the new ENDscript server. *Nucleic Acids Research* **42**, W320–W324 (2014).
23. Lei, Y., Luo, W. & Zhu, Y. A matching algorithm for catalytic residue site selection in computational enzyme design. *Protein Science* **20**, 1566–1575 (2011).

24. Tian, Y., Huang, X. & Zhu, Y. Computational design of enzyme–ligand binding using a combined energy function and deterministic sequence optimization algorithm. *J Mol Model* **21**, 191 (2015).
25. Huang, X., Xue, J., Lin, M. & Zhu, Y. Use of an Improved Matching Algorithm to Select Scaffolds for Enzyme Design Based on a Complex Active Site Model. *PLoS ONE* **11**, e0156559 (2016).
26. Zhang, S., Zhang, J. & Zhu, Y. ProdaMatch: A fast and accurate active site matching algorithm for de novo enzyme design. *Computers & Chemical Engineering* **140**, 106921 (2020).
27. MacKerell, A. D. *et al.* All-Atom Empirical Potential for Molecular Modeling and Dynamics Studies of Proteins. *J. Phys. Chem. B* **102**, 3586–3616 (1998).
28. Xiang, Z. & Honig, B. Extending the accuracy limits of prediction for side-chain conformations. *Journal of Molecular Biology* **311**, 421–430 (2001).
29. Sitkoff, D., Sharp, K. A. & Honig, B. Accurate Calculation of Hydration Free Energies Using Macroscopic Solvent Models. *J. Phys. Chem.* **98**, 1978–1988 (1994).
30. Lassila, J. K., Privett, H. K., Allen, B. D. & Mayo, S. L. Combinatorial methods for small-molecule placement in computational enzyme design. *Proc. Natl. Acad. Sci. U.S.A.* **103**, 16710–16715 (2006).
31. Huang, X., Han, K. & Zhu, Y. Systematic optimization model and algorithm for binding sequence selection in computational enzyme design: Selection Algorithm for Enzyme Design. *Protein Science* **22**, 929–941 (2013).
32. Tian, Y., Huang, X., Li, Q. & Zhu, Y. Computational design of variants for cephalosporin C acylase from Pseudomonas strain N176 with improved stability and activity. *Appl Microbiol Biotechnol* **101**, 621–632 (2017).
33. Abraham, M. J. *et al.* GROMACS: High performance molecular simulations through multi-level parallelism from laptops to supercomputers. *SoftwareX* **1–2**, 19–25 (2015).
34. Vanommeslaeghe, K. *et al.* CHARMM general force field: A force field for drug-like molecules compatible with the CHARMM all-atom additive biological force fields. *J. Comput. Chem.* NA-NA (2009) doi:10.1002/jcc.21367.
35. Yu, W., He, X., Vanommeslaeghe, K. & MacKerell, A. D. Extension of the CHARMM general force field to sulfonyl-containing compounds and its utility in biomolecular simulations. *J. Comput. Chem.* **33**, 2451–2468 (2012).
36. Vanommeslaeghe, K., Raman, E. P. & MacKerell, A. D. Automation of the CHARMM General Force Field (CGenFF) II: Assignment of Bonded Parameters and Partial Atomic Charges. *J. Chem. Inf. Model.* **52**, 3155–3168 (2012).
37. Vanommeslaeghe, K. & MacKerell, A. D. Automation of the CHARMM General Force Field (CGenFF) I: Bond Perception and Atom Typing. *J. Chem. Inf. Model.* **52**, 3144–3154 (2012).
38. Jorgensen, W. L., Chandrasekhar, J., Madura, J. D., Impey, R. W. & Klein, M. L. Comparison of simple potential functions for simulating liquid water. *The Journal of Chemical Physics* **79**, 926–935 (1983).
39. Bussi, G., Donadio, D. & Parrinello, M. Canonical sampling through velocity rescaling. *The Journal of Chemical Physics* **126**, 014101 (2007).
40. Andersen, H. C. Molecular dynamics simulations at constant pressure and/or temperature. *The Journal of Chemical Physics* **72**, 2384–2393 (1980).
41. Berendsen, H. J. C., Postma, J. P. M., van Gunsteren, W. F., DiNola, A. & Haak, J. R. Molecular dynamics with coupling to an external bath. *The Journal of Chemical Physics* **81**, 3684–3690 (1984).
42. Essmann, U. *et al.* A smooth particle mesh Ewald method. *The Journal of Chemical Physics* **103**, 8577–8593 (1995).
43. Darden, T., York, D. & Pedersen, L. Particle mesh Ewald: An $N \cdot \log(N)$ method for Ewald sums in large systems. *The Journal of Chemical Physics* **98**, 10089–10092 (1993).
