# Supplementary material for: Computational design of highly efficient thermostable MHET hydrolases and dual enzyme system for PET recycling
Source: Commun Biol. 2023 Nov 9;6:1135. doi: 10.1038/s42003-023-05523-5 (PMC10636135; doi:10.1038/s42003-023-05523-5)
Supplement: Supplementary file 3 — Description of Supplementary Materials [file 42003_2023_5523_MOESM3_ESM.docx]

**Description of Additional Supplementary Files**

**File name:** Supplementary Data 1

**Description**: The source data behind the graphs in the paper

**File name:** Supplementary Data 2

**Description**: The data sets of Tables S9 and S10.
