# Supplementary material for: Computational design of highly efficient thermostable MHET hydrolases and dual enzyme system for PET recycling
Source: Commun Biol. 2023 Nov 9;6:1135. doi: 10.1038/s42003-023-05523-5 (PMC10636135; doi:10.1038/s42003-023-05523-5)
Supplement: Supplementary file 6 — Reporting Summary [file 42003_2023_5523_MOESM6_ESM.pdf]

nature portfolio

Corresponding author(s): Yushan Zhu

Last updated by author(s): Sep 27, 2023

Reporting Summary

Nature Portfolio wishes to improve the reproducibility of the work that we publish. This form provides structure for consistency and transparency in reporting. For further information on Nature Portfolio policies, see our Editorial Policies and the Editorial Policy Checklist.

Statistics

For all statistical analyses, confirm that the following items are present in the figure legend, table legend, main text, or Methods section.

n/a	Confirmed	
☐	☒	The exact sample size (*n*) for each experimental group/condition, given as a discrete number and unit of measurement
☐	☒	A statement on whether measurements were taken from distinct samples or whether the same sample was measured repeatedly
☒	☐	The statistical test(s) used AND whether they are one- or two-sided<br>*Only common tests should be described solely by name; describe more complex techniques in the Methods section.*
☒	☐	A description of all covariates tested
☒	☐	A description of any assumptions or corrections, such as tests of normality and adjustment for multiple comparisons
☐	☒	A full description of the statistical parameters including central tendency (e.g. means) or other basic estimates (e.g. regression coefficient) AND variation (e.g. standard deviation) or associated estimates of uncertainty (e.g. confidence intervals)
☒	☐	For null hypothesis testing, the test statistic (e.g. *F*, *t*, *r*) with confidence intervals, effect sizes, degrees of freedom and *P* value noted<br>*Give P values as exact values whenever suitable.*
☒	☐	For Bayesian analysis, information on the choice of priors and Markov chain Monte Carlo settings
☒	☐	For hierarchical and complex designs, identification of the appropriate level for tests and full reporting of outcomes
☒	☐	Estimates of effect sizes (e.g. Cohen's *d*, Pearson's *r*), indicating how they were calculated

Our web collection on statistics for biologists contains articles on many of the points above.

Software and code

Policy information about availability of computer code

Data collection	X-Ray data were collected at Shanghai 634 Synchrotron Radiation Facility beamlines BL02U1 and BL19U1.<br>LCsolution (Shimadzu) was used to record HPLC data.<br>MicroCal PEAQ-DSC (Malvern Panalytical) was used to record DSC data for the protein thermal stability test.<br>Pyris (PerkinElmer) was used to record DSC data for determining PET crystallinity.<br>AlphaFold2 and AlphaFold-Multimer were used to predict the structures of all dual enzyme systems.<br>The substrate structure of MHET in the TS was built using Discovery Studio 2018.<br>PRODA was used to generate candidate sequences and calculate the corresponding free energies.<br>GROMACS 2019.4 was used to perform the molecular dynamics simulations.
Data analysis	AlphaFold2, COOT and PHENIX were used to process and refine the crystallographic data.<br>LCsolution (Shimadzu) was used to analyze HPLC data for integration of chromatograms.<br>MicroCal PEAQ-DSC (Malvern Panalytical) was used to analyze DSC data and obtain the protein melting temperatures.<br>Pyris (PerkinElmer) was used to analyze DSC data for integration of heat flux in order to determine PET crystallinity.<br>Microsoft Excel 2016, Origin Pro 9.1, Python 3.7 were used to analyze and display all experimental and computational data.<br>PyMOL 2.4.0 was used for structure comparison and visualization.<br>GROMACS 2019.4 was used to analyze the post-simulation data.<br>Sequence alignment and display were carried out with Clustal Omega and ESPript.

For manuscripts utilizing custom algorithms or software that are central to the research but not yet described in published literature, software must be made available to editors and reviewers. We strongly encourage code deposition in a community repository (e.g. GitHub). See the Nature Portfolio guidelines for submitting code & software for further information.

Data

Policy information about availability of data

All manuscripts must include a data availability statement. This statement should provide the following information, where applicable:

- Accession codes, unique identifiers, or web links for publicly available datasets
- A description of any restrictions on data availability
- For clinical datasets or third party data, please ensure that the statement adheres to our policy

The atomic coordinates and structure factors have been deposited in the Protein Data Bank, (https://www.wwpdb.org/) with PDB code: 8ILT. AlphaFold models for enzyme complexes are available at https://github.com/zhangjun19thu/SI_Structures. Other data are provided with this paper. Additional supplementary data that support this study are available from the corresponding author (YZ) upon request.

Research involving human participants, their data, or biological material

Policy information about studies with human participants or human data. See also policy information about sex, gender (identity/presentation), and sexual orientation and race, ethnicity and racism.

Reporting on sex and gender	*Use the terms sex (biological attribute) and gender (shaped by social and cultural circumstances) carefully in order to avoid confusing both terms. Indicate if findings apply to only one sex or gender; describe whether sex and gender were considered in study design; whether sex and/or gender was determined based on self-reporting or assigned and methods used. Provide in the source data disaggregated sex and gender data, where this information has been collected, and if consent has been obtained for sharing of individual-level data; provide overall numbers in this Reporting Summary. Please state if this information has not been collected. Report sex- and gender-based analyses where performed, justify reasons for lack of sex- and gender-based analysis.*
Reporting on race, ethnicity, or other socially relevant groupings	*Please specify the socially constructed or socially relevant categorization variable(s) used in your manuscript and explain why they were used. Please note that such variables should not be used as proxies for other socially constructed/relevant variables (for example, race/ethnicity should not be used as a proxy for socioeconomic status). Provide clear definitions of the relevant terms used, how they were provided (by the participants/respondents, the researchers, or third parties), and the method(s) used to classify people into the different categories (e.g. self-report, census or administrative data, social media data, etc.) Please provide details about how you controlled for confounding variables in your analyses.*
Population characteristics	*Describe the covariate-relevant population characteristics of the human research participants (e.g. age, genotypic information, past and current diagnosis and treatment categories). If you filled out the behavioural & social sciences study design questions and have nothing to add here, write "See above."*
Recruitment	*Describe how participants were recruited. Outline any potential self-selection bias or other biases that may be present and how these are likely to impact results.*
Ethics oversight	*Identify the organization(s) that approved the study protocol.*

Note that full information on the approval of the study protocol must also be provided in the manuscript.

Field-specific reporting

Please select the one below that is the best fit for your research. If you are not sure, read the appropriate sections before making your selection.

☒ Life sciences ☐ Behavioural & social sciences ☐ Ecological, evolutionary & environmental sciences

For a reference copy of the document with all sections, see nature.com/documents/nr-reporting-summary-flat.pdf

Life sciences study design

All studies must disclose on these points even when the disclosure is negative.

Sample size	For enzymatic activity assays and the experiments incorporating various enzymes and cell, triplicates were performed to determine mean and standard deviation. For melting temperature and PET crystallinity assessments, duplicates were performed. Results are reproducible. Attempts at replication were successful.
Data exclusions	No data was excluded.
Replication	All in vitro experiments with explicit standard deviation (SD) were performed in duplicates or triplicates where feasible. Attempts at replication were successful.
Randomization	No data was randomized since it was not applicable for our set of experiments.
Blinding	For enzymatic activity measurements, DSC, HPLC analysis, the analytical team who performed the experiments and analyzed the data were

Blinding	blind to the samples they were handling with.

Reporting for specific materials, systems and methods

We require information from authors about some types of materials, experimental systems and methods used in many studies. Here, indicate whether each material, system or method listed is relevant to your study. If you are not sure if a list item applies to your research, read the appropriate section before selecting a response.

Materials & experimental systems

n/a	Involved in the study
☒	☐ Antibodies
☒	☐ Eukaryotic cell lines
☒	☐ Palaeontology and archaeology
☒	☐ Animals and other organisms
☒	☐ Clinical data
☒	☐ Dual use research of concern
☒	☐ Plants

Methods

n/a	Involved in the study
☒	☐ ChIP-seq
☒	☐ Flow cytometry
☒	☐ MRI-based neuroimaging
